# Unveiling the Relationship Between Oral Microbiota and Alzheimer's Disease: A Genetic Instrumental Variable Analysis via Mendelian Randomization

**DOI:** 10.1002/brb3.70753

**Published:** 2025-08-04

**Authors:** Zhichao Li, Yihan Kang, Shuai Li, Shiqi Guo, Hui Zheng

**Affiliations:** ^1^ Department of Anesthesiology, National Cancer Center/National Clinical Research Center for Cancer/Cancer Hospital Chinese Academy of Medical Sciences and Peking Union Medical College Beijing China; ^2^ Department of Anesthesiology The First Hospital of China Medical University Shenyang China; ^3^ Department of General Surgery, National Center of Gerontology, Institute of Geriatric Medicine/Chinese Academy of Medical Sciences & Peking Union Medical College Beijing Hospital Beijing China

**Keywords:** Alzheimer's disease, causal relationship, genetic analysis, Mendelian randomization, oral microbiota, single nucleotide polymorphisms

## Abstract

**Background**: The potential relationship between oral microbiota (OM) and Alzheimer's disease (AD) is increasingly recognized, but the exact causal relationship between them remains uncertain. This study aims to reveal the causal relationship between OM and AD.

**Methods**: A two‐sample Mendelian randomization (MR) approach was employed to examine the association between 594 OM exposures and AD outcomes. Effect estimates were derived from external genome‐wide association study (GWAS) summary statistics, primarily utilizing inverse‐variance weighted (IVW) analysis. Sensitivity analyses were conducted to assess the robustness of the findings. In addition, we genetically mapped SNPs corresponding to OM in the MR analysis to identify genes that may link OM to AD.

**Results**: A total of 48 OM exposures exhibited statistically significant associations with AD outcomes (*p* ≤ 0.05). Of these, 30 were identified at the genus level, 12 at the species level, and six at the family level. Genetic function analyses indicated that OM‐related genes are closely linked to the regulation of neurobiological functions, supporting a potential role for OM in the pathogenesis of AD.

**Conclusion**: The findings presented here provide genetic evidence for a causal relationship between OM and AD, offering insights that may guide the future development of prevention and treatment strategies targeting OM in the context of AD.

## Graphical abstract

This study investigated the association between oral microbiota (OM) and Alzheimer's disease (AD). The causal relationship between OM and AD was established using Mendelian randomization (MR) methods and further supported by gene function analyses.

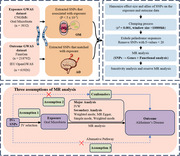

## 1 Introduction

Alzheimer's disease (AD) is a common neurodegenerative disease in older individuals and the most common type of dementia. AD is primarily characterized by a gradual decline in cognitive function and the loss of specific neurons and synapses. Initially, the disease presents with subtle brain changes. However, as neurons become damaged or partially destroyed, symptoms such as memory loss, cognitive impairment, and language deficits emerge gradually (Scheltens et al. 2021). The predominant pathological hallmarks in AD are the deposition of amyloid plaques and the formation of neurofibrillary tangles (Jucker and Walker 2023). Current consensus suggests that the interplay between genetic predisposition and environmental influences constitutes a primary precipitant in the pathogenesis of AD (Migliore and Coppedè 2022).

The oral microbiota (OM) is intricately shaped by the unique physiology of the oral cavity and has co‐evolved alongside its host over time. This evolutionary relationship allows the host to provide a stable ecological niche for symbiotic bacteria. In turn, the OM fosters local health by forming beneficial biofilms that help maintain pH balance and inhibit pathogenic growth. Moreover, it plays a vital role in enhancing systemic physiological processes, including the homeostasis of the cardiovascular and nervous systems (Tonelli et al. 2023; Botelho et al. 2022; Gao et al. 2018). However, when these biofilms become dysregulated and disrupt the host's homeostatic balance, the OM can become a reservoir for opportunistic pathogens. Such pathogens are implicated in the pathogenesis of various diseases, including inflammatory bowel disease, arthritis, cancer, and AD (Tuganbaev et al. 2022; Jungbauer et al. 2022; Yang et al. 2021; Z. Li et al. 2023).

Mendelian randomization (MR) is an advanced epidemiological technique that leverages genetic variation to assess the causal impact of modifiable risk factors on health outcomes. Based on Mendelian inheritance principles, MR mirrors randomization in controlled trials, providing a robust framework for establishing causal relationships (D. Tian, Zhang, et al. 2022; Z. Li et al. 2025). This approach is particularly useful when randomized controlled trials are impractical or when observational studies face confounding or reverse causality. By using single nucleotide polymorphisms (SNPs) as instrumental variables (IVs), MR enables researchers to infer the causal effects of specific exposures on outcomes, effectively addressing limitations inherent in observational studies (Sekula et al. 2016).

Recent studies using MR have established a causal link between gut microbiota and several neurodegenerative disorders, including major depression, delirium, Parkinson's disease, and AD (Ji et al. 2024; M. Chen et al. 2022; Jiang et al. 2023; Yu et al. 2023). C. Li et al. (2022) further investigated the relationship between OM and conditions like anxiety and depression through MR. Research on the relationship between OM and AD has primarily depended on preclinical and observational studies, which are significantly affected by confounding factors and inherent limitations, thus undermining the reliability of the findings (Cirstea et al. 2022; Jungbauer et al. 2022; Y.‐F. Wu, Lee, et al. 2021). This study aims to investigate the association between OM and AD using MR for the first time, assessing the potential causal relationship and impact of OM on AD risk.

## 2 Materials and Methods

### 2.1 Study Design

**FIGURE 1:**
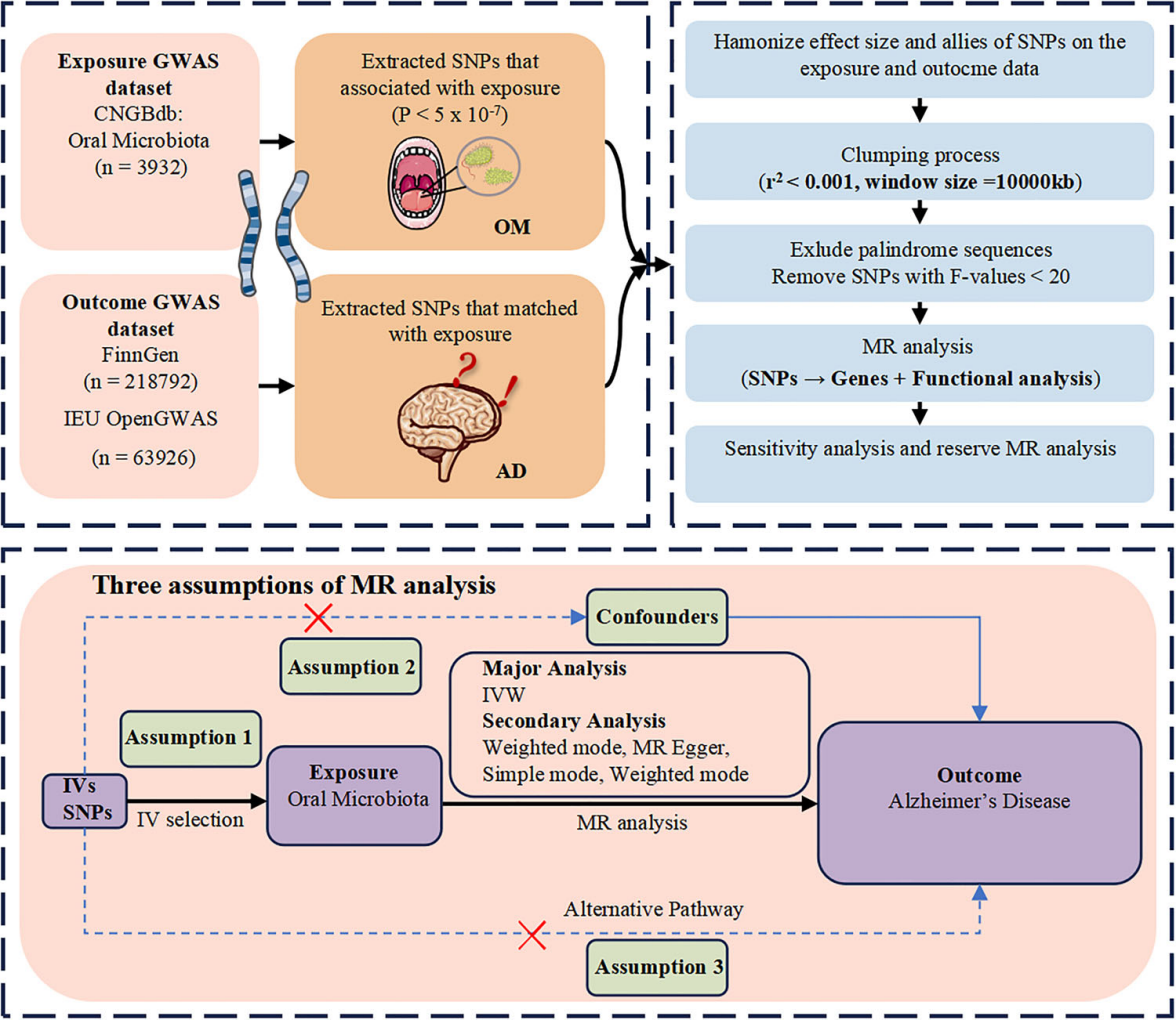
Overview of study design and MR strategy. The solid line illustrates the direct pathway through which genetic variation acts as an instrument for the risk factor under investigation. The dashed lines signify potential causal associations between variables that could undermine the MR hypothesis. (1) Assumption 1, which posits strong associations between the IVs and OM; (2) Assumption 2, which asserts that the IVs are unrelated to confounding factors; (3) Assumption 3, which states that the IVs influence AD solely through OM. AD, Alzheimer's Disease; GWAS, Genome‐Wide Association Study; IV_S_, instrumental variables; IVW, inverse‐variance weighted; MR, Mendelian Randomization; OM, Oral Microbiota; SNPs: Single nucleotide polymorphisms.

This study examined 594 OM exposures (*n* = 3932), including 309 from the tongue dorsum and 285 from saliva, with AD as the outcome (*n* = 282,718). We employed a two‐sample MR methodology to investigate the causal relationship, utilizing data from independent GWAS to enhance efficiency and robustness compared to single‐sample MR. To ensure the validity of our analysis, we adhered to three fundamental assumptions of MR (Figure 1).

### 2.2 Oral Microbiota

OM data, encompassing samples from saliva and the tongue dorsum, were sourced from the China National GeneBank Database (CNGBdb), which comprises microbiota profiles from 2984 healthy Chinese individuals. Shotgun metagenomic sequencing was performed using the BGISEQ‐500 platform with paired‐end 100 bp reads. Sequencing quality control was rigorously applied, with filtering to ensure high‐quality data (mean quality Phred score ≥ 20, minimum read length ≥ 51 bp). The microbiome composition was determined by aligning the sequencing reads to a reference catalog of oral genomes, and the relative abundance of each species was quantified. The analysis pipeline utilized Bowtie2 for read alignment, followed by normalization of contig depths to generate species‐level abundance profiles. In this study, we analyzed MR using summary data from a previous GWAS based on these samples. This GWAS marks the first large‐scale investigation of its kind within this population, analyzing a total of 2017 tongue dorsum samples and 1915 saliva samples through high‐depth whole‐genome sequencing (Liu et al. 2021).

### 2.3 Alzheimer's Disease

This study employed two extensive datasets to explore the causal relationship between OM and AD. The AD findings extracted from the ieu‐b‐2 dataset encompass genetic data from a substantial European cohort [PMID 30820047], while the finn‐b‐G6_ALZHEIMER dataset includes information from a Finnish genetic population. Incorporating diverse populations enhances our understanding of the influence of OM on AD across varying genetic and environmental backgrounds. A summary of data sources for this study is provided in Table 1.

**TABLE 1:** Summary of the data source in the study.

Trait	Consortium	Samples	Case	Control
**Exposure**				
Oral microbiota	CNGBdb	3932 (Tongue. *n* = 2017, saliva. *n* = 1915)	—	—
**Outcome**				
Alzheimer's disease	ieu‐b‐2	63,926	21,982	41,944
Alzheimer's disease	finn‐b‐G6_ALZHEIMER	218,792	3899	214,893

Abbreviations: CNGBdb, China National GeneBank DataBase; finn, Finnish Genetics; ieu, Integrative Epidemiology Unit; MR, Mendelian Randomization.

### 2.4 Instrumental Variable Selection

SNPs significantly associated with OM (*p* < 5.0 × 10^−7^) were selected as IVs for the MR analyses. These SNPs were chosen based on their relevance to the OM of interest and their availability in both datasets. To ensure the validity of the IVs, we excluded SNPs with pleiotropic effects unrelated to microbiota. We excluded linked unbalanced SNPs to minimize the risk of alleles from multiple loci co‐occurring on a single chromosome (*r* ^2^ < 0.001, kb = 10,000). Palindromic SNPs were also discarded to maintain alignment between effect alleles and their effect sizes. Furthermore, SNPs with *F*‐statistic below 20 were excluded, as higher *F*‐statistic indicate a reduced likelihood of weak instrumental bias. The *F*‐statistic was calculated using *F* = (*R* ^2^/[1 − *R* ^2^]) *([*N *− *K *− 1]/*K*), where *R* ^2^ = 2 × EAF × (1 − EAF) × β^2^ (EAF represents effect allele frequency, *N* is the sample size, and *K* is the number of IVs). Finally, each SNP was rigorously assessed in PhenoScanner (http://www.phenoscanner.medschl.cam.ac.uk/) to identify potential confounding factors, ensuring that the IVs influenced AD solely through OM.

### 2.5 Mendelian Randomization Analysis

A two‐sample MR analysis was conducted to investigate the causal relationships between specific OM and the risk of AD. The inverse‐variance weighted (IVW) method served as the primary analytical approach, augmented by MR‐Egger regression, weighted median, simple median, and weighted mode methods to address potential pleiotropy and enhance the robustness of our estimates (Supporting Information S1). The IVW method aggregates effect estimates from the IVs under the assumption of no pleiotropy. To further validate our findings, we conducted heterogeneity and pleiotropy analyses. Cochran's *Q* test was utilized to assess the heterogeneity of individual causal effects, and the MR‐Egger intercept test was applied to evaluate horizontal pleiotropy.

### 2.6 SNP‐Gene Mapping and Analysis of Key Genes

In addition, we utilized the online database SNPnexus (https://www.snp‐nexus.org/v4/) as a variant annotation tool to associate each SNP linked to microbiota risk or protective effects with its nearest gene, including overlapping, downstream, or upstream genes (Oscanoa et al. 2020). We conducted functional enrichment analyses on genes identified through SNPs mapping related to OM. Gene ontology (GO) and *Kyoto Encyclopedia of Genes and Genomes* (KEGG) pathway analyses were performed using the “clusterProfiler” R package (T. Wu, Hu, et al. 2021), with a significance threshold of *p* < 0.05. We visualized the three most significant GO terms and pathways (or all terms if fewer than three met this criterion) using the “ggplot2” R package.

### 2.7 Statistical Analysis

All statistical analyses were performed in R Studio (R version 4.2.3), employing the “TwoSampleMR” and “MR‐PRESSO” (Mendelian Randomization Pleiotropy RESidual Sum and Outlier) packages, which are specifically tailored for MR Analysis. The significant threshold for these analyses was established at *p* value = 0.05. The detailed statistical content is described as follows: (1) *IVW analysis*: IVW was employed to estimate the overall effect of OM on AD outcomes by performing a meta‐analysis of the variance‐weighted inverse mean of all SNPs effect estimates (Cui and Tian 2021). Without pleiotropy and heterogeneity, a significant IVW result (*p* ≤ 0.05) can be considered reliable, even if other methods do not yield significant results (X. Chen et al. 2020). (2) *Sensitivity analysis*: To verify the robustness of the IVW model, we conducted additional analyses using the MR‐Egger, weighted median, simple model, and weighted model methods (Mavromatis et al. 2022). The results were illustrated with scatter plots (Supporting Information S2). (3) *Pleiotropy analysis*: The MR‐Egger method detected potential pleiotropy in the SNPs. The MR‐Egger intercept analysis can elucidate this potential pleiotropy; if the intercept differs from zero (*p* < 0.05), the IVs are considered to exhibit horizontal pleiotropy, implying that the results persist regardless of exposure (Bowden et al. 2015; Yoshiji et al. 2023). (4) *Heterogeneity test*: Cochran's *Q* test quantified heterogeneity (*p* < 0.05 indicates presence) (Cohen et al. 2015), with the results visualized by funnel plot symmetry (Supporting Information S3). (5) *Leave‐One‐Out analysis*: This analysis was utilized to assess the impact of individual SNPs on the results (Burgess et al. 2017). By excluding one SNP at a time and analyzing the remaining SNPs using MR, we confirmed the influence of outlier variants on the results (Supporting Information S4).

## 3 Results

### 3.1 Associations Between Oral Microbiota and Alzheimer's Disease

The MR analysis identified statistically significant associations (*p* ≤ 0.05) between 48 specific OM taxa and the risk of AD, including 30 taxa identified at the genus level, 12 at the species level, and six at the family level. These findings were primarily derived using the IVW method and further validated through several complementary MR analyses, each providing odds ratios (OR) and 95% confidence intervals (CIs) to illustrate the associations between OM and AD (Table 2).

**TABLE 2:** Associations between oral microbiota and Alzheimer's disease risk.

Outcome (ID)	Exposure (ID)	Exposure (microbiotia)	SNP (*n*)	Method	*β*	SE	OR	95% CI	*p* value
AD	Saliva microbiotia								
finn‐b‐G6_ALZHEIMER	pheno.1304	*Streptococcus* *vestibularis* ^s^	4	IVW	−0.384	0.185	0.681	0.474–0.978	0.038
				MR‐Egger	5.373	9.200	2.16E + 02	0.000–1.46E+10	0.618
				Weighted median	−0.406	0.174	0.667	0.474–0.937	0.020
finn‐b‐G6_ALZHEIMER	pheno.1326	*Centipeda periodontii* ^s^	3	IVW	0.514	0.236	1.672	1.053–2.652	0.029
				MR‐Egger	6.469	34.600	6.45E + 02	0.000–1.83E + 32	0.882
				Weighted median	0.515	0.274	1.674	0.979–2.863	0.060
finn‐b‐G6_ALZHEIMER	pheno.1384	RUG343^g^	3	IVW	0.381	0.151	1.463	1.089–1.967	0.012
				MR‐Egger	−3.450	6.408	0.032	0.000–9.05E + 03	0.686
				Weighted median	0.341	0.181	1.407	0.987–2.005	0.059
finn‐b‐G6_ALZHEIMER	pheno.3524	*Lancefieldella* sp000564995^s^	5	IVW	−0.407	0.135	0.666	0.511–0.868	**0.003^※^**
				MR‐Egger	−8.282	24.718	0.000	0.000–2.78E + 17	0.760
				Weighted median	−0.272	0.151	0.762	0.567–1.024	0.071
ieu‐b‐2	pheno.1000	*Streptococcus infantis* ^s^	4	IVW	−0.233	0.119	0.792	0.628–1.000	0.050
				MR‐Egger	1.576	2.415	4.837	0.043–549.761	0.581
				Weighted median	−0.285	0.127	0.752	0.587–0.963	0.024
ieu‐b‐2	pheno.1035	*Neisseria* ^g^	6	IVW	0.194	0.087	1.214	1.023–1.441	0.026
				MR‐Egger	1.995	2.533	7.350	0.051–1052.101	0.475
				Weighted median	0.163	0.111	1.177	0.947–1.463	0.143
ieu‐b‐2	pheno.1048	CAG‐793^g^	3	IVW	−0.298	0.133	0.742	0.572–0.963	0.025
				MR‐Egger	−1.009	5.870	0.365	0.000–3.62E + 04	0.892
				Weighted median	−0.324	0.164	0.723	0.524–0.998	0.049
ieu‐b‐2	pheno.1062	*Haemophilus parainfluenzae* ^s^	3	IVW	0.200	0.100	1.222	1.003–1.487	0.046
				MR‐Egger	0.885	2.399	2.424	0.022–267.249	0.775
				Weighted median	0.190	0.121	1.209	0.953–1.534	0.117
ieu‐b‐2	pheno.1132	*Alloprevotella* ^g^	4	IVW	0.265	0.106	1.303	1.058–1.605	0.013
				MR‐Egger	−6.685	6.686	0.001	0.000–613.341	0.423
				Weighted median	0.195	0.147	1.215	0.911–1.621	0.184
ieu‐b‐2	pheno.1346	*Campylobacter rectus* ^s^	4	IVW	0.205	0.087	1.227	1.035–1.455	0.018
				MR‐Egger	−3.036	4.157	0.048	0.000–165.913	0.541
				Weighted median	0.172	0.113	1.187	0.951–1.482	0.130
ieu‐b‐2	pheno.1499	UBA6648^g^	3	IVW	−0.280	0.105	0.756	0.615–0.929	**0.008^※^**
				MR‐Egger	−0.538	2.829	0.584	0.002–149.609	0.880
				Weighted median	−0.273	0.128	0.761	0.592–0.978	0.033
ieu‐b‐2	pheno.1535	*Treponema* ^g^	5	IVW	0.173	0.074	1.188	1.028–1.374	0.020
				MR‐Egger	1.371	0.998	3.941	0.557–27.868	0.263
				Weighted median	0.155	0.094	1.168	0.971–1.406	0.100
ieu‐b‐2	pheno.190	*Leptotrichia* *massiliensis* ^s^	3	IVW	0.228	0.112	1.257	1.009–1.565	0.041
				MR‐Egger	−13.197	10.701	0.000	0.000–2387.508	0.434
				Weighted median	0.145	0.135	1.156	0.887–1.506	0.284
ieu‐b‐2	pheno.2035	*Granulicatella* ^g^	5	IVW	−0.169	0.080	0.845	0.723–0.988	0.035
				MR‐Egger	0.623	1.848	1.865	0.050–69.780	0.758
				Weighted median	−0.192	0.105	0.825	0.671–1.014	0.068
ieu‐b‐2	pheno.214	Prevotella conceptionensis^s^	5	IVW	0.253	0.089	1.288	1.082–1.534	**0.005^※^**
				MR‐Egger	4.608	4.283	1.00E + 02	0.023–4.43E + 05	0.361
				Weighted median	0.241	0.117	1.272	1.012–1.599	0.039
ieu‐b‐2	pheno.2176	*Solobacterium* ^g^	4	IVW	0.232	0.104	1.261	1.029–1.546	0.025
				MR‐Egger	16.172	12.328	1.06E + 07	0.000–3.29E + 17	0.320
				Weighted median	0.290	0.110	1.336	1.076–1.659	0.009
ieu‐b‐2	pheno.2184	*Granulicatella* ^g^	4	IVW	0.232	0.102	1.261	1.032–1.541	0.023
				MR‐Egger	−1.093	1.746	0.335	0.011–10.264	0.595
				Weighted median	0.228	0.127	1.257	0.981–1.610	0.071
ieu‐b‐2	pheno.2292	*Lachnoanaerobaculum* sp000287675^s^	4	IVW	0.281	0.109	1.325	1.070–1.641	**0.010^※^**
				MR‐Egger	11.650	14.767	1.15E + 05	0.000–4.26E + 17	0.513
				Weighted median	0.279	0.134	1.321	1.017–1.717	0.037
ieu‐b‐2	pheno.2861	*Solobacterium* ^g^	4	IVW	−0.483	0.215	0.617	0.404–0.940	0.025
				MR‐Egger	−22.116	6.128	0.000	0.000–0.000	0.069
				Weighted median	−0.165	0.213	0.848	0.559–1.286	0.437
ieu‐b‐2	pheno.2891	*Lachnoanaerobaculum* ^g^	3	IVW	−0.303	0.119	0.739	0.585–0.932	0.011
				MR‐Egger	−3.986	4.813	0.019	0.000–232.365	0.560
				Weighted median	−0.300	0.146	0.741	0.556–0.987	0.040
ieu‐b‐2	pheno.2968	*Solobacterium* ^g^	4	IVW	−0.305	0.107	0.737	0.598–0.908	0.004^**^
				MR‐Egger	−1.633	5.247	0.195	0.000–5714.611	0.785
				Weighted median	−0.288	0.132	0.750	0.579–0.971	0.029
ieu‐b‐2	pheno.3024	*Solobacterium* ^g^	4	IVW	0.169	0.082	1.184	1.009–1.389	0.039
				MR‐Egger	−5.973	3.495	0.003	0.000–2.404	0.230
				Weighted median	0.229	0.106	1.258	1.022–1.547	0.030
ieu‐b‐2	pheno.3153	*Pauljensenia* sp000308055^s^	4	IVW	−0.261	0.112	0.771	0.619–0.959	0.020
				MR‐Egger	5.233	5.167	1.87E + 02	0.007–4.68E + 06	0.418
				Weighted median	−0.237	0.124	0.789	0.619–1.006	0.056
**ieu‐b‐2**	pheno.3275	*Saccharimonadaceae* TM7x^g^	3	IVW	−0.371	0.111	0.690	0.555–0.858	0.001^*^
				MR‐Egger	2.420	5.025	1.12E + 01	0.001–2.13E + 05	0.714
				Weighted median	−0.348	0.154	0.706	0.522–0.955	0.024
ieu‐b‐2	pheno.3362	*Saccharimonadaceae* ^f^	8	IVW	0.110	0.054	1.117	1.005–1.241	0.040
				MR‐Egger	1.381	1.428	3.978	0.242–65.381	0.371
				Weighted median	0.132	0.071	1.141	0.993–1.311	0.062
ieu‐b‐2	pheno.464	*Streptococcus* ^g^	4	IVW	0.332	0.138	1.394	1.063–1.826	0.016
				MR‐Egger	−1.958	6.725	0.141	0.000–7.49E + 04	0.798
				Weighted median	0.179	0.171	1.195	0.856–1.670	0.295
ieu‐b‐2	pheno.630	*Veillonella* ^g^	4	IVW	−0.321	0.083	0.725	0.616–0.854	0.000^*^
				MR‐Egger	−6.983	8.580	0.001	0.000–1.87E + 04	0.501
				Weighted median	−0.292	0.109	0.747	0.603–0.925	0.008
ieu‐b‐2	pheno.684	*Streptococcus* ^g^	3	IVW	0.190	0.090	1.209	1.013–1.443	0.036
				MR‐Egger	−9.986	13.914	0.000	0.00–3.22E + 07	0.604
				Weighted median	0.172	0.113	1.188	0.953–1.481	0.126
ieu‐b‐2	pheno.743	CAG‐793^g^	4	IVW	0.208	0.091	1.232	1.031–1.472	0.022
				MR‐Egger	−1.295	3.037	0.274	0.001–105.326	0.711
				Weighted median	0.189	0.118	1.208	0.959–1.520	0.108
ieu‐b‐2	pheno.828	*Aggregatibacter* ^g^	5	IVW	0.151	0.069	1.163	1.016–1.332	0.029
				MR‐Egger	1.899	1.512	6.680	0.345–129.370	0.298
				Weighted median	0.129	0.086	1.138	0.961–1.348	0.134
ieu‐b‐2	pheno.879	*Alloprevotella tannerae* ^s^	3	IVW	0.381	0.176	1.463	1.037–2.064	0.030
				MR‐Egger	−9.144	6.762	0.000	0.000–60.887	0.405
				Weighted median	0.328	0.197	1.388	0.943–2.043	0.097
**AD**	**Tongue microbiota**								
finn‐b‐G6_ALZHEIMER	pheno.738	*Saccharimonadaceae* ^f^	5	IVW	−0.344	0.145	0.709	0.534–0.941	0.017
				MR‐Egger	0.652	4.050	1.919	0.001–5.38E + 03	0.882
				Weighted median	−0.324	0.186	0.723	0.503–1.041	0.081
ieu‐b‐2	pheno.1303	*Streptobacillus* ^g^	5	IVW	−0.251	0.123	0.778	0.611–0.991	0.042
				MR‐Egger	−2.472	3.490	0.084	0.000–78.953	0.530
				Weighted median	−0.197	0.159	0.821	0.601–1.122	0.216
ieu‐b‐2	pheno.1644	*Bacteroidales* F082^f^	3	IVW	−0.239	0.111	0.787	0.633–0.979	0.032
				MR‐Egger	2.142	2.771	8.518	0.037–1946.987	0.581
				Weighted median	−0.257	0.133	0.773	0.596–1.003	0.052
ieu‐b‐2	pheno.1908	*Porphyromonas* ^g^	4	IVW	0.325	0.155	1.384	1.021–1.878	0.036
				MR‐Egger	−17.186	12.794	0.000	0.000–2673.004	0.311
				Weighted median	0.396	0.157	1.486	1.092–2.023	0.012
ieu‐b‐2	pheno.2065	*Aggregatibacter* ^g^	3	IVW	−0.270	0.117	0.763	0.607–0.959	0.021
				MR‐Egger	2.962	7.883	19.343	0.000–9.93E + 07	0.771
				Weighted median	−0.246	0.138	0.782	0.597–1.024	0.074
ieu‐b‐2	pheno.2167	*Neisseria* ^g^	3	IVW	−0.409	0.132	0.664	0.513–0.861	0.002^**^
				MR‐Egger	0.058	12.733	1.060	0.000–7.31E + 10	0.997
				Weighted median	−0.390	0.181	0.677	0.475–0.965	0.031
ieu‐b‐2	pheno.2219	*Campylobacter* ^g^	3	IVW	−0.278	0.109	0.757	0.612–0.937	0.011
				MR‐Egger	−1.455	3.352	0.233	0.000–166.579	0.739
				Weighted median	−0.342	0.138	0.711	0.542–0.931	0.013
ieu‐b‐2	pheno.2566	*Treponema vincentii* ^s^	3	IVW	−0.494	0.252	0.610	0.372–1.000	0.050
				MR‐Egger	13.455	13.470	6.98E + 05	0.000–2.04E + 17	0.500
				Weighted median	−0.476	0.312	0.621	0.337–1.145	0.127
ieu‐b‐2	pheno.2598	*Eubacterium* ^g^	3	IVW	0.251	0.103	1.285	1.049–1.573	0.015
				MR‐Egger	0.189	4.478	1.208	0.000–7830.512	0.973
				Weighted median	0.252	0.126	1.287	1.005–1.648	0.045
ieu‐b‐2	pheno.2994	CAG‐793^g^	5	IVW	0.185	0.092	1.204	1.005–1.441	0.044
				MR‐Egger	−5.437	8.217	0.004	0.000–4.29E + 04	0.555
				Weighted median	0.168	0.115	1.183	0.944–1.482	0.144
ieu‐b‐2	pheno.3149	*Saccharimonadaceae* ^f^	4	IVW	−0.204	0.079	0.815	0.699–0.952	0.010^**^
				MR‐Egger	1.404	3.153	4.071	0.008–1967.481	0.700
				Weighted median	−0.201	0.099	0.818	0.673–0.993	0.042
ieu‐b‐2	pheno.3255	*Saccharimonadaceae* TM7x^g^	6	IVW	−0.172	0.076	0.842	0.726–0.976	0.023
				MR‐Egger	−1.248	1.396	0.287	0.019–4.424	0.422
				Weighted median	−0.102	0.100	0.903	0.743–1.097	0.304
ieu‐b‐2	pheno.3515	*Saccharimonadaceae* ^f^	4	IVW	−0.182	0.093	0.834	0.696–1.000	0.050
				MR‐Egger	0.306	1.199	1.358	0.130–14.241	0.822
				Weighted median	−0.118	0.118	0.888	0.706–1.119	0.314
ieu‐b‐2	pheno.3794	*Saccharimonadaceae* ^f^	6	IVW	0.183	0.073	1.201	1.040–1.387	0.013
				MR‐Egger	−0.942	2.212	0.390	0.005–29.731	0.692
				Weighted median	0.184	0.092	1.202	1.005–1.438	0.044
ieu‐b‐2	pheno.429	*Fusobacterium* ^g^	3	IVW	0.300	0.135	1.350	1.036–1.759	0.026
				MR‐Egger	5.793	3.036	328.066	0.854–1.26E + 05	0.307
				Weighted median	0.260	0.141	1.297	0.985–1.708	0.064
ieu‐b‐2	pheno.790	*Streptococcus* ^g^	5	IVW	0.203	0.099	1.225	1.010–1.486	0.039
				MR‐Egger	0.377	4.597	1.458	0.000–1.19E + 04	0.940
				Weighted median	0.177	0.122	1.194	0.939–1.517	0.148
ieu‐b‐2	pheno.824	*Oribacterium* ^g^	3	IVW	0.314	0.119	1.369	1.085–1.727	0.008^**^
				MR‐Egger	2.028	2.471	7.597	0.060–963.622	0.563
				Weighted median	0.351	0.148	1.421	1.064–1.898	0.017

Abbreviations: 95% CI, 95% confidence interval; AD, Alzheimer's disease; f, family; g, genus; IVW, inverse‐variance weighted; MR, Mendelian Randomization; o, Order; OR, odds ratio; s, species; SE, Standard error of estimate; SNP(n), numb/ers of single nucleotide polymorphisms.

*p ≤ 0.001; **p ≤ 0.01

### 3.2 Saliva Microbiota

**FIGURE 2:**
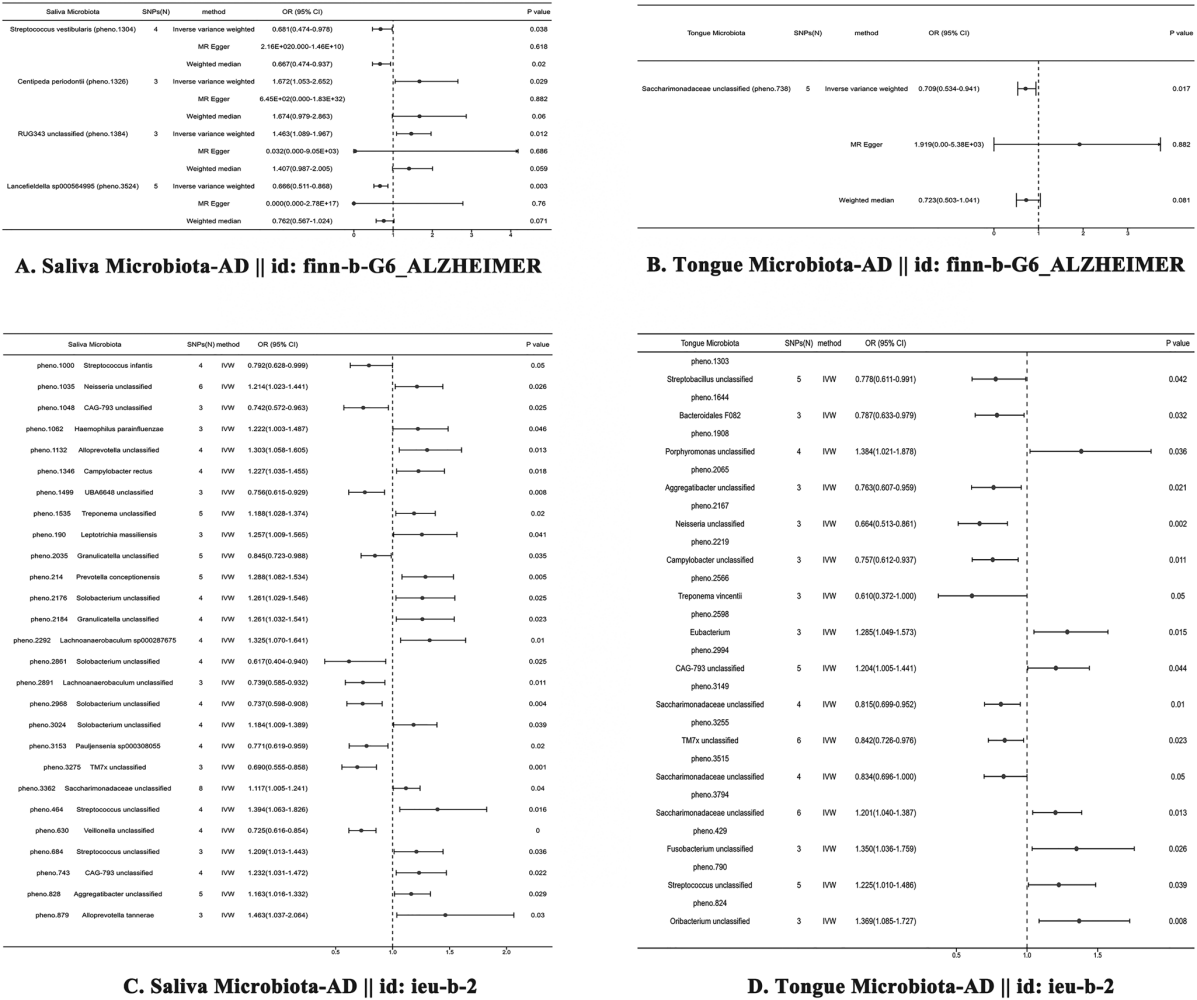
Causal effects of the oral microbiota on Alzheimer's disease. Results from IVW and error bars indicate 95% confidence intervals. AD, Alzheimer's disease; OR, odds ratio; SNPs, Single nucleotide polymorphisms.

Among the salivary microbiota, MR analysis revealed that 31 specific OM taxa (four from the finn‐b‐G6_ALZHEIMER dataset and 27 from the ieu‐b‐2 dataset) showed statistically significant (*p* ≤ 0.05) associations with AD. Of these, 12 showed negative and 19 positive associations, and 11 taxa were identified at the species level, 19 at the genus level, and one at the family level. Notably, *Lancefieldella* sp000564995, UBA6648, *Solobacterium* (pheno.2968), TM7x (pheno.3275), and *Veillonella* demonstrated a protective effect against AD at a higher statistical threshold (OR < 1, *p* ≤ 0.01). Conversely, *Prevotella conceptionensis* and *Lachnoanaerobaculum* sp000287675 showed a significant positive correlation with AD, suggesting their potential role as risk factors in AD development (OR > 1, *p* ≤ 0.01). Remarkably, among these taxa, TM7x (pheno.3275) and *Veillonella* exhibited a strong negative correlation with AD (*p* ≤ 0.001), highlighting their profound value in AD research (Figure 2, Table 2).

### 3.3 Tongue Microbiota

**FIGURE 3:**
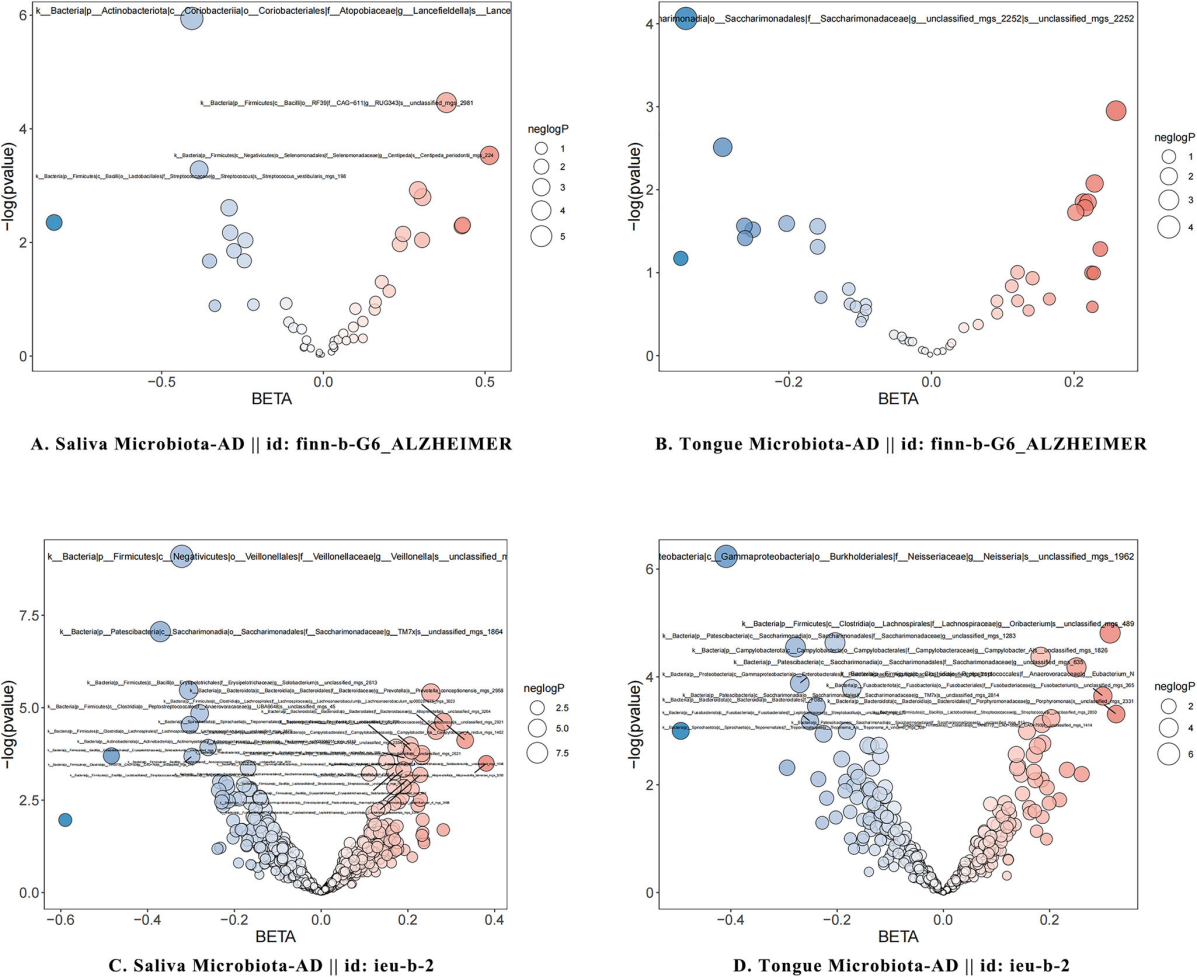
Bubble plot of Oral microbiota associations with Alzheimer's disease Risk. This bubble plot details the associations between oral microbiota and AD risk, highlighting significant effect sizes and levels of statistical significance. Each bubble's size represents the effect size (*β*), while the color indicates the statistical significance (*p* value). Red represents a positive relationship between OM and AD, and blue represents a negative relationship between OM and AD, with darker colors indicating greater strength of association.

As for tongue microbiota, 17 microbial taxa (one from the finn‐b‐G6_ALZHEIMER dataset and 16 from the ieu‐b‐2 dataset) were found to be associated with AD (*p* ≤ 0.05). Among these, only *Treponema vincentii* was identified at the species level, while 11 were at the genus level and five at the family level. *Saccharimonadaceae* (pheno.738), *Streptobacillus*, *Bacteroidales* F082, *Aggregatibacter* (pheno.2065), *Neisseria* (pheno.2167), *Campylobacter* (pheno.2219), *Treponema vincentii*, *Saccharimonadaceae* (pheno.3149), *Saccharimonadaceae* TM7x (pheno.3255) *and Saccharimonadaceae* (pheno.3515) demonstrated negative associations with AD (OR < 1, *p* ≤ 0.05). Conversely, *Saccharimonadaceae* (pheno.3794), *Fusobacterium*, *Streptococcus* (pheno.790), *Oribacterium*, *Porphyromonas*, *Eubacterium* and CAG‐793 (pheno.2994) exhibited positive correlations with AD outcomes (OR > 1, *p* ≤ 0.05). Among these 17 taxa, *Neisseria* (pheno.2167), *Saccharimonadaceae* (pheno.3149), and *Oribacterium* demonstrated a stronger association with AD (*p* ≤ 0.01) (Figure 3, Table 2).

### 3.4 Genes and Functions

**FIGURE 4:**
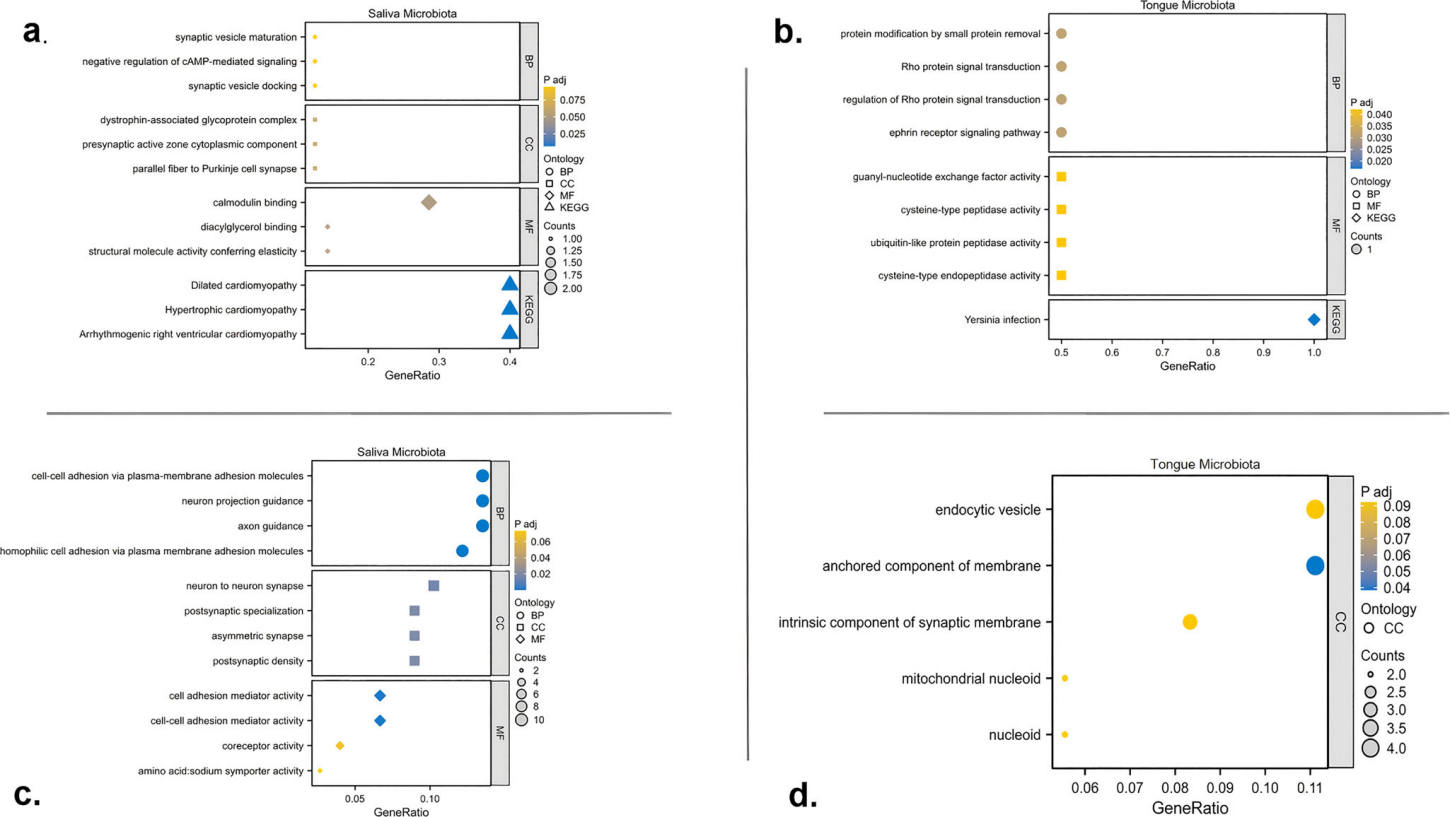
Genetic function analyses for oral microbiota related genes in this study. BP, biological process; CC, cellular component; MF, KEGG, *Kyoto Encyclopedia of Genes and Genomes*; molecular function.

The correspondence between SNPs, genes, and their functions is outlined in Supporting Information S5. GO enrichment analyses revealed that the OM identified in this MR study, which showed significant associations with AD risk, may exert a causal influence through various gene regulatory mechanisms related to neurocognitive function. While KEGG pathway analyses did not identify specific signaling pathways directly associated with AD, they uncovered links to cardiovascular disease, suggesting a broader impact of OM on systemic health (Figure 4).

### 3.5 Heterogeneity and Pleiotropy Analyses

The results of Cochran's *Q* test, shown in Table S1, indicated no significant heterogeneity among the associations (*p* > 0.05), thereby confirming the consistency of the IVs used. Moreover, the MR‐Egger intercept tests, as shown in Table S2, did not indicate significant evidence of horizontal pleiotropy for the observed associations (*p* > 0.05). This suggests that the findings are unlikely to be influenced by pleiotropy, thereby reinforcing the robustness of the results.

## 4 Discussion

Through MR analysis, we investigated the impact of 594 OM on AD. Our results indicated that up to 48 microbiota were statistically significantly associated with AD outcomes (*p* ≤ 0.05). Among these, 12 microbiota were identified at the species level, while the 48 taxa are distributed across eight phyla, including *Firmicutes* (*n* = 22), *Patescibacteria* (*n* = 7), *Proteobacteria* (*n* = 5), *Bacteroidota* (*n* = 5), *Campylobacterota* (*n* = 2), *Spirochaetota* (*n* = 2), *Fusobacteriota* (*n* = 3), and *Actinobacteriota* (*n* = 2). At the species level, *Streptococcus vestibularis*, *Lancefieldella* sp000564995, *Streptococcus infantis*, *Pauljensenia* sp000308055 and *Treponema vincentii* exhibited negative associations with AD (OR < 1, *p* ≤ 0.05), suggesting these five OM may serve as protective factors against AD. Conversely, *Centipeda periodontii*, *Haemophilus parainfluenzae*, *Campylobacter rectus*, *Leptotrichia massiliensis*, *Prevotella conceptionensis*, *Lachnoanaerobaculum* sp000287675, and *Alloprevotella tannerae* were significantly positively associated with AD (OR > 1, *p* ≤ 0.05). These findings suggest that these seven microbial species may act as risk factors, potentially playing a promotive role in the pathophysiology of AD. Notably, the Saccharimonadaceae family (*n* = 7), *Streptococcus* genus (*n* = 5), *Solobacterium* genus (*n* = 4), and CAG‐793 genus (*n* = 3) exhibit a broad microbial spectrum in their associations with AD, suggesting a potentially close relationship between these OM and the pathogenesis of AD, warranting further investigation.

Saccharimonadaceae, a family within the *Saccharibacteria* phylum, was formerly known as TM7 (Bor et al. 2019). Tran et al. (2019) demonstrated lower levels of TM7 in the gut microbiota of APOE4 transgenic mice compared to APOE3 transgenic mice, suggesting a potential inverse association between TM7 and AD. Previous research has shown that TM7 is active in inflammatory environments, with increased prevalence in various oral diseases (Kuehbacher et al. 2008; Acharya et al. 2019). However, recent studies indicate that TM7 may reduce the likelihood of periodontitis and bone resorption in mice (Chipashvili et al. 2021). TM7x, the first cultivated member of the TM7 phylum, is an epibiotic bacterium capable of both killing its host bacteria and forming stable relationships (Dong et al. 2024). J. Tian, Utter, et al. (2022) found that TM7x may indirectly benefit oral health by metabolizing arginine to produce ATP and ammonia, which raises pH. Our study supports the protective effects of TM7x against AD (pheno.3275 OR = 0.690, 95% CI 0.555–0.858, *p* = 0.001; pheno.1767 OR = 0.888, 95% CI 0.792–0.996, *p* = 0.043), aligning with its effects on oral health. The potential mechanism behind these protective effects may involve the *“oral microbiota dysbiosis → inflammation → blood–brain barrier disruption → entry of inflammatory mediators and bacterial metabolites → neuropsychiatric disorders”* pathway (Paudel et al. 2022). The oral health benefits of TM7x and the role of TM7x in maintaining microbial homeostasis may elucidate their protective effects against AD.

*Streptococcus* plays a crucial role in the development and organization of the OM. Studies have shown that *Streptococcus* load in the oral cavity generally increases with age, and individuals with lower *Streptococcus* counts tend to have better oral health compared to those with higher concentrations (Al‐Haboubi et al. 2014; Fure and Zickert 1990; Papapanou et al. 2020). In addition, recent research has revealed a reduction in oral microbial diversity among AD patients, with enrichment of the Streptococcaceae family (Y.‐F. Wu, Lee, et al. 2021; Kamer et al. 2021). A large‐scale population cohort study also demonstrated that individuals with a high burden of periodontal pathogens, particularly *P. gingivalis and Streptococcus oralis*, had a significantly increased risk of developing AD (Beydoun et al. 2024). Although research linking the *Streptococcus* genus to AD remains limited, our MR analysis has uncovered a causal relationship between five *Streptococcus* genus and AD—three positively correlated (pheno.464, pheno.684, pheno.790) and two negatively correlated (pheno.1304, pheno.1000). These findings suggest a promising avenue for further exploration of the *Streptococcus*‐AD connection.

*Solobacterium* genus, a known component of the salivary microbiome, has not yet been investigated for its potential role in AD. Our MR analysis provides novel insights into *Solobacterium*, suggesting an association with AD (pheno.2176, pheno.2861, pheno.2968, pheno.3024) beyond its established links to oral health (Wuri et al. 2023; Vancauwenberghe et al. 2013). As for the CAG‐793 genus, although limited research exists, its classification within the *Firmicutes* phylum is notable. *Firmicutes*, comprising 22 of the 48 taxa in our analysis, appear to corroborate previous findings indicating a significant association with AD (Jungbauer et al. 2022). Previous studies have shown that individuals with AD exhibit higher oral *Firmicutes* abundance compared to controls (Y.‐F. Wu, Lee, et al. 2021; Kamer et al. 2021). However, an animal study reported decreased amyloid‐β deposition and enhanced cognitive function, which correlated with an increase in *Firmicutes* abundance in the gut microbiome (Sun et al. 2020). These findings suggest a significant yet inconsistent role of the *Firmicutes* phylum in AD pathogenesis.

*Veillonella* genus exhibited a strong association with AD outcomes (*p* < 0.001), highlighting its significant potential for further investigation. While some studies suggest that *Veillonella* may help prevent dental caries by converting lactate into weaker acids and producing NO_2_ from NO_3_ (Giacomini et al. 2023; Wicaksono et al. 2020), the predominant view is that it plays a key role in the progression of dental caries and periodontal disease, either directly or through synergistic interactions with *Streptococcus* and *P. gingivalis* (Luo et al. 2020). Given these insights, the potential protective effect of *Veillonella* against AD observed in our study (OR = 0.725, 95% CI 0.616–0.854, *p* < 0.001) introduces a novel perspective on *Veillonella* as a potentially beneficial factor for human health.

The relationship between periodontal microbiota dysbiosis and AD has been a key area of research, with many AD‐related OM species identified in previous studies also appearing in this MR analysis. The risk of periodontal disease increases with age, mirroring the age‐related nature of neurodegenerative diseases like AD (Demmer et al. 2012). Furthermore, the spread of oral bacteria and systemic elevation of inflammatory cytokines may heighten the risk of chronic diseases such as AD (Beydoun et al. 2024; Slade et al. 2003). The primary organisms linked with severe periodontal lesions include *P. gingivalis*, *Prevotella intermedia*, *Bacteroides forsythus*, *Actinobacillus actinomycetemcomitans*, and *Treponema denticola* (Zambon 1996). *Porphyromonas gingivalis*, a major pathogen in severe periodontitis, has been a central focus of the “*OM‐AD*” field (Jungbauer et al. 2022; Papapanou et al. 2020). Our study supports the role of *P. gingivalis* as a potential causative factor in AD (OR = 1.384, 95% CI 1.021–1.878, *p* = 0.036). Previous human studies have also found a high prevalence of *P. gingivalis* and its virulence factors in brain samples from AD patients (Dominy et al. 2019). However, findings in this field are inconsistent. Some studies report that *P. gingivalis* levels in AD patients were like or lower than in controls, while others failed to detect *P. gingivalis* altogether (Lobmaier et al. 2009; Riviere et al. 2002; Emery et al. 2017; Franciotti et al. 2021; Sparks Stein et al. 2012). The reasons for these concerns are multifaceted. For example, sample quality issues and the lack of baseline data on oral hygiene and dental health in the study also reduced the reliability of the findings. In addition, the sensitivity and specificity of the methods used to interpret the data deserve scrutiny, which may exacerbate the significant controversy and uncertainty prevalent in this area of research.

Furthermore, our analysis identified a significant positive association between *Treponema*, another bacterium closely linked to the severity of periodontitis (Papapanou et al. 2020), and AD (pheno.1535, OR = 1.188, 95% CI 1.028–1.374, *p* = 0.020), corroborating previous findings (Riviere et al. 2002; Su et al. 2021). Similarly, *Prevotella* showed a positive correlation with AD (OR = 1.288, 95% CI 1.082–1.534, *p* = 0.005), consistent with earlier clinical data from both oral and gut microbiota studies (Guo et al. 2021; Beydoun et al. 2021).

We did not find direct evidence linking *Actinobacillus actinomycetemcomitans* with AD. However, closely related genera within the Pasteurellaceae family, such as *Haemophilus* (pheno.1062) and *Aggregatibacter* (pheno.828, pheno.2065), showed significant associations with AD outcomes in this study, despite the differing directions of these associations. These findings prompt further exploration of the roles these bacterial groups may play in AD pathogenesis, particularly *Aggregatibacter*, which has been implicated in disrupting the BBB and triggering CNS inflammation in preclinical studies (Díaz‐Zúñiga et al. 2019; Han et al. 2019). This mechanism may underlie its potential link to AD.

*Campylobacter* is a predominant cause of bacterial gastroenteritis globally (Igwaran and Okoh 2019), yet its potential association with AD has not been adequately investigated. Our findings suggest oral *Campylobacter* (pheno.2219, OR = 0.757, 95% CI 0.612–0.937, *p* = 0.011) may be protective against AD, while *Campylobacter rectus* (OR = 1.277, 95% CI 1.035–1.455, *p* = 0.018) could increase AD risk. *Pauljensenia*, a member of the Actinomycetaceae family, is a Gram‐positive bacterium (Nouioui et al. 2018). Our analysis revealed significant negative associations between *Pauljensenia* sp000308055 and AD outcome (OR = 0.771, 95% CI 0.619–0.959, *p* = 0.020). However, previous studies have suggested that oral Actinomycetaceae abundance is increased in AD patients, which contradicts our findings (Y.‐F. Wu, Lee, et al. 2021; Kamer et al. 2021). Extensive research on *Fusobacterium* shows mixed results–some studies report a decrease in AD patients (Kamer et al. 2021), while in vitro studies link it to amyloid adhesion, promoting AD‐like pathology (Meng et al. 2021). Our MR analysis is consistent with the latter (OR = 1.350, 95% CI 1.036–1.759, *p* = 0.026).

Yussof et al. (2020) indicated that several genera, including *Eubacterium*, *Bacteroides*, *Porphyromonas*, and *Fusobacteria*, are linked to AD. These findings align with our results (pheno.2598, pheno.1644, pheno.1908, pheno.429). Furthermore, their analysis revealed that *Porphyromonas* and *Neisseria* are common genera associated with both AD and alcohol abuse, which also corroborates our conclusions (pheno.1908, OR = 1.384, 95% CI 1.021–1.878, *p* = 0.036; pheno.1035, OR = 1.214, 95% CI 1.023–1.441, *p* = 0.026). In addition, our study reports novel associations between AD and the genera *Granulicatella*, *Alloprevotella*, *Lachnoanaerobaculum*, *Centipeda*, *Lancefieldella*, *Leptotrichia*, RUG343, UBA6648, and *Oribacterium*, which have not been previously documented, expanding our understanding of OM's potential link to AD.

While our findings provide robust genetic evidence supporting the causal role of specific OM in AD, it is essential to contextualize these results within the broader microbiome–brain axis framework. A recent MR study by Zhao et al. (2024) investigated the gut microbiota and similarly identified both protective (e.g., *Clostridia*, *Bifidobacterium bifidum*) and risk‐enhancing taxa (e.g., *Actinobacteria*, *Betaproteobacteria*) in relation to AD susceptibility. Notably, certain genera such as *Veillonella* exhibited opposing associations depending on anatomical location—being positively associated with AD risk in the gut, yet demonstrating a protective effect in our OM analysis. These contrasting patterns underscore the importance of microbial niche specificity and suggest that microbial influence on neurodegenerative processes may be modulated by distinct local host–microbiota interactions. Integrating multi‐site microbiome data may therefore offer a more comprehensive perspective on the etiological pathways linking dysbiosis to AD.

Genetic analyses were performed to clarify the biological functions of these OM‐related genes in the context of AD. Our analysis revealed that OM‐related genes are involved in neurophysiological functions, particularly in *synaptic activity*, *neuronal signaling*, and *cell adhesion*. However, the genes associated with saliva and tongue microbiota do not completely overlap in their functional roles. Commonalities include the involvement of both saliva and tongue microbiota in cellular adhesion and synaptic processes, suggesting a shared role in neural connectivity and communication. The enrichment of signaling pathways further underscores the potential influence of OM on broader physiological functions. In terms of differences, saliva microbiota is more focused on synaptic vesicle function and neuronal structures, indicating a direct impact on neural communication. In contrast, tongue microbiota is associated with protein modification and Rho signaling pathways, reflecting a broader role in cellular dynamics and signaling rather than direct synaptic interactions. This distinction highlights the diverse roles of OM in neurological and cellular processes, with significant implications for health and disease. In summary, our functional enrichment analysis at the gene level offers both supportive evidence and new insights for investigating the potential causal relationship between OM and AD.

This study has several limitations. First, the participants were predominantly of European descent, limiting the generalizability of our findings. Incorporating a third dataset from a geographically distinct population, such as a Chinese cohort, could provide valuable insights into the variations in OM across different populations and strengthen the generalizability of these findings in the future. Second, The OM data in our study were derived from a Chinese population, whereas the AD GWAS datasets originated predominantly from individuals of European descent. Geographic and ethnic differences—particularly in diet, lifestyle, oral hygiene practices, and environmental exposures—can influence the composition and diversity of the OM. Therefore, potential population stratification is an important consideration when interpreting our results. Nonetheless, previous studies have applied cross‐ethnic MR frameworks using OM GWAS data (Z. Li et al. 2025; Feng et al. 2023), indicating that well‐associated genetic instruments may remain valid across populations. In our study, stringent IV selection and sensitivity analyses were employed to minimize bias from population heterogeneity. Furthermore, the consistent associations observed across two independent AD datasets reinforce the robustness of our findings. Future studies with harmonized multi‐ethnic data are still needed to confirm and extend these results. Third, demographic variations between data sources, such as gender, age, and education—factors potentially related to AD—may introduce confounders. Although MR analysis attempts to account for these, the absence of such data precludes further statistical adjustments. This issue is common to all two‐sample MR studies. Fourth, the limited number of valid SNPs in this MR analysis prevented the use of the MR‐PRESSO test, requiring the more conservative MR‐Egger method to detect horizontal pleiotropy. We believe that the scarcity of SNPs is due to both the dataset characteristics and strict SNP selection criteria. Relaxing these criteria to increase SNP numbers would compromise the results’ interpretability and reliability, especially given the substantial positive findings already obtained. Lastly, although multiple testing corrections were performed, several associations did not remain significant after adjustment. Given the exploratory nature of this study, we prioritized uncovering potential signals that may warrant further investigation, rather than applying strict correction thresholds that might obscure biologically relevant associations. Despite these limitations, this study has the potential to significantly advance AD research. We explored potential OM–AD associations in different genetic backgrounds by combining different databases. Furthermore, understanding the role of OM in AD pathogenesis may bring new perspectives for thinking about the development of diagnostic and therapeutic programs for AD.

## 5 Conclusion

This study examined the causal relationships between OM from saliva and tongue dorsum samples and AD using European and FinnGen datasets. MR analysis identified 48 OM species potentially linked to AD, suggesting OM's role in disease pathogenesis. Combined with gene‐level functional analyses, this research lays the groundwork for future microbial‐targeted therapies and preventive strategies, such as dietary interventions, probiotics, and microbiota transplantation, as well as for identifying early diagnostic markers for AD.

## Author Contributions

**Zhichao Li**: conceptualization, methodology, software, data curation, investigation, formal analysis, writing – original draft, writing – review and editing. **Yihan Kang**: methodology, software, data curation, formal analysis, investigation, visualization, writing – review and editing. **Shuai Li**: conceptualization, methodology, writing – original draft, software, writing – review and editing. **Shiqi Guo**: methodology, data curation, software, visualization. **Hui Zheng**: conceptualization, investigation, validation, supervision, project administration, resources, writing – review and editing.

## Conflicts of Interest

The authors declare no conflicts of interest.

## Peer Review

The peer review history for this article is available at https://publons.com/publon/10.1002/brb3.70753

## Ethic Statement

This study only used publicly available data, and the relevant ethical approval can be found in the corresponding studies referenced in the Section 2.

## Supporting information




Supplementary Information

Supplementary Information

Supplementary Information

Supplementary Information

Supplemnetary 5

Table S1‐S2

## Data Availability

Additional data supporting the findings of this study are available from the corresponding author upon reasonable request.
