# Supplementary material for: Unveiling the Relationship Between Oral Microbiota and Alzheimer's Disease: A Genetic Instrumental Variable Analysis via Mendelian Randomization
Source: Brain Behav. 2025 Aug 4;15(8):e70753. doi: 10.1002/brb3.70753 (PMC12321961; doi:10.1002/brb3.70753)
Supplement: Supplementary file 2 — Supplementary Information [file BRB3-15-e70753-s003.zip › Table S2_Pleiotropy analysis.docx]

**Table S5. The pleiotropy test of the MR analysis.**

| **Outcome**  **(ID)** | **Outcome** | **Exposure**  **(ID)** | **Exposure**  **(microbiota)** | **Egger intercept** | **SE** | **p-val** |
| --- | --- | --- | --- | --- | --- | --- |
| **Saliva Microbiota** | | | | | | |
| **finn-b-G6_ALZHEIMER** | **AD** | pheno.1304 | Streptococcus  vestibularis**^s^** | -0.665 | 1.062 | 0.595 |
|  |  |  |  |  |  |  |
|  |  | pheno.1326 | Centipeda periodontii**^s^** | -0.690 | 4.008 | 0.891 |
|  |  |  |  |  |  |  |
|  |  | pheno.1384 | RUG343**^g^** | 0.451 | 0.754 | 0.657 |
|  |  |  |  |  |  |  |
|  |  | pheno.3524 | Lancefieldella  sp000564995**^s^** | 0.920 | 2.887 | 0.771 |
|  |  |  |  |  |  |  |
| **ieu-b-2** | **AD** | pheno.1000 | Streptococcus infantis**^s^** | -0.204 | 0.272 | 0.531 |
|  |  |  |  |  |  |  |
|  |  | pheno.1035 | Neisseria**^g^** | -0.194 | 0.273 | 0.516 |
|  |  |  |  |  |  |  |
|  |  | pheno.1048 | CAG-793**^g^** | 0.078 | 0.645 | 0.923 |
|  |  |  |  |  |  |  |
|  |  | pheno.1062 | Haemophilus parainfluenzae**^s^** | -0.077 | 0.270 | 0.823 |
|  |  |  |  |  |  |  |
|  |  | pheno.1132 | Alloprevotella**^g^** | 0.735 | 0.707 | 0.408 |
|  |  |  |  |  |  |  |
|  |  | pheno.1346 | Campylobacter rectus**^s^** | 0.353 | 0.453 | 0.517 |
|  |  |  |  |  |  |  |
|  |  | pheno.1499 | UBA6648**^g^** | 0.028 | 0.312 | 0.942 |
|  |  |  |  |  |  |  |
|  |  | pheno.1535 | Treponema**^g^** | -0.136 | 0.113 | 0.315 |
|  |  |  |  |  |  |  |
|  |  | pheno.190 | Leptotrichia  massiliensis**^s^** | 1.411 | 1.125 | 0.428 |
|  |  |  |  |  |  |  |
|  |  | pheno.2035 | Granulicatella**^g^** | -0.084 | 0.197 | 0.697 |
|  |  |  |  |  |  |  |
|  |  | pheno.214 | Prevotella conceptionensis**^s^** | -0.469 | 0.461 | 0.384 |
|  |  |  |  |  |  |  |
|  |  | pheno.2176 | Solobacterium**^g^** | -1.732 | 1.339 | 0.325 |
|  |  |  |  |  |  |  |
|  |  | pheno.2184 | Granulicatella**^g^** | 0.145 | 0.191 | 0.526 |
|  |  |  |  |  |  |  |
|  |  | pheno.2292 | Lachnoanaerobaculum sp000287675**^s^** | -1.201 | 1.560 | 0.522 |
|  |  |  |  |  |  |  |
|  |  | pheno.2861 | Solobacterium**^g^** | 2.347 | 0.665 | 0.072 |
|  |  |  |  |  |  |  |
|  |  | pheno.2891 | Lachnoanaerobaculum**^g^** | 0.410 | 0.535 | 0.584 |
|  |  |  |  |  |  |  |
|  |  | pheno.2968 | Solobacterium**^g^** | 0.143 | 0.565 | 0.824 |
|  |  |  |  |  |  |  |
|  |  | pheno.3024 | Solobacterium**^g^** | 0.653 | 0.371 | 0.221 |
|  |  |  |  |  |  |  |
|  |  | pheno.3153 | Pauljensenia sp000308055**^s^** | -0.594 | 0.559 | 0.399 |
|  |  |  |  |  |  |  |
|  |  | pheno.3275 | Saccharimonadaceae TM7x**^g^** | -0.299 | 0.539 | 0.677 |
|  |  |  |  |  |  |  |
|  |  | pheno.3362 | Saccharimonadaceae**^f^** | -0.141 | 0.159 | 0.408 |
|  |  |  |  |  |  |  |
|  |  | pheno.464 | Streptococcus**^g^** | 0.248 | 0.727 | 0.766 |
|  |  |  |  |  |  |  |
|  |  | pheno.630 | Veillonella**^g^** | 0.690 | 0.889 | 0.519 |
|  |  |  |  |  |  |  |
|  |  | pheno.684 | Streptococcus**^g^** | 1.068 | 1.461 | 0.598 |
|  |  |  |  |  |  |  |
|  |  | pheno.743 | CAG-793**^g^** | 0.166 | 0.334 | 0.669 |
|  |  |  |  |  |  |  |
|  |  | pheno.828 | Aggregatibacter**^g^** | -0.191 | 0.165 | 0.331 |
|  |  |  |  |  |  |  |
|  |  | pheno.879 | Alloprevotella tannerae**^g^** | 1.019 | 0.723 | 0.393 |
|  |  |  |  |  |  |  |
| **Tongue Microbiota** | | | | | | |
| **finn-b-G6_ALZHEIMER** | **AD** | pheno.738 | Saccharimonadaceae**^f^** | -0.040 | 0.276 | 0.892 |
|  |  |  |  |  |  |  |
| **ieu-b-2** | **AD** | pheno.1303 | Streptobacillus**^g^** | 0.234 | 0.368 | 0.570 |
|  |  |  |  |  |  |  |
|  |  | pheno.1644 | Bacteroidales  F082**^f^** | -0.249 | 0.290 | 0.548 |
|  |  |  |  |  |  |  |
|  |  | pheno.1908 | Porphyromonas**^g^** | 1.829 | 1.337 | 0.305 |
|  |  |  |  |  |  |  |
|  |  | pheno.2065 | Aggregatibacter**^g^** | -0.344 | 0.838 | 0.752 |
|  |  |  |  |  |  |  |
|  |  | pheno.2167 | Neisseria**^g^** | -0.048 | 1.307 | 0.977 |
|  |  |  |  |  |  |  |
|  |  | pheno.2219 | Campylobacter**^g^** | 0.123 | 0.349 | 0.785 |
|  |  |  |  |  |  |  |
|  |  | pheno.2566 | Treponema vincentii**^s^** | -1.443 | 1.393 | 0.489 |
|  |  |  |  |  |  |  |
|  |  | pheno.2598 | Eubacterium**^g^** | 0.006 | 0.462 | 0.991 |
|  |  |  |  |  |  |  |
|  |  | pheno.2994 | CAG-793**^g^** | 0.577 | 0.844 | 0.543 |
|  |  |  |  |  |  |  |
|  |  | pheno.3149 | Saccharimonadaceae**^f^** | -0.172 | 0.337 | 0.661 |
|  |  |  |  |  |  |  |
|  |  | pheno.3255 | Saccharimonadaceae TM7x**^g^** | 0.119 | 0.154 | 0.483 |
|  |  |  |  |  |  |  |
|  |  | pheno.3515 | Saccharimonadaceae**^f^** | -0.054 | 0.133 | 0.723 |
|  |  |  |  |  |  |  |
|  |  | pheno.3794 | Saccharimonadaceae**^f^** | 0.121 | 0.238 | 0.637 |
|  |  |  |  |  |  |  |
|  |  | pheno.429 | Fusobacterium**^g^** | -0.592 | 0.327 | 0.321 |
|  |  |  |  |  |  |  |
|  |  | pheno.790 | Streptococcus**^g^** | -0.018 | 0.479 | 0.972 |
|  |  |  |  |  |  |  |
|  |  | pheno.824 | Oribacterium**^g^** | -0.179 | 0.258 | 0.614 |
|  |  |  |  |  |  |  |
|  |  |  |  |  |  |  |
|  |  |  |  |  |  |  |
| **MR,** Mendelian Randomization; **AD**, Alzheimer's disease; **SE**, Standard error of estimate | | | | | | |
