# Supplementary material for: Unveiling the Relationship Between Oral Microbiota and Alzheimer's Disease: A Genetic Instrumental Variable Analysis via Mendelian Randomization
Source: Brain Behav. 2025 Aug 4;15(8):e70753. doi: 10.1002/brb3.70753 (PMC12321961; doi:10.1002/brb3.70753)
Supplement: Supplementary file 2 — Supplementary Information [file BRB3-15-e70753-s003.zip › Table S1_Heterogeneity test.docx]

**Table S4. The heterogeneity test of the MR analysis.**

| **Outcome**  **(ID)** | **Outcome** | **Exposure**  **(ID)** | **Exposure**  **(microbiota)** | **Method** | **Cochran's Q** | **Q-df** | **Q-pval** |
| --- | --- | --- | --- | --- | --- | --- | --- |
| **Saliva Microbiota** | | | | | | | |
| **finn-b-G6_ALZHEIMER** | **AD** | pheno.1304 | Streptococcus  vestibularis**^s^** | IVW | 5.248 | 3 | 0.155 |
|  |  |  |  | MR-Egger | 4.388 | 2 | 0.111 |
|  |  | pheno.1326 | Centipeda periodontii**^s^** | IVW | 0.030 | 2 | 0.985 |
|  |  |  |  | MR-Egger | 0.000 | 1 | 0.990 |
|  |  | pheno.1384 | RUG343**^g^** | IVW | 0.675 | 2 | 0.714 |
|  |  |  |  | MR-Egger | 0.317 | 1 | 0.573 |
|  |  | pheno.3524 | Lancefieldella  sp000564995**^s^** | IVW | 5.668 | 4 | 0.225 |
|  |  |  |  | MR-Egger | 5.482 | 3 | 0.140 |
| **ieu-b-2** | **AD** | pheno.1000 | Streptococcus infantis**^s^** | IVW | 5.169 | 3 | 0.160 |
|  |  |  |  | MR-Egger | 4.034 | 2 | 0.133 |
|  |  | pheno.1035 | Neisseria**^g^** | IVW | 1.573 | 5 | 0.904 |
|  |  |  |  | MR-Egger | 1.067 | 4 | 0.899 |
|  |  | pheno.1048 | CAG-793**^g^** | IVW | 0.248 | 2 | 0.883 |
|  |  |  |  | MR-Egger | 0.233 | 1 | 0.629 |
|  |  | pheno.1062 | Haemophilus parainfluenzae**^s^** | IVW | 0.552 | 2 | 0.759 |
|  |  |  |  | MR-Egger | 0.470 | 1 | 0.493 |
|  |  | pheno.1132 | Alloprevotella**^g^** | IVW | 2.962 | 3 | 0.398 |
|  |  |  |  | MR-Egger | 1.881 | 2 | 0.390 |
|  |  | pheno.1346 | Campylobacter rectus**^s^** | IVW | 1.978 | 3 | 0.577 |
|  |  |  |  | MR-Egger | 1.370 | 2 | 0.504 |
|  |  | pheno.1499 | UBA6648**^g^** | IVW | 0.284 | 2 | 0.867 |
|  |  |  |  | MR-Egger | 0.276 | 1 | 0.599 |
|  |  | pheno.1535 | Treponema**^g^** | IVW | 3.340 | 4 | 0.503 |
|  |  |  |  | MR-Egger | 1.889 | 3 | 0.596 |
|  |  | pheno.190 | Leptotrichia  massiliensis**^s^** | IVW | 1.887 | 2 | 0.389 |
|  |  |  |  | MR-Egger | 0.313 | 1 | 0.576 |
|  |  | pheno.2035 | Granulicatella**^g^** | IVW | 2.895 | 4 | 0.576 |
|  |  |  |  | MR-Egger | 2.711 | 3 | 0.438 |
|  |  | pheno.214 | Prevotella conceptionensis**^s^** | IVW | 2.553 | 4 | 0.635 |
|  |  |  |  | MR-Egger | 1.519 | 3 | 0.678 |
|  |  | pheno.2176 | Solobacterium**^g^** | IVW | 4.639 | 3 | 0.200 |
|  |  |  |  | MR-Egger | 2.527 | 2 | 0.283 |
|  |  | pheno.2184 | Granulicatella**^g^** | IVW | 0.668 | 3 | 0.881 |
|  |  |  |  | MR-Egger | 0.090 | 2 | 0.956 |
|  |  | pheno.2292 | Lachnoanaerobaculum sp000287675**^s^** | IVW | 1.388 | 3 | 0.708 |
|  |  |  |  | MR-Egger | 0.795 | 2 | 0.672 |
|  |  | pheno.2861 | Solobacterium**^g^** | IVW | 13.181 | 3 | 0.004 |
|  |  |  |  | MR-Egger | 0.716 | 2 | 0.699 |
|  |  | pheno.2891 | Lachnoanaerobaculum**^g^** | IVW | 0.761 | 2 | 0.684 |
|  |  |  |  | MR-Egger | 0.175 | 1 | 0.676 |
|  |  | pheno.2968 | Solobacterium**^g^** | IVW | 0.516 | 3 | 0.915 |
|  |  |  |  | MR-Egger | 0.452 | 2 | 0.798 |
|  |  | pheno.3024 | Solobacterium**^g^** | IVW | 3.163 | 3 | 0.367 |
|  |  |  |  | MR-Egger | 0.074 | 2 | 0.964 |
|  |  | pheno.3153 | Pauljensenia sp000308055**^s^** | IVW | 4.077 | 3 | 0.253 |
|  |  |  |  | MR-Egger | 2.604 | 2 | 0.272 |
|  |  | pheno.3275 | Saccharimonadaceae TM7x**^g^** | IVW | 1.683 | 2 | 0.431 |
|  |  |  |  | MR-Egger | 1.286 | 1 | 0.257 |
|  |  | pheno.3362 | Saccharimonadaceae**^f^** | IVW | 5.917 | 7 | 0.549 |
|  |  |  |  | MR-Egger | 5.125 | 6 | 0.528 |
|  |  | pheno.464 | Streptococcus**^g^** | IVW | 3.833 | 3 | 0.280 |
|  |  |  |  | MR-Egger | 3.623 | 2 | 0.163 |
|  |  | pheno.630 | Veillonella**^g^** | IVW | 1.045 | 3 | 0.790 |
|  |  |  |  | MR-Egger | 0.442 | 2 | 0.802 |
|  |  | pheno.684 | Streptococcus**^g^** | IVW | 1.376 | 2 | 0.503 |
|  |  |  |  | MR-Egger | 0.841 | 1 | 0.359 |
|  |  | pheno.743 | CAG-793**^g^** | IVW | 2.570 | 3 | 0.463 |
|  |  |  |  | MR-Egger | 2.289 | 2 | 0.318 |
|  |  | pheno.828 | Aggregatibacter**^g^** | IVW | 1.513 | 4 | 0.824 |
|  |  |  |  | MR-Egger | 0.174 | 3 | 0.982 |
|  |  | pheno.879 | Alloprevotella tannerae**^g^** | IVW | 2.315 | 2 | 0.314 |
|  |  |  |  | MR-Egger | 0.329 | 1 | 0.566 |
| **Tongue Microbiota** | | | | | | | |
| **finn-b-G6_ALZHEIMER** | **AD** | pheno.738 | Saccharimonadaceae**^f^** | IVW | 1.398 | 4 | 0.845 |
|  |  |  |  | MR-Egger | 1.337 | 3 | 0.720 |
| **ieu-b-2** | **AD** | pheno.1303 | Streptobacillus**^g^** | IVW | 2.601 | 4 | 0.627 |
|  |  |  |  | MR-Egger | 2.196 | 3 | 0.533 |
|  |  | pheno.1644 | Bacteroidales  F082**^f^** | IVW | 0.787 | 2 | 0.675 |
|  |  |  |  | MR-Egger | 0.047 | 1 | 0.828 |
|  |  | pheno.1908 | Porphyromonas**^g^** | IVW | 4.857 | 3 | 0.183 |
|  |  |  |  | MR-Egger | 2.508 | 2 | 0.285 |
|  |  | pheno.2065 | Aggregatibacter**^g^** | IVW | 1.436 | 2 | 0.488 |
|  |  |  |  | MR-Egger | 1.229 | 1 | 0.268 |
|  |  | pheno.2167 | Neisseria**^g^** | IVW | 1.322 | 2 | 0.516 |
|  |  |  |  | MR-Egger | 1.320 | 1 | 0.251 |
|  |  | pheno.2219 | Campylobacter**^g^** | IVW | 1.093 | 2 | 0.579 |
|  |  |  |  | MR-Egger | 0.970 | 1 | 0.325 |
|  |  | pheno.2566 | Treponema vincentii**^s^** | IVW | 1.399 | 2 | 0.497 |
|  |  |  |  | MR-Egger | 0.326 | 1 | 0.568 |
|  |  | pheno.2598 | Eubacterium**^g^** | IVW | 0.015 | 2 | 0.993 |
|  |  |  |  | MR-Egger | 0.015 | 1 | 0.903 |
|  |  | pheno.2994 | CAG-793**^g^** | IVW | 2.405 | 4 | 0.662 |
|  |  |  |  | MR-Egger | 1.937 | 3 | 0.586 |
|  |  | pheno.3149 | Saccharimonadaceae**^f^** | IVW | 3.652 | 3 | 0.302 |
|  |  |  |  | MR-Egger | 3.231 | 2 | 0.199 |
|  |  | pheno.3255 | Saccharimonadaceae TM7x**^g^** | IVW | 4.263 | 5 | 0.512 |
|  |  |  |  | MR-Egger | 3.667 | 4 | 0.453 |
|  |  | pheno.3515 | Saccharimonadaceae**^f^** | IVW | 1.900 | 3 | 0.593 |
|  |  |  |  | MR-Egger | 1.733 | 2 | 0.420 |
|  |  | pheno.3794 | Saccharimonadaceae**^f^** | IVW | 1.815 | 5 | 0.874 |
|  |  |  |  | MR-Egger | 1.556 | 4 | 0.817 |
|  |  | pheno.429 | Fusobacterium**^g^** | IVW | 3.510 | 2 | 0.173 |
|  |  |  |  | MR-Egger | 0.234 | 1 | 0.629 |
|  |  | pheno.790 | Streptococcus**^g^** | IVW | 1.493 | 4 | 0.828 |
|  |  |  |  | MR-Egger | 1.492 | 3 | 0.684 |
|  |  | pheno.824 | Oribacterium**^g^** | IVW | 0.546 | 2 | 0.761 |
|  |  |  |  | MR-Egger | 0.064 | 1 | 0.800 |
| **MR,** Mendelian Randomization; **AD**, Alzheimer's disease; **IVW**, inverse-variance weighted; **df**, degree of freedom | | | | | | | |
