# Supplementary material for: Unveiling the Relationship Between Oral Microbiota and Alzheimer's Disease: A Genetic Instrumental Variable Analysis via Mendelian Randomization
Source: Brain Behav. 2025 Aug 4;15(8):e70753. doi: 10.1002/brb3.70753 (PMC12321961; doi:10.1002/brb3.70753)
Supplement: Supplementary file 3 — Supplementary Information [file BRB3-15-e70753-s004.pdf]

method	nsnp	b	se	pval
MR Egger	6	-0.942	2.212	0.692
Weighted median	6	0.184	0.095	0.053
Inverse variance weighted	6	0.183	0.073	0.013
Simple mode	6	0.106	0.143	0.492
Weighted mode	6	0.088	0.130	0.528

method	nsnp	b	se	pval
MR Egger	4	0.306	1.199	0.822
Weighted median	4	-0.118	0.122	0.332
Inverse variance weighted	4	-0.182	0.093	0.050
Simple mode	4	-0.106	0.159	0.553
Weighted mode	4	-0.106	0.137	0.495

method	nsnp	b	se	pval
MR Egger	6	-1.248	1.396	0.422
Weighted median	6	-0.102	0.099	0.303
Inverse variance weighted	6	-0.172	0.076	0.023
Simple mode	6	-0.035	0.154	0.831
Weighted mode	6	-0.042	0.149	0.791

method	nsnp	b	se	pval
MR Egger	4	1.404	3.153	0.700
Weighted median	4	-0.201	0.096	0.035
Inverse variance weighted	4	-0.204	0.079	0.010
Simple mode	4	-0.282	0.151	0.158
Weighted mode	4	-0.267	0.143	0.159

method	nsnp	b	se	pval
MR Egger	5	-5.437	8.217	0.555
Weighted median	5	0.168	0.119	0.159
Inverse variance weighted	5	0.185	0.092	0.044
Simple mode	5	0.156	0.128	0.289
Weighted mode	5	0.173	0.118	0.217

method	nsnp	b	se	pval
MR Egger	3	0.189	4.478	0.973
Weighted median	3	0.252	0.127	0.046
Inverse variance weighted	3	0.251	0.103	0.015
Simple mode	3	0.257	0.137	0.203
Weighted mode	3	0.255	0.146	0.222

method	nsnp	b	se	pval
MR Egger	3	13.455	13.470	0.500
Weighted median	3	-0.476	0.313	0.128
Inverse variance weighted	3	-0.494	0.252	0.050
Simple mode	3	-0.188	0.379	0.670
Weighted mode	3	-0.584	0.358	0.244

method	nsnp	b	se	pval
MR Egger	3	-1.455	3.352	0.739
Weighted median	3	-0.342	0.141	0.016
Inverse variance weighted	3	-0.278	0.109	0.011
Simple mode	3	-0.355	0.175	0.180
Weighted mode	3	-0.356	0.164	0.163

method	nsnp	b	se	pval
MR Egger	3	0.058	12.733	0.997
Weighted median	3	-0.390	0.165	0.018
Inverse variance weighted	3	-0.409	0.132	0.002
Simple mode	3	-0.480	0.283	0.232
Weighted mode	3	-0.373	0.161	0.147

method	nsnp	b	se	pval
MR Egger	3	2.962	7.883	0.771
Weighted median	3	-0.246	0.132	0.062
Inverse variance weighted	3	-0.270	0.117	0.021
Simple mode	3	-0.302	0.189	0.251
Weighted mode	3	-0.210	0.145	0.284

method	nsnp	b	se	pval
MR Egger	4	-17.186	12.794	0.311
Weighted median	4	0.396	0.153	0.009
Inverse variance weighted	4	0.325	0.155	0.036
Simple mode	4	0.312	0.219	0.250
Weighted mode	4	0.402	0.155	0.081

method	nsnp	b	se	pval
MR Egger	3	2.142	2.771	0.581
Weighted median	3	-0.257	0.137	0.061
Inverse variance weighted	3	-0.239	0.111	0.032
Simple mode	3	-0.266	0.159	0.235
Weighted mode	3	-0.271	0.152	0.217

method	nsnp	b	se	pval
MR Egger	5	-2.472	3.490	0.530
Weighted median	5	-0.197	0.166	0.236
Inverse variance weighted	5	-0.251	0.123	0.042
Simple mode	5	-0.175	0.205	0.442
Weighted mode	5	-0.208	0.171	0.293

method	nsnp	b	se	pval
MR Egger	3	2.028	2.471	0.563
Weighted median	3	0.351	0.142	0.013
Inverse variance weighted	3	0.314	0.119	0.008
Simple mode	3	0.385	0.186	0.174
Weighted mode	3	0.392	0.172	0.150

method	nsnp	b	se	pval
MR Egger	5	0.377	4.597	0.940
Weighted median	5	0.177	0.126	0.159
Inverse variance weighted	5	0.203	0.099	0.039
Simple mode	5	0.171	0.158	0.340
Weighted mode	5	0.173	0.114	0.203

method	nsnp	b	se	pval
MR Egger	3	5.793	3.036	0.307
Weighted median	3	0.260	0.131	0.047
Inverse variance weighted	3	0.300	0.135	0.026
Simple mode	3	0.015	0.215	0.949
Weighted mode	3	0.444	0.150	0.097

method	nsnp	b	se	pval
MR Egger	8	1.381	1.428	0.371
Weighted median	8	0.132	0.072	0.065
Inverse variance weighted	8	0.110	0.054	0.040
Simple mode	8	0.115	0.104	0.307
Weighted mode	8	0.120	0.092	0.234

method	nsnp	b	se	pval
MR Egger	3	2.420	5.025	0.714
Weighted median	3	-0.348	0.150	0.021
Inverse variance weighted	3	-0.371	0.111	0.001
Simple mode	3	-0.222	0.197	0.377
Weighted mode	3	-0.337	0.181	0.203

method	nsnp	b	se	pval
MR Egger	4	5.233	5.167	0.418
Weighted median	4	-0.237	0.124	0.056
Inverse variance weighted	4	-0.261	0.112	0.020
Simple mode	4	-0.508	0.211	0.095
Weighted mode	4	-0.085	0.135	0.573

method	nsnp	b	se	pval
MR Egger	4	-5.973	3.495	0.230
Weighted median	4	0.229	0.106	0.030
Inverse variance weighted	4	0.169	0.082	0.039
Simple mode	4	0.226	0.129	0.177
Weighted mode	4	0.226	0.113	0.140

method	nsnp	b	se	pval
MR Egger	4	-1.633	5.247	0.785
Weighted median	4	-0.288	0.146	0.049
Inverse variance weighted	4	-0.305	0.107	0.004
Simple mode	4	-0.312	0.163	0.152
Weighted mode	4	-0.280	0.136	0.133

method	nsnp	b	se	pval
MR Egger	3	-3.986	4.813	0.560
Weighted median	3	-0.300	0.145	0.038
Inverse variance weighted	3	-0.303	0.119	0.011
Simple mode	3	-0.312	0.196	0.253
Weighted mode	3	-0.306	0.144	0.167

method	nsnp	b	se	pval
MR Egger	4	-22.116	6.128	0.069
Weighted median	4	-0.165	0.202	0.413
Inverse variance weighted	4	-0.483	0.215	0.025
Simple mode	4	-0.190	0.413	0.677
Weighted mode	4	0.017	0.218	0.943

method	nsnp	b	se	pval
MR Egger	4	11.650	14.767	0.513
Weighted median	4	0.279	0.142	0.050
Inverse variance weighted	4	0.281	0.109	0.010
Simple mode	4	0.238	0.178	0.275
Weighted mode	4	0.272	0.129	0.125

method	nsnp	b	se	pval
MR Egger	4	-1.093	1.746	0.595
Weighted median	4	0.228	0.123	0.063
Inverse variance weighted	4	0.232	0.102	0.023
Simple mode	4	0.151	0.148	0.383
Weighted mode	4	0.172	0.134	0.291

method	nsnp	b	se	pval
MR Egger	4	16.172	12.328	0.320
Weighted median	4	0.290	0.111	0.009
Inverse variance weighted	4	0.232	0.104	0.025
Simple mode	4	0.312	0.144	0.119
Weighted mode	4	0.293	0.133	0.114

method	nsnp	b	se	pval
MR Egger	5	0.623	1.848	0.758
Weighted median	5	-0.192	0.102	0.059
Inverse variance weighted	5	-0.169	0.080	0.035
Simple mode	5	-0.219	0.138	0.187
Weighted mode	5	-0.229	0.149	0.198

method	nsnp	b	se	pval
MR Egger	5	1.371	0.998	0.263
Weighted median	5	0.155	0.092	0.092
Inverse variance weighted	5	0.173	0.074	0.020
Simple mode	5	0.160	0.123	0.263
Weighted mode	5	0.141	0.110	0.267

method	nsnp	b	se	pval
MR Egger	3	-0.538	2.829	0.880
Weighted median	3	-0.273	0.130	0.035
Inverse variance weighted	3	-0.280	0.105	0.008
Simple mode	3	-0.282	0.140	0.181
Weighted mode	3	-0.267	0.128	0.172

method	nsnp	b	se	pval
MR Egger	4	-3.036	4.157	0.541
Weighted median	4	0.172	0.108	0.113
Inverse variance weighted	4	0.205	0.087	0.018
Simple mode	4	0.141	0.135	0.373
Weighted mode	4	0.162	0.118	0.261

method	nsnp	b	se	pval
MR Egger	4	-6.685	6.686	0.423
Weighted median	4	0.195	0.153	0.203
Inverse variance weighted	4	0.265	0.106	0.013
Simple mode	4	0.073	0.242	0.784
Weighted mode	4	0.417	0.173	0.095

method	nsnp	b	se	pval
MR Egger	3	0.885	2.399	0.775
Weighted median	3	0.190	0.122	0.118
Inverse variance weighted	3	0.200	0.100	0.046
Simple mode	3	0.198	0.140	0.293
Weighted mode	3	0.184	0.122	0.271

method	nsnp	b	se	pval
MR Egger	3	-1.009	5.870	0.892
Weighted median	3	-0.324	0.164	0.048
Inverse variance weighted	3	-0.298	0.133	0.025
Simple mode	3	-0.325	0.199	0.244
Weighted mode	3	-0.325	0.154	0.169

method	nsnp	b	se	pval
MR Egger	6	1.995	2.533	0.475
Weighted median	6	0.163	0.111	0.142
Inverse variance weighted	6	0.194	0.087	0.026
Simple mode	6	0.134	0.165	0.452
Weighted mode	6	0.130	0.142	0.402

method	nsnp	b	se	pval
MR Egger	4	1.576	2.415	0.581
Weighted median	4	-0.285	0.126	0.023
Inverse variance weighted	4	-0.233	0.119	0.050
Simple mode	4	-0.255	0.161	0.210
Weighted mode	4	-0.294	0.133	0.115

method	nsnp	b	se	pval
MR Egger	3	-9.144	6.762	0.405
Weighted median	3	0.328	0.182	0.071
Inverse variance weighted	3	0.381	0.176	0.030
Simple mode	3	0.417	0.304	0.304
Weighted mode	3	0.238	0.213	0.380

method	nsnp	b	se	pval
MR Egger	5	1.899	1.512	0.298
Weighted median	5	0.129	0.086	0.131
Inverse variance weighted	5	0.151	0.069	0.029
Simple mode	5	0.121	0.112	0.340
Weighted mode	5	0.119	0.102	0.311

method	nsnp	b	se	pval
MR Egger	4	-1.295	3.037	0.711
Weighted median	4	0.189	0.119	0.113
Inverse variance weighted	4	0.208	0.091	0.022
Simple mode	4	0.116	0.149	0.495
Weighted mode	4	0.174	0.117	0.234

method	nsnp	b	se	pval
MR Egger	3	-9.986	13.914	0.604
Weighted median	3	0.172	0.117	0.142
Inverse variance weighted	3	0.190	0.090	0.036
Simple mode	3	0.178	0.141	0.336
Weighted mode	3	0.144	0.145	0.427

method	nsnp	b	se	pval
MR Egger	4	-6.983	8.580	0.501
Weighted median	4	-0.292	0.115	0.011
Inverse variance weighted	4	-0.321	0.083	0.000
Simple mode	4	-0.261	0.140	0.159
Weighted mode	4	-0.268	0.138	0.148

method	nsnp	b	se	pval
MR Egger	4	-1.958	6.725	0.798
Weighted median	4	0.179	0.170	0.294
Inverse variance weighted	4	0.332	0.138	0.016
Simple mode	4	0.205	0.235	0.447
Weighted mode	4	0.168	0.192	0.446

method	nsnp	b	se	pval
MR Egger	5	4.608	4.283	0.361
Weighted median	5	0.241	0.115	0.036
Inverse variance weighted	5	0.253	0.089	0.005
Simple mode	5	0.237	0.157	0.205
Weighted mode	5	0.242	0.138	0.155

method	nsnp	b	se	pval
MR Egger	3	-13.197	10.701	0.434
Weighted median	3	0.145	0.139	0.297
Inverse variance weighted	3	0.228	0.112	0.041
Simple mode	3	0.147	0.197	0.532
Weighted mode	3	0.135	0.155	0.477

method	nsnp	b	se	pval
MR Egger	5	0.652	4.050	0.882
Weighted median	5	-0.324	0.189	0.087
Inverse variance weighted	5	-0.344	0.145	0.017
Simple mode	5	-0.183	0.222	0.457
Weighted mode	5	-0.321	0.206	0.195

method	nsnp	b	se	pval
MR Egger	3	-3.450	6.408	0.686
Weighted median	3	0.341	0.186	0.067
Inverse variance weighted	3	0.381	0.151	0.012
Simple mode	3	0.335	0.220	0.267
Weighted mode	3	0.320	0.195	0.242

method	nsnp	b	se	pval
MR Egger	3	6.469	34.600	0.882
Weighted median	3	0.515	0.267	0.054
Inverse variance weighted	3	0.514	0.236	0.029
Simple mode	3	0.655	0.449	0.282
Weighted mode	3	0.502	0.250	0.182

method	nsnp	b	se	pval
MR Egger	4	5.373	9.200	0.618
Weighted median	4	-0.406	0.179	0.024
Inverse variance weighted	4	-0.384	0.185	0.038
Simple mode	4	-0.036	0.339	0.922
Weighted mode	4	-0.607	0.320	0.154

method	nsnp	b	se	pval
MR Egger	5	-8.282	24.718	0.760
Weighted median	5	-0.272	0.146	0.063
Inverse variance weighted	5	-0.407	0.135	0.003
Simple mode	5	-0.265	0.184	0.222
Weighted mode	5	-0.265	0.156	0.164
