# Supplementary figures and images for: Unveiling the Relationship Between Oral Microbiota and Alzheimer's Disease: A Genetic Instrumental Variable Analysis via Mendelian Randomization
Source: Brain Behav. 2025 Aug 4;15(8):e70753. doi: 10.1002/brb3.70753 (PMC12321961; doi:10.1002/brb3.70753)

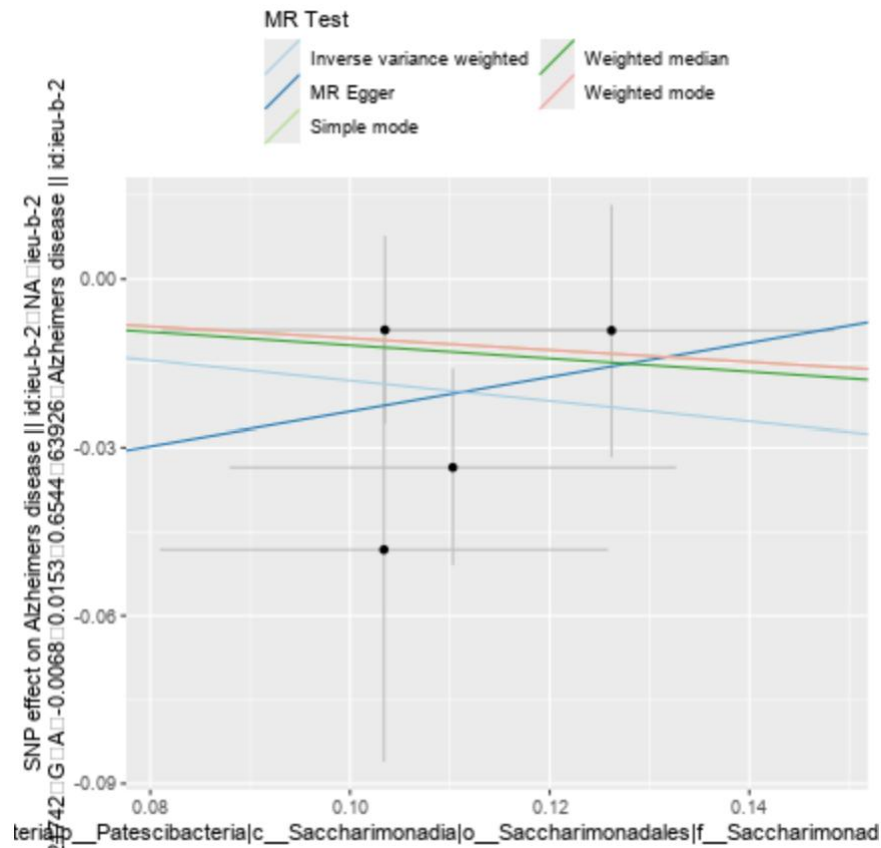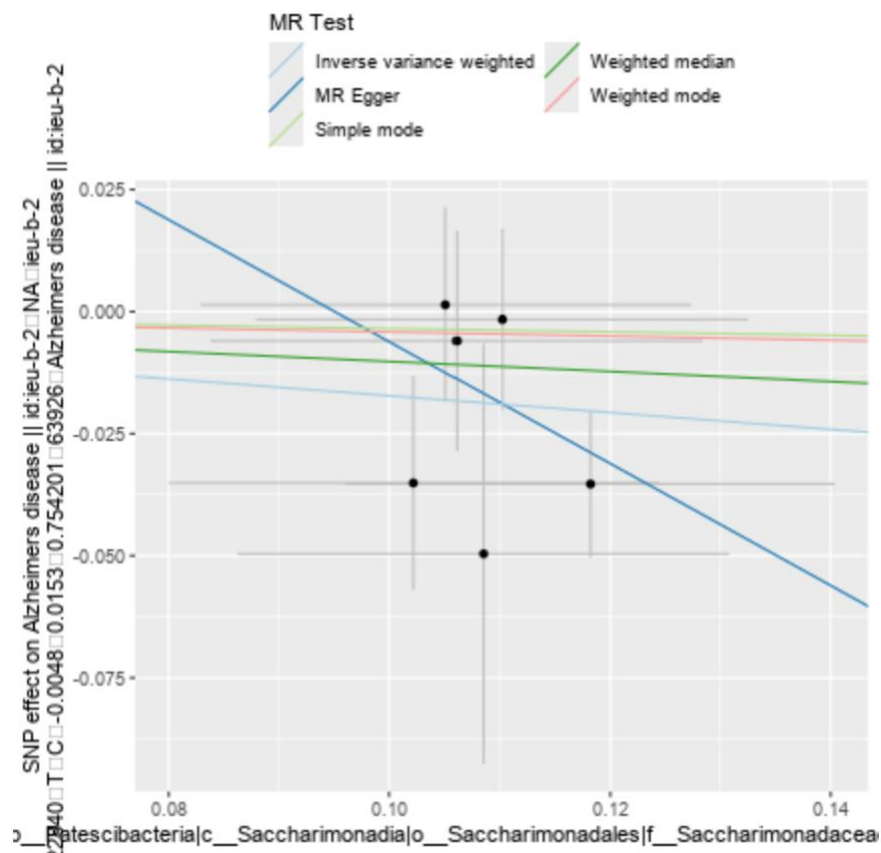

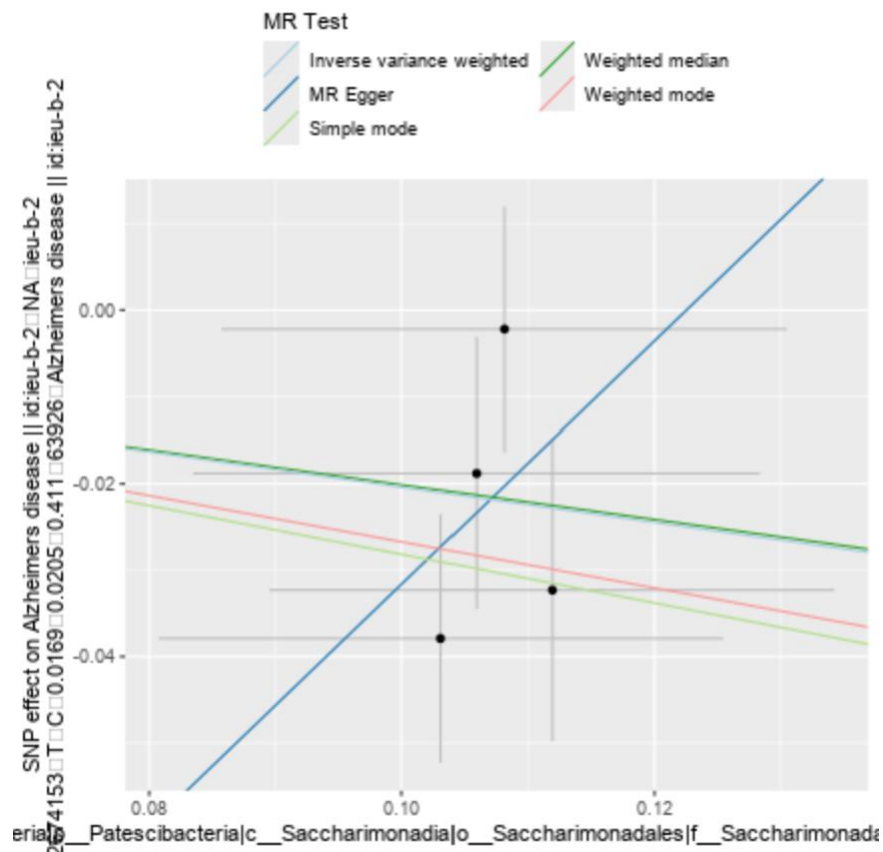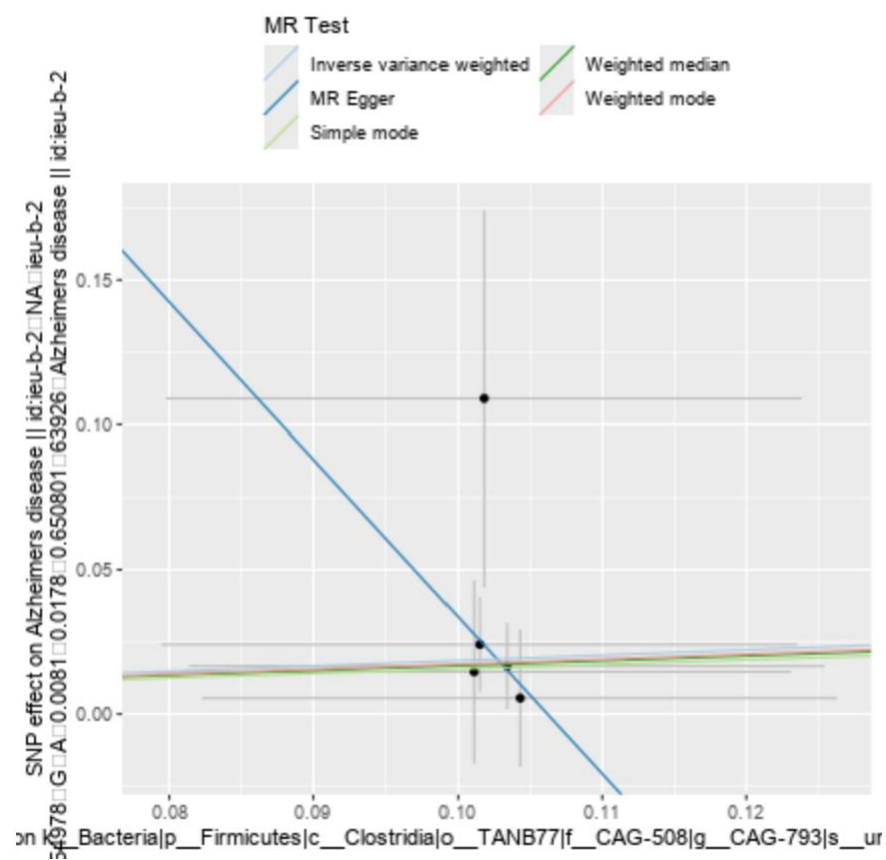



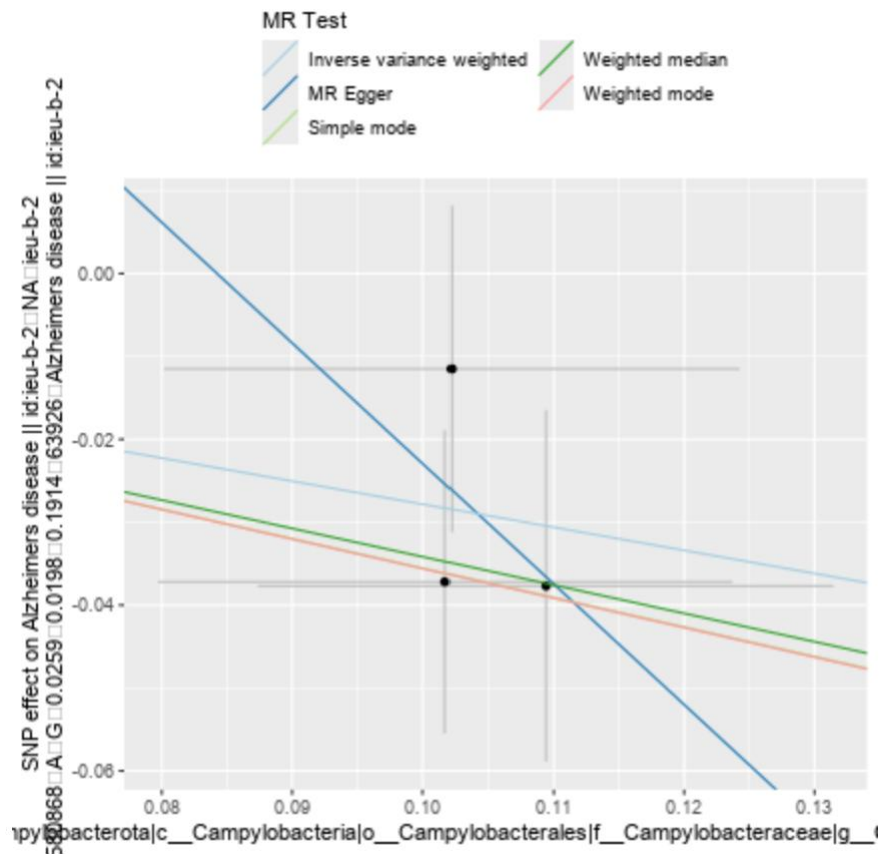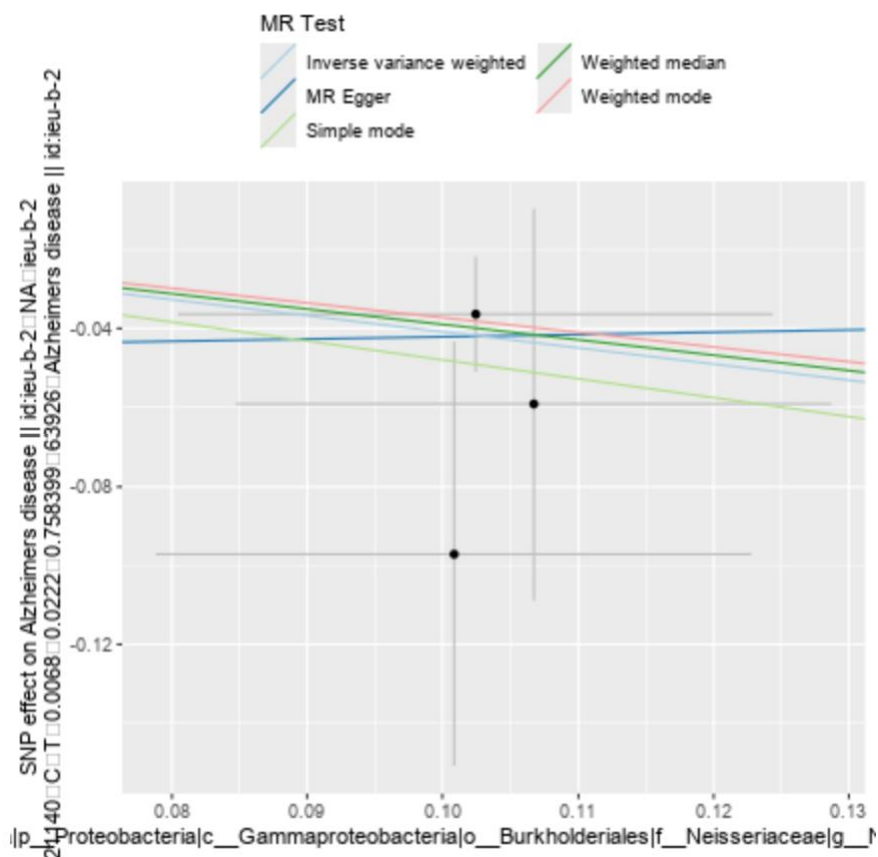





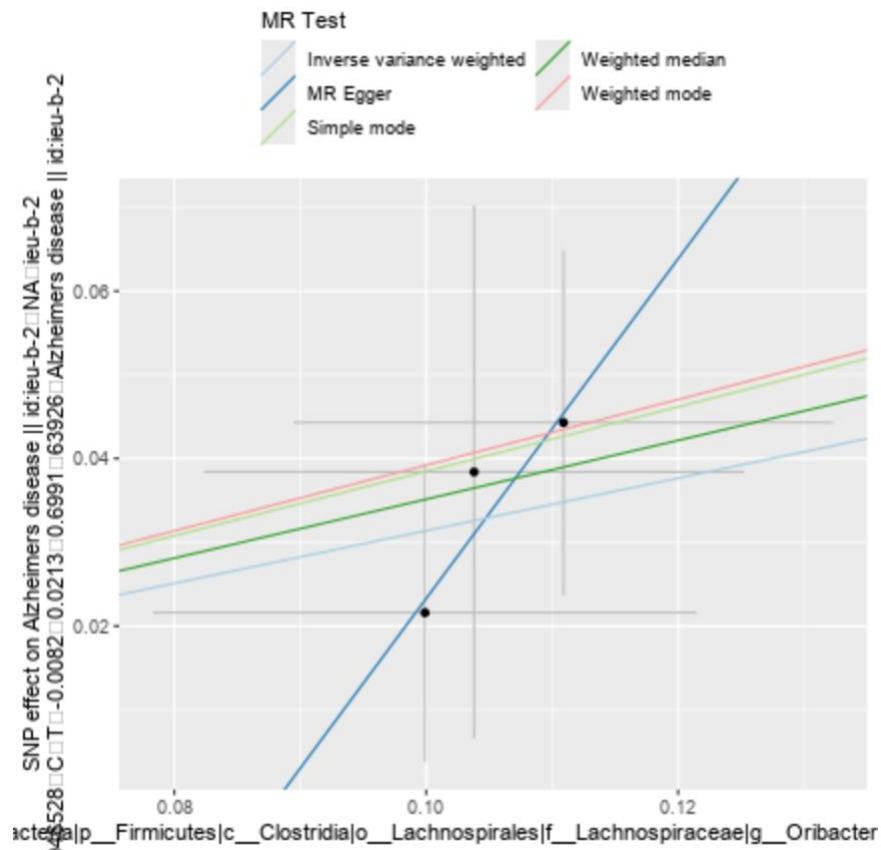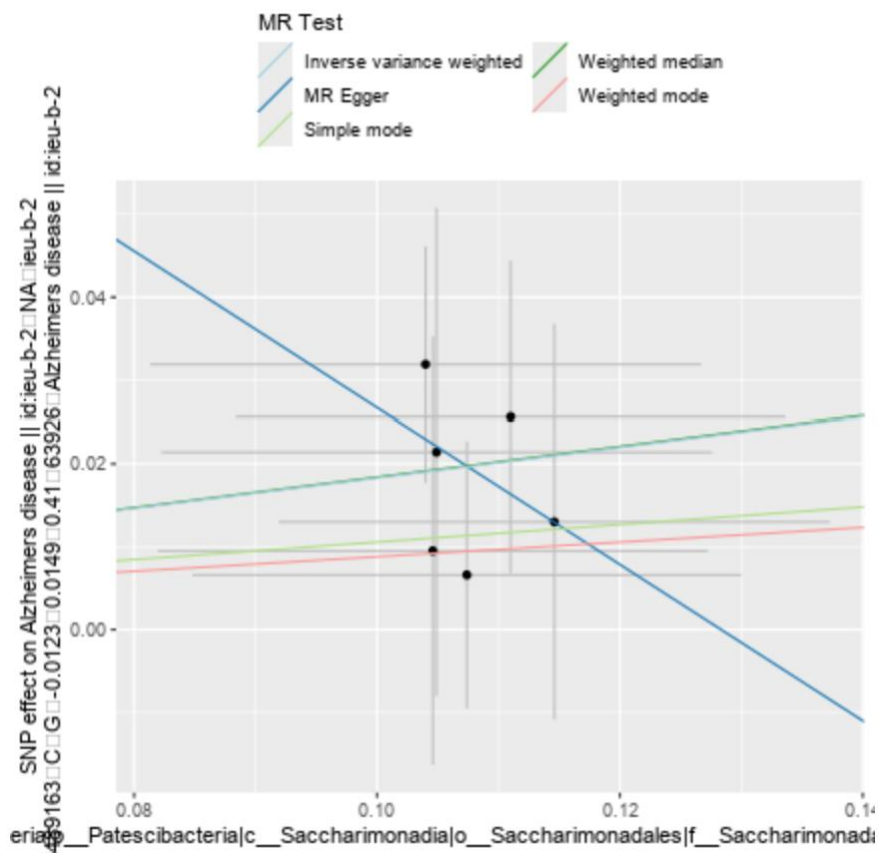

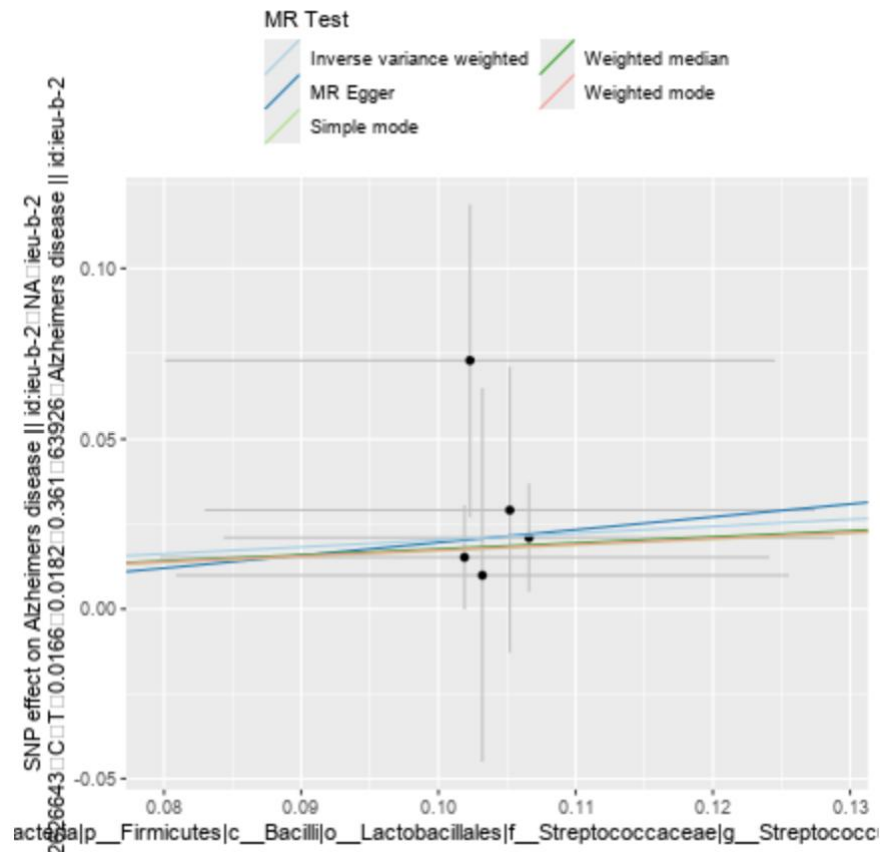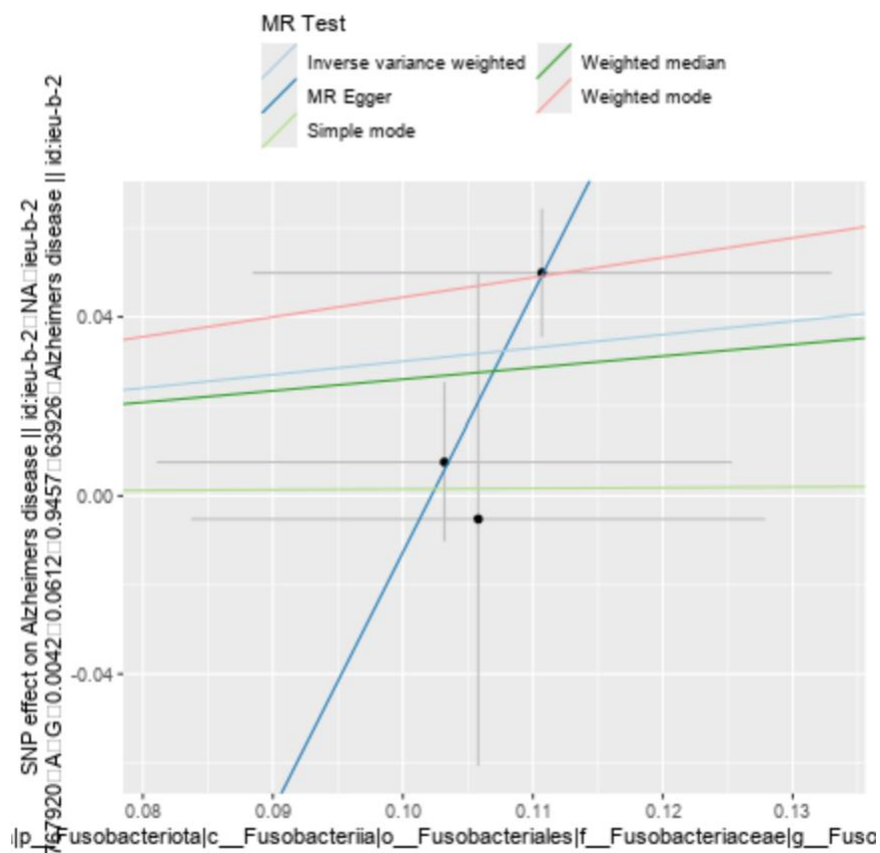



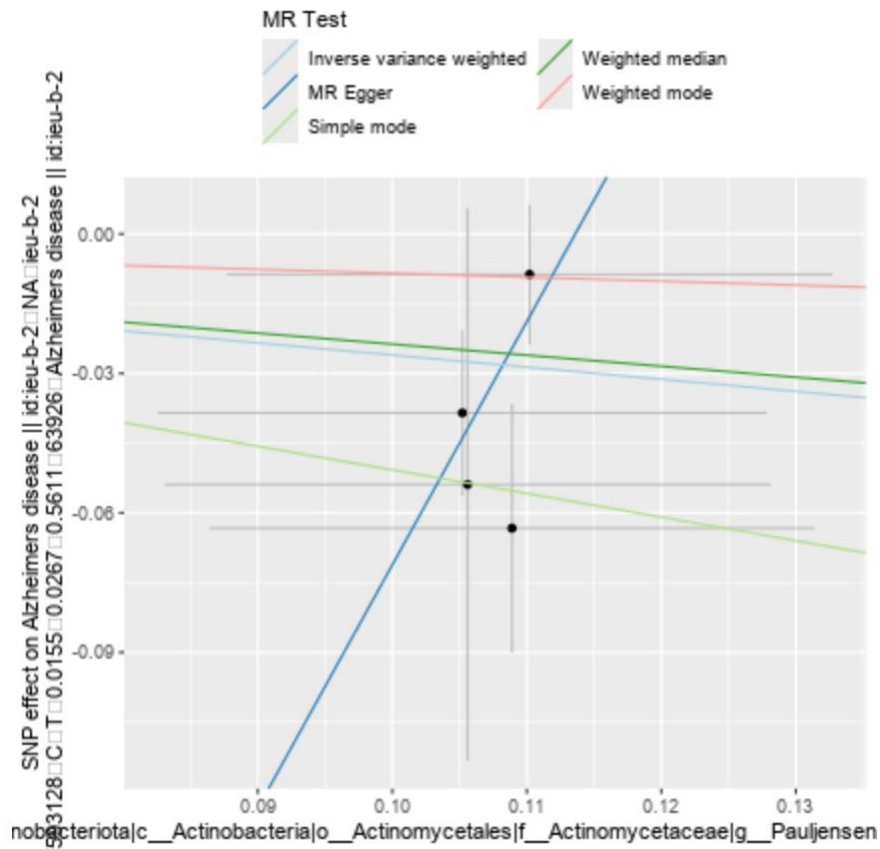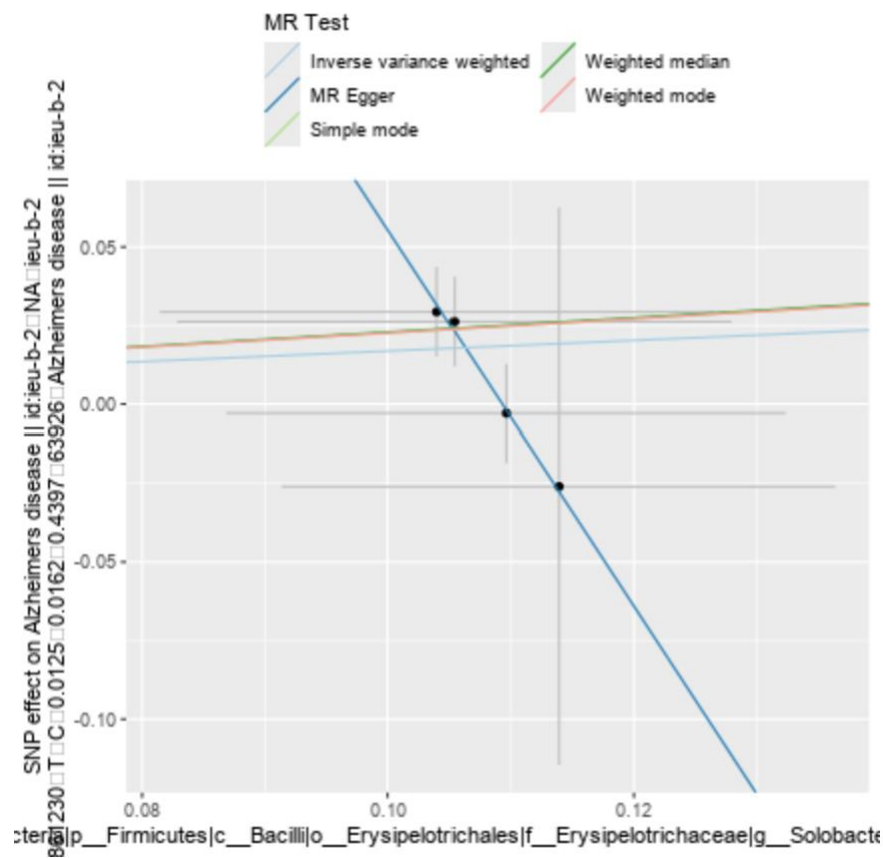

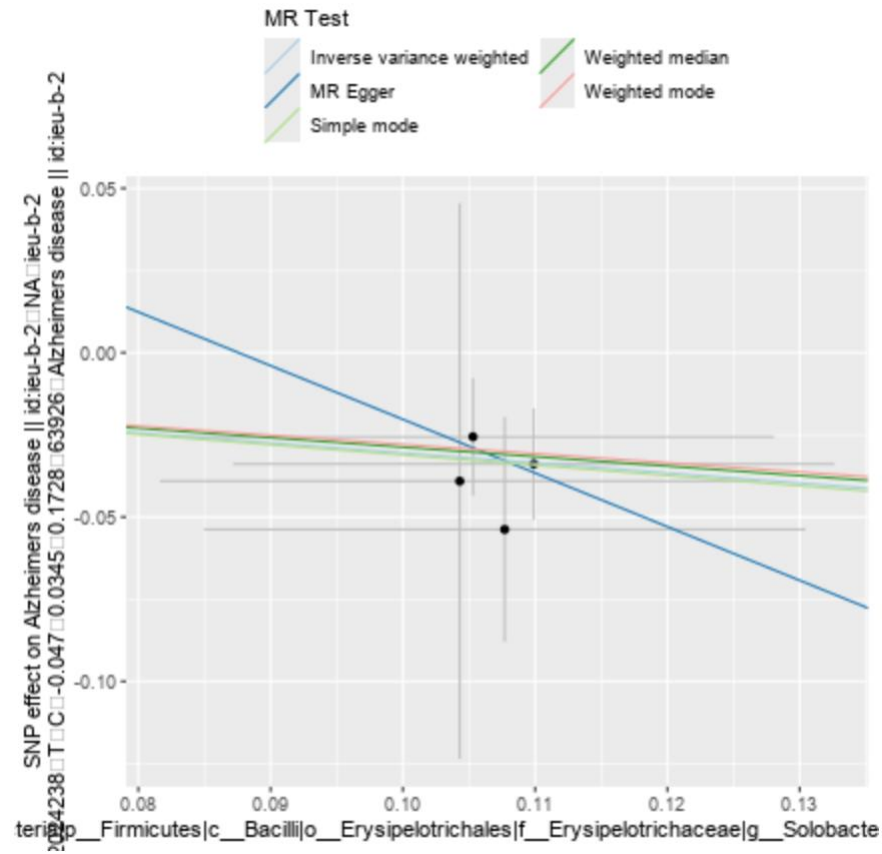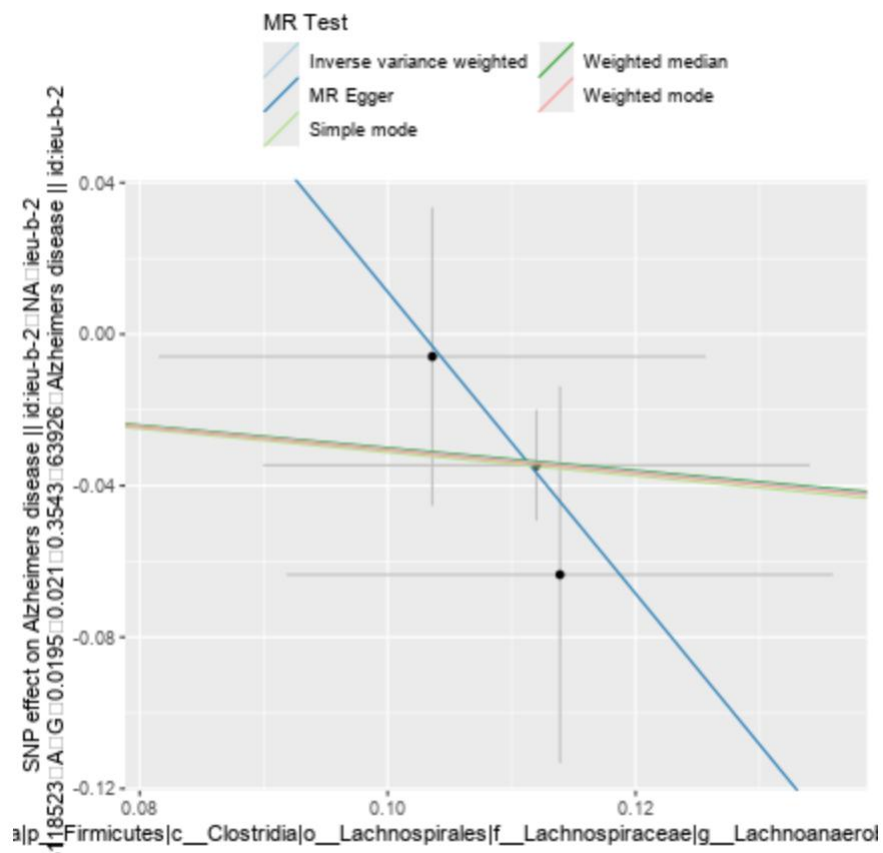

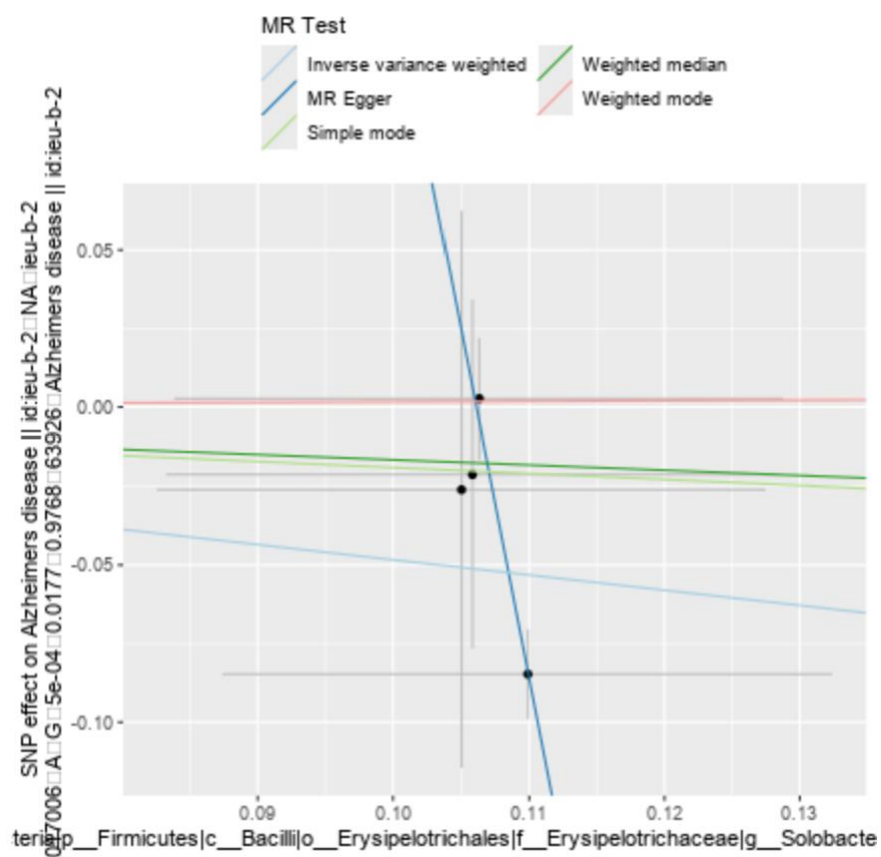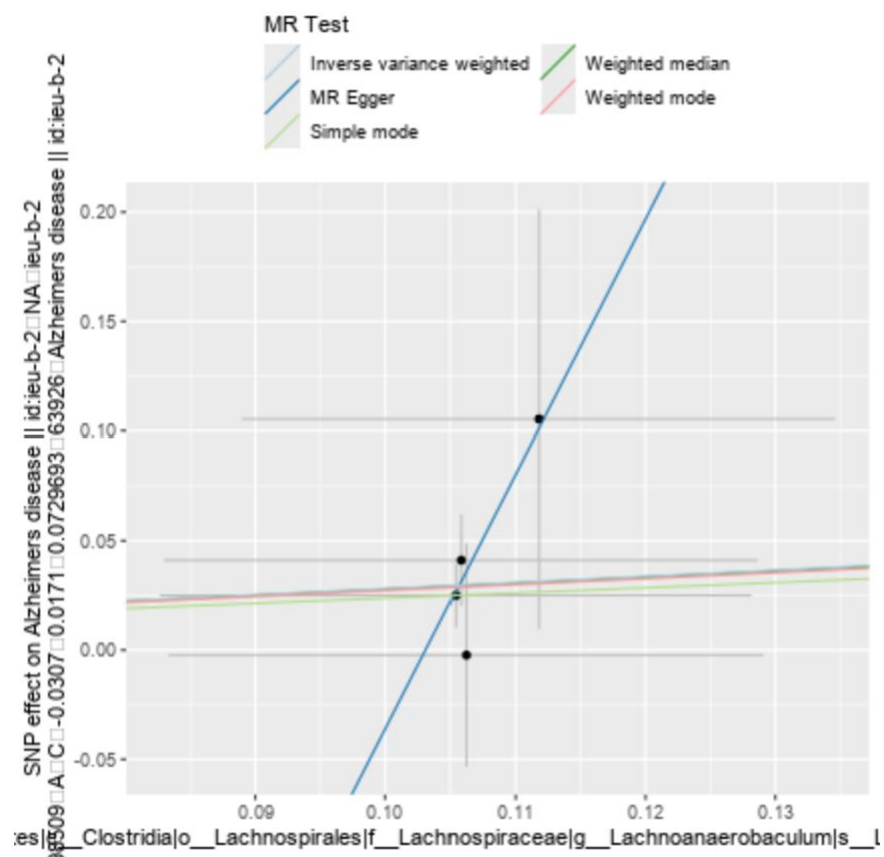

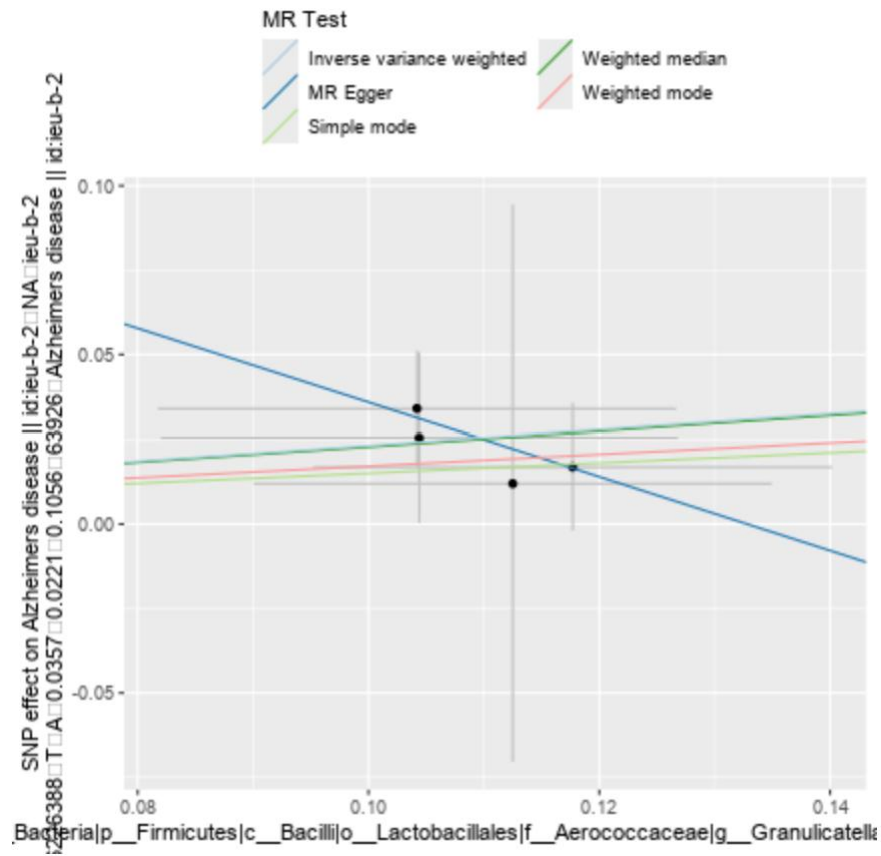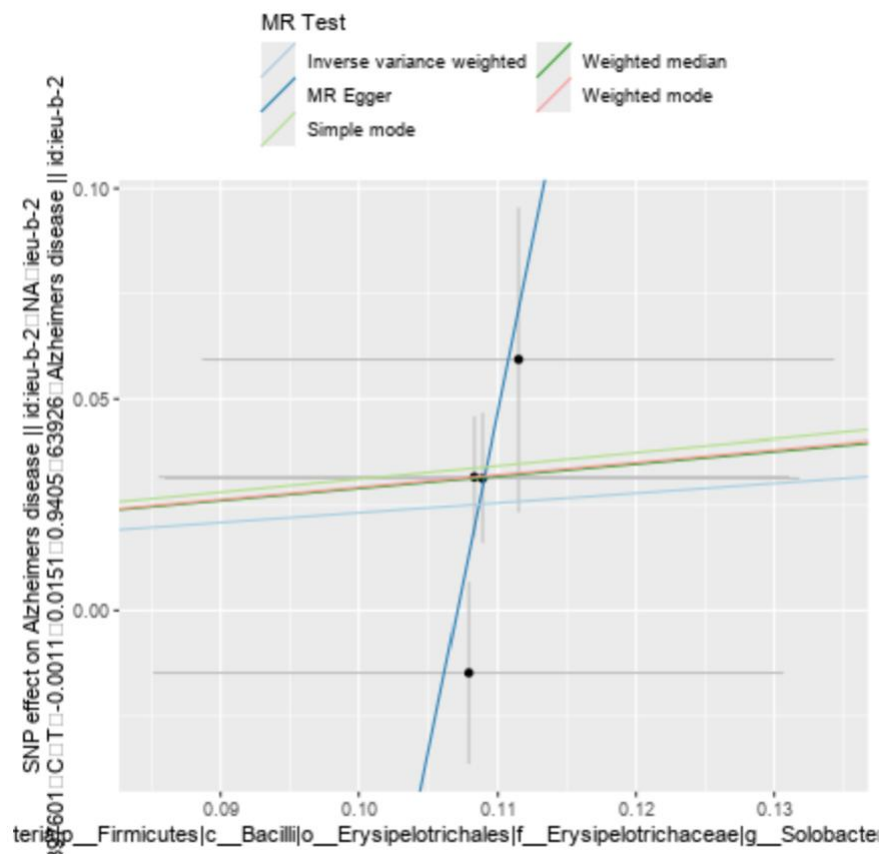



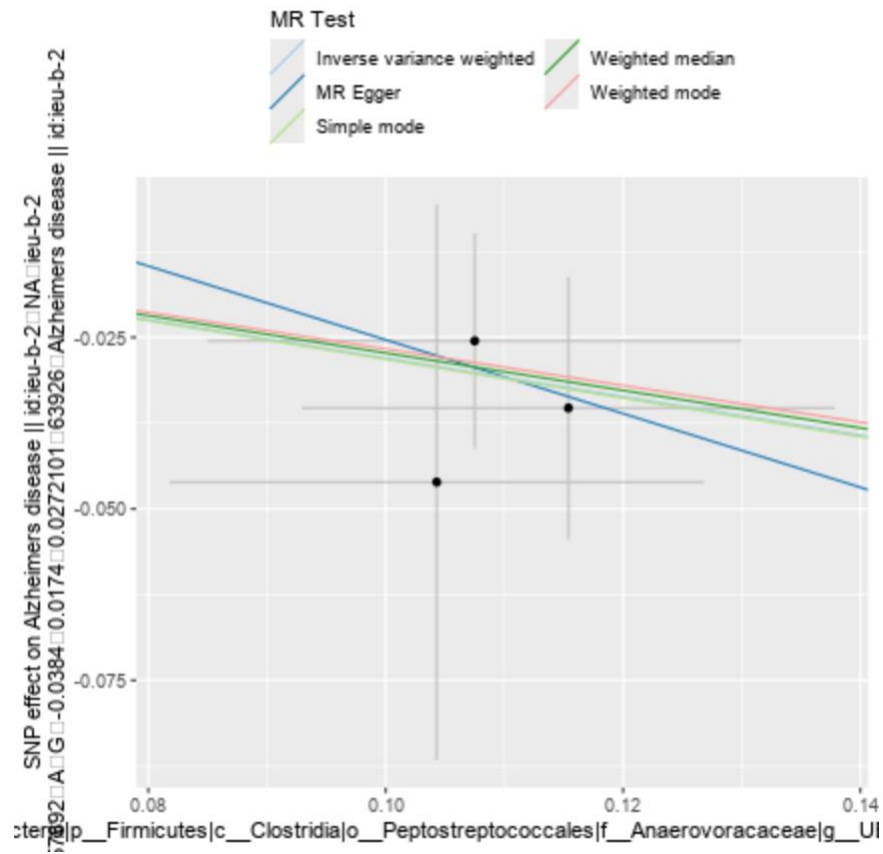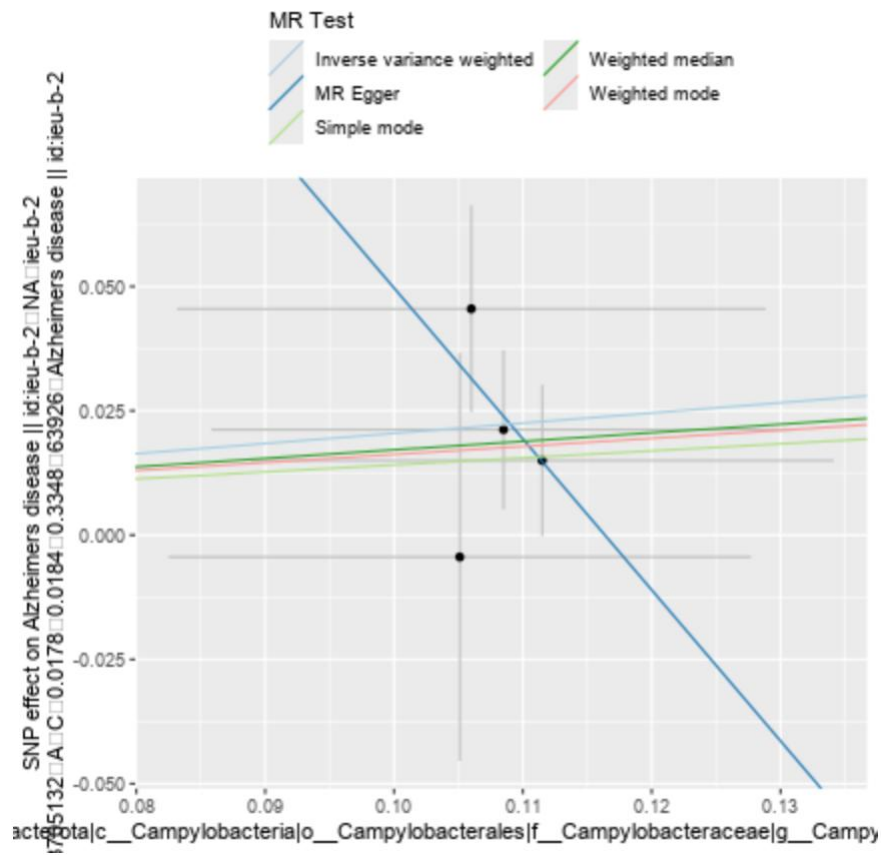



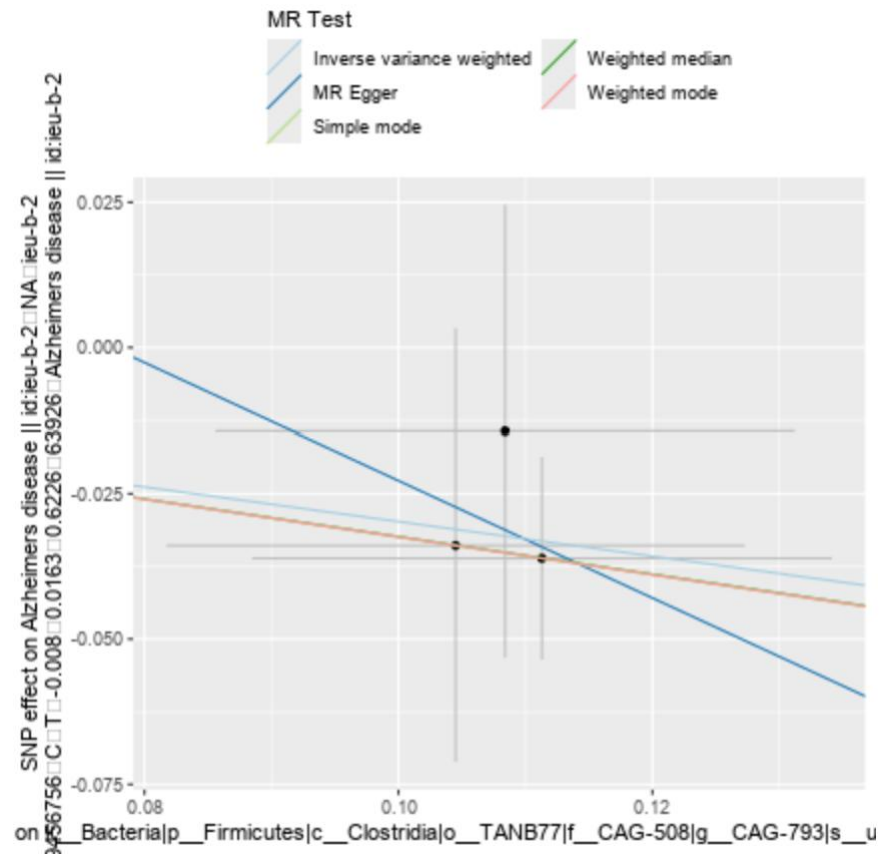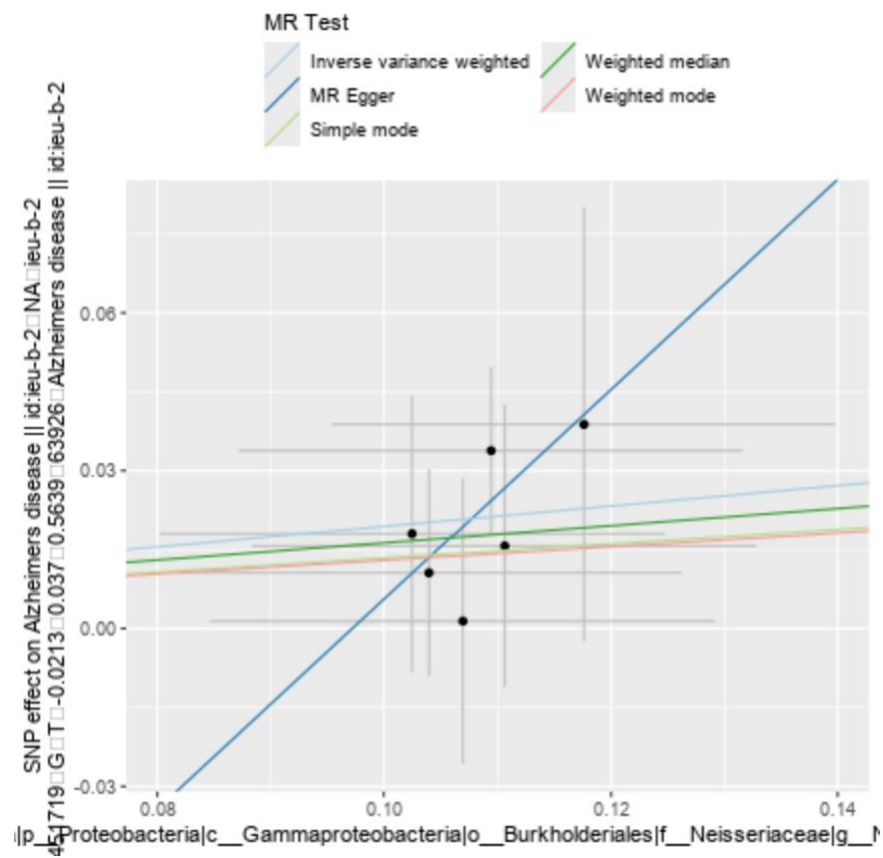



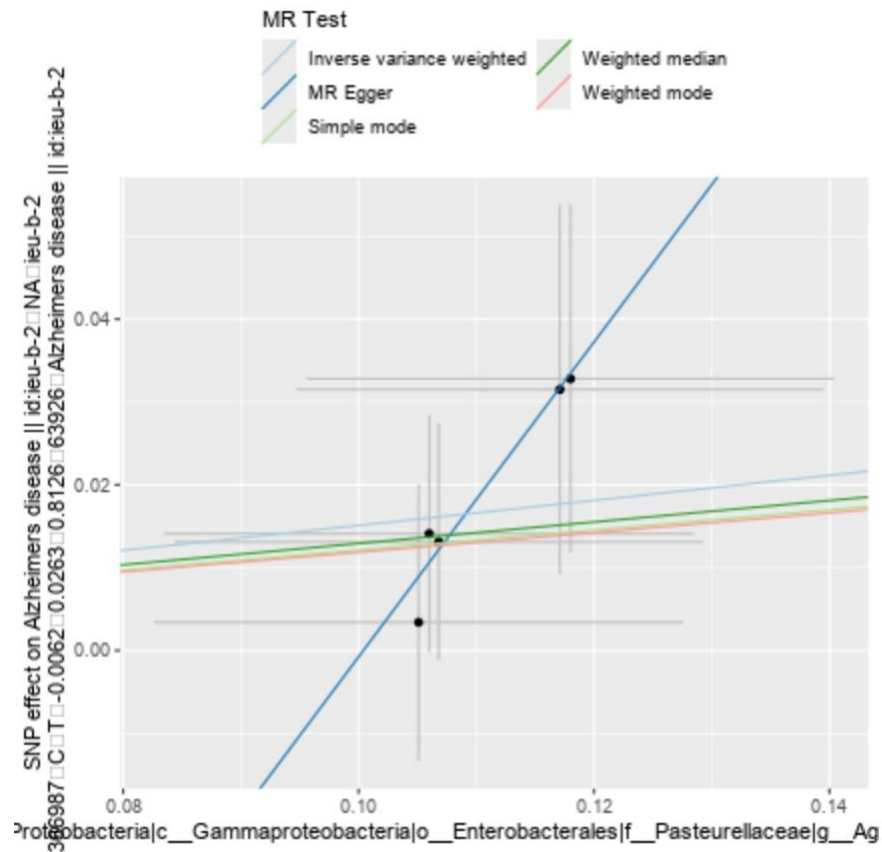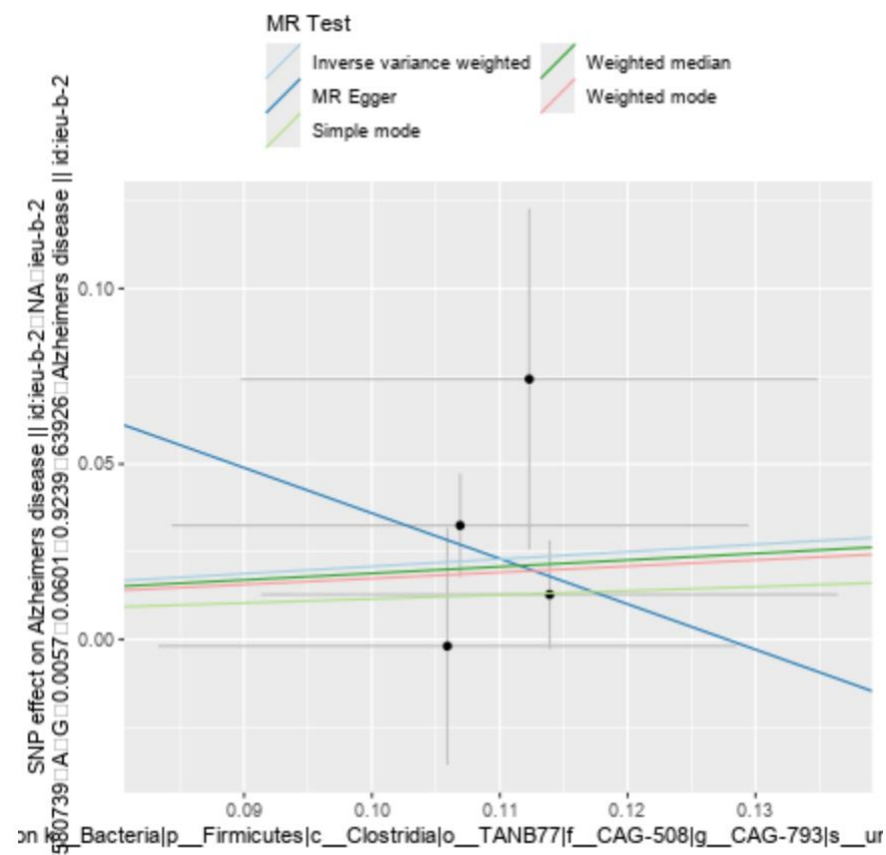



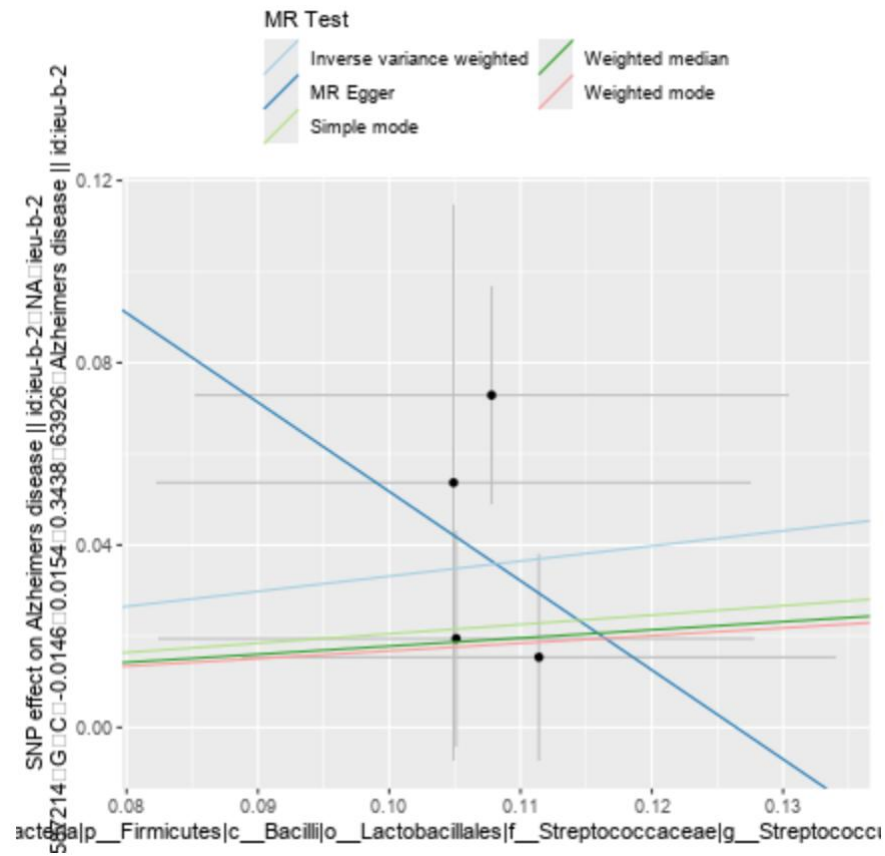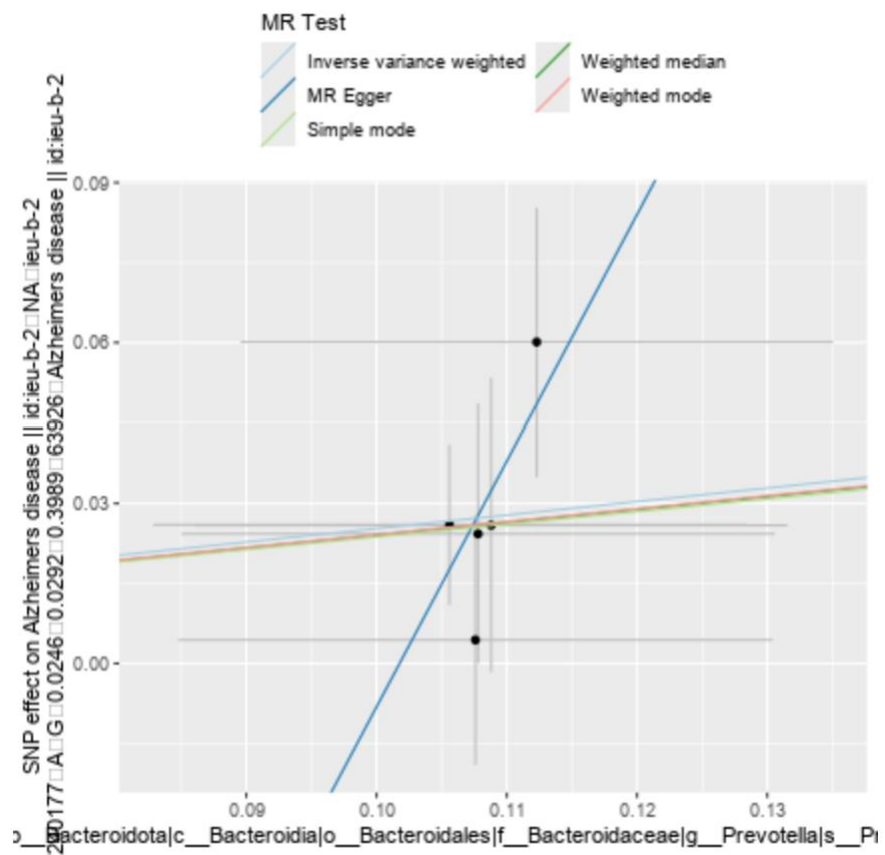

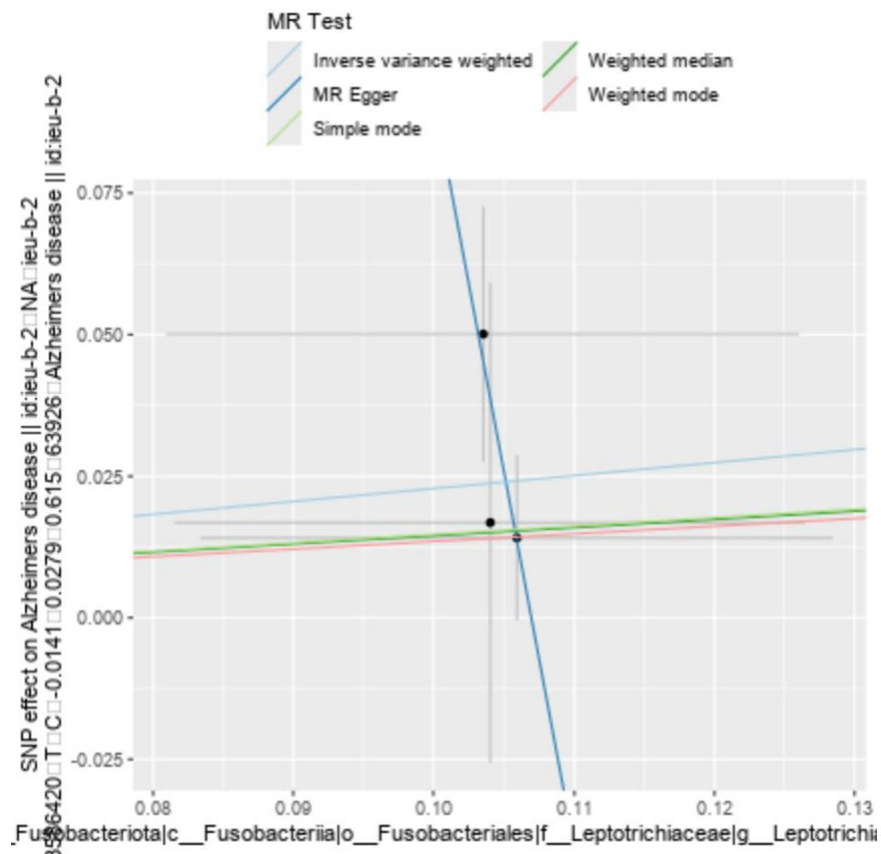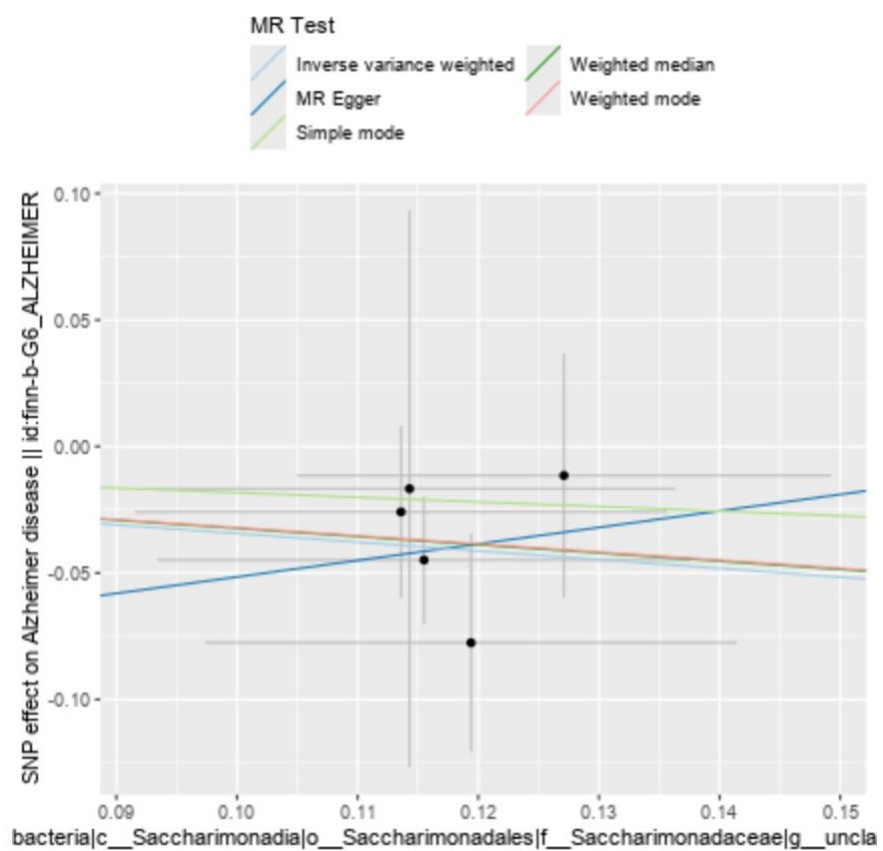

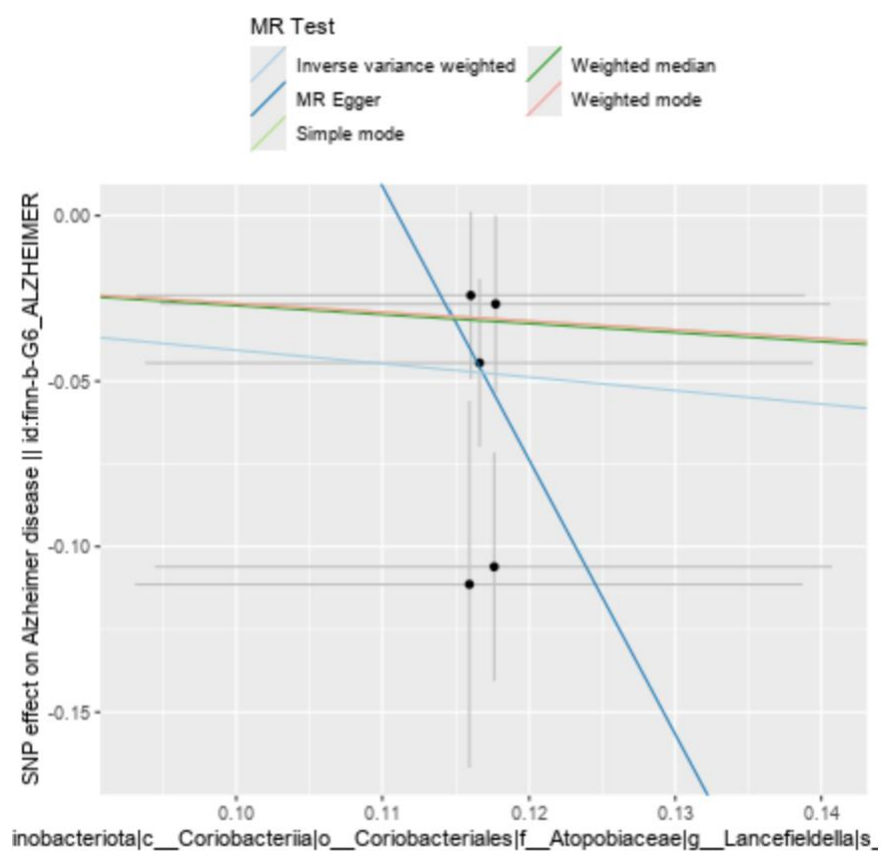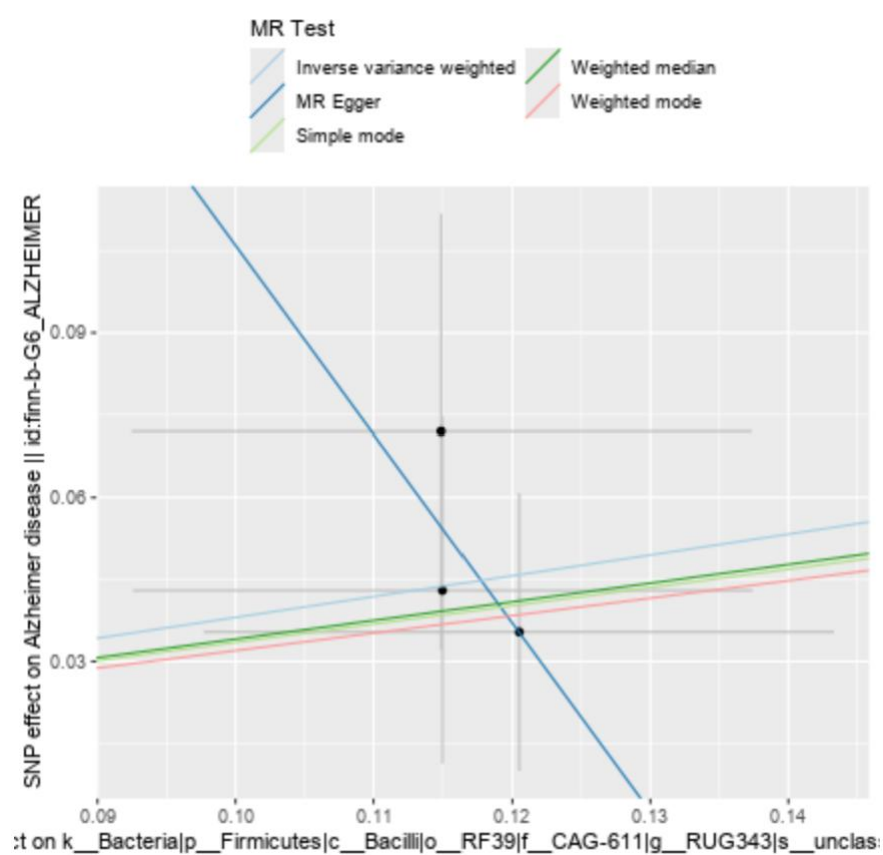

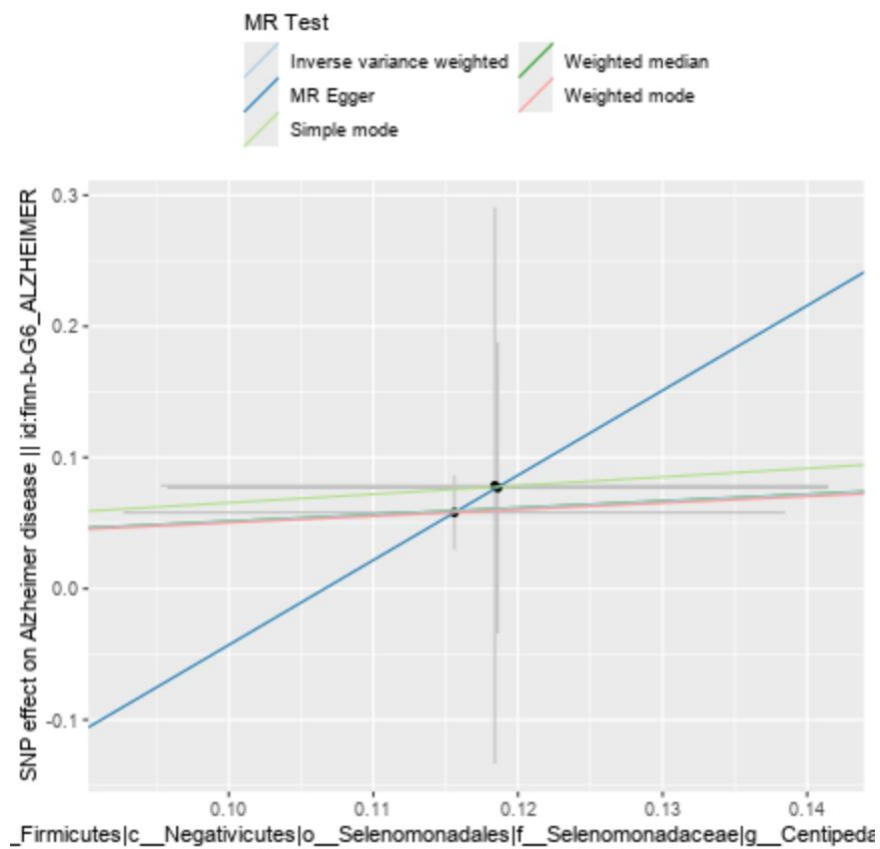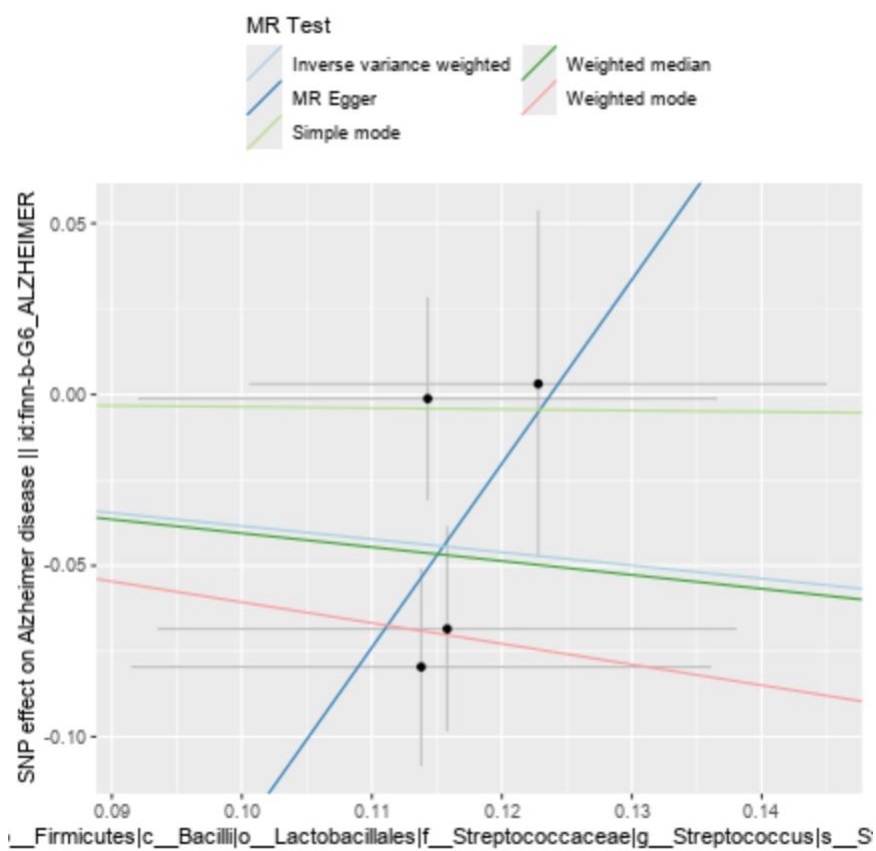

Supplement: Supplementary file 4 — Supplementary Information [file BRB3-15-e70753-s005.pdf]

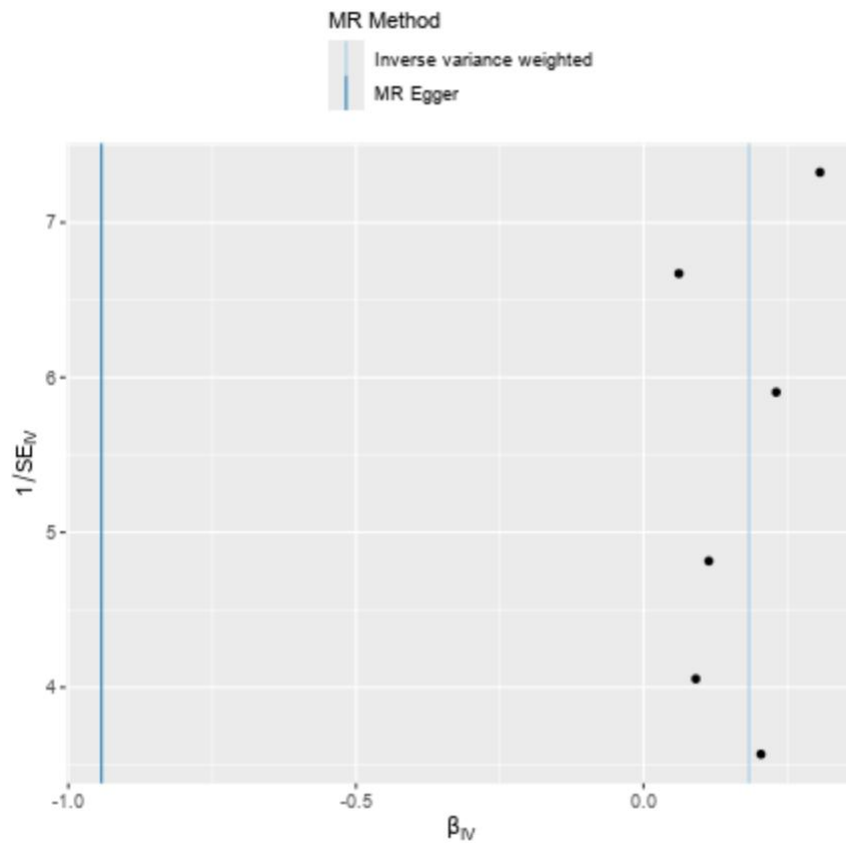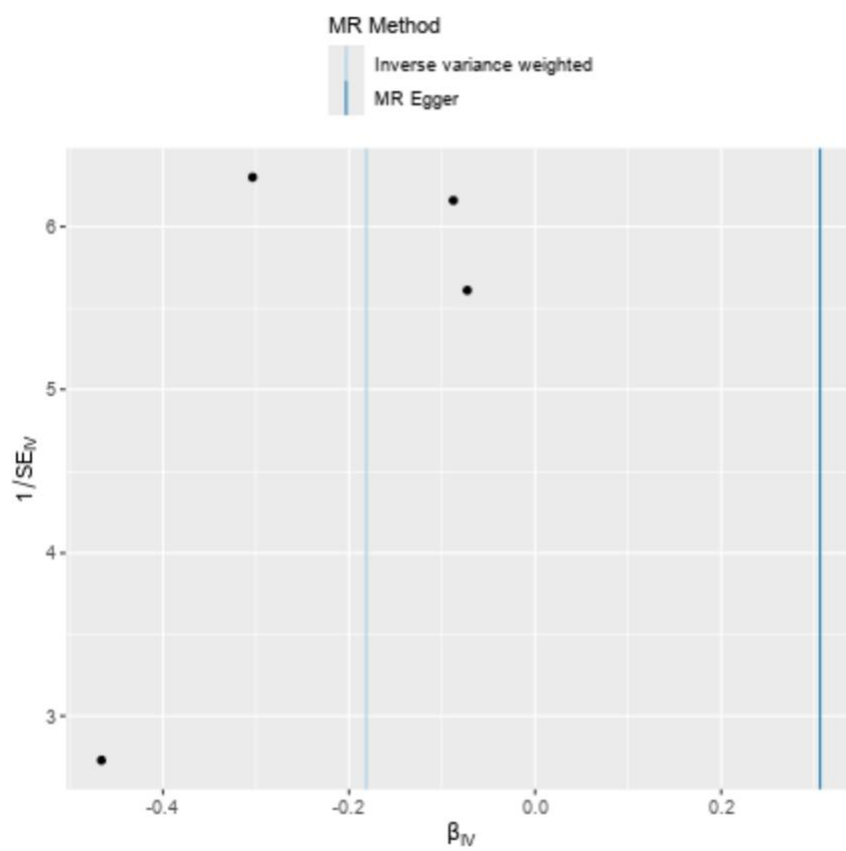

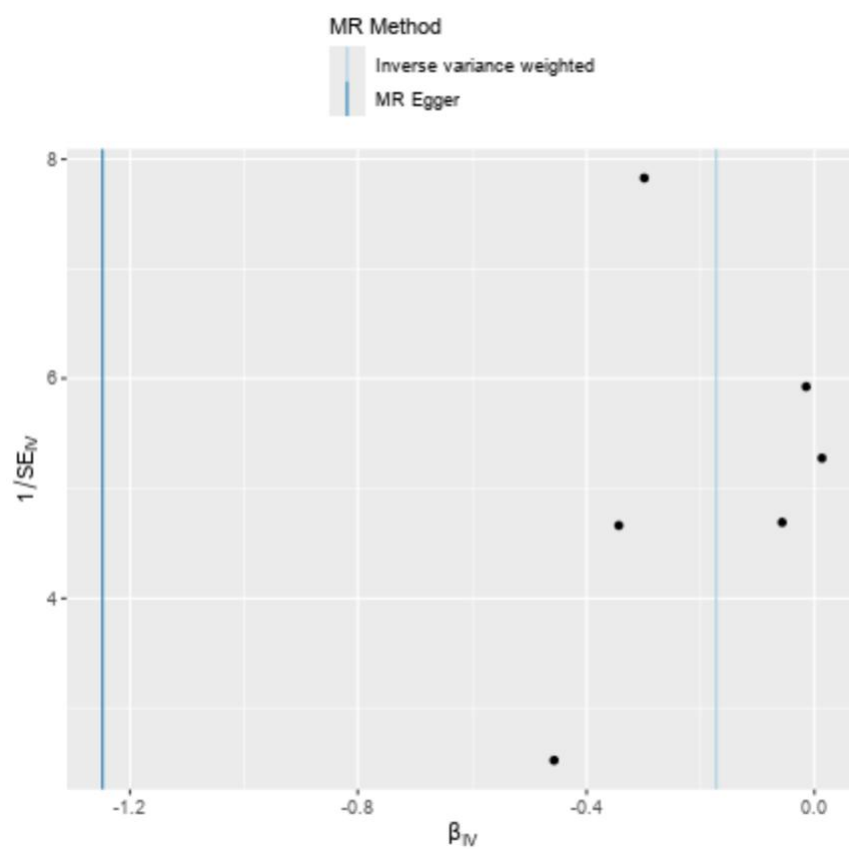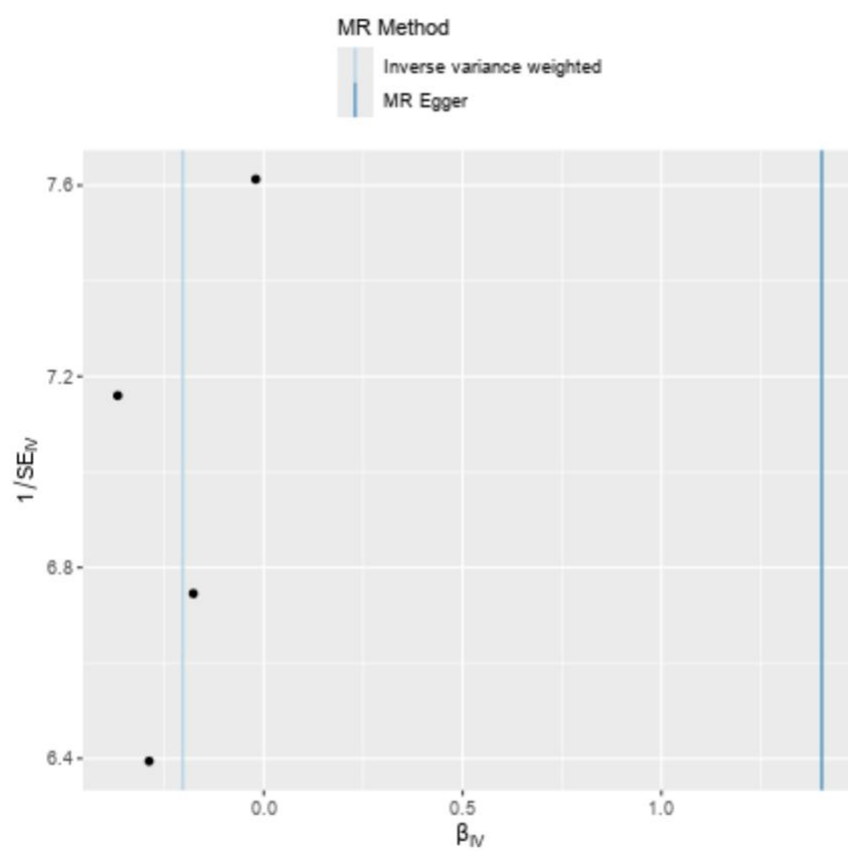

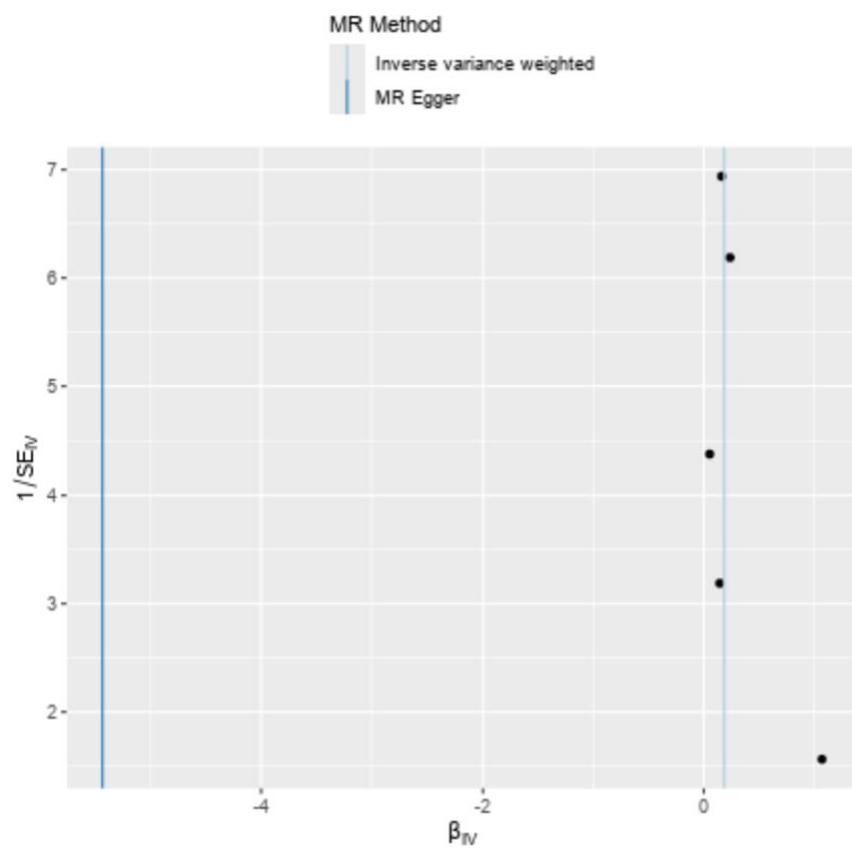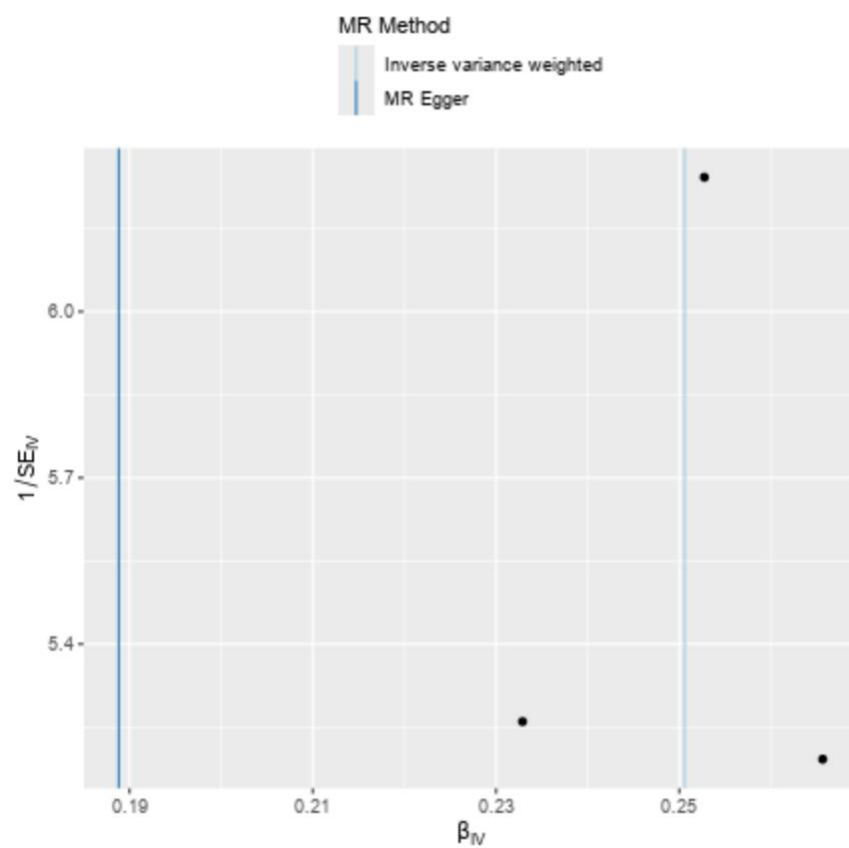

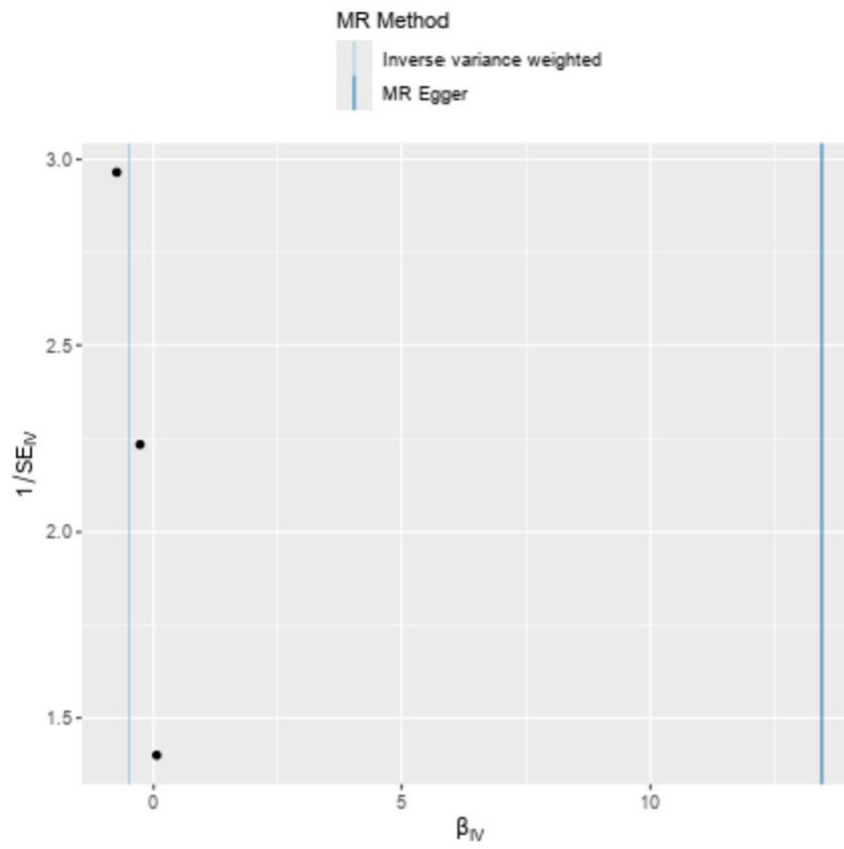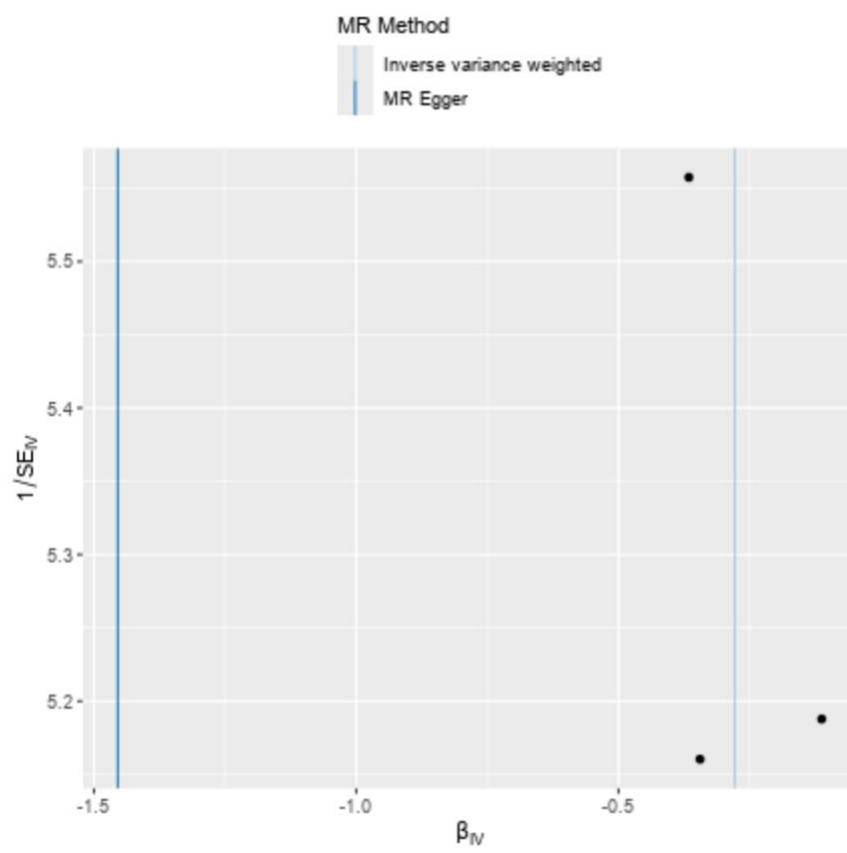

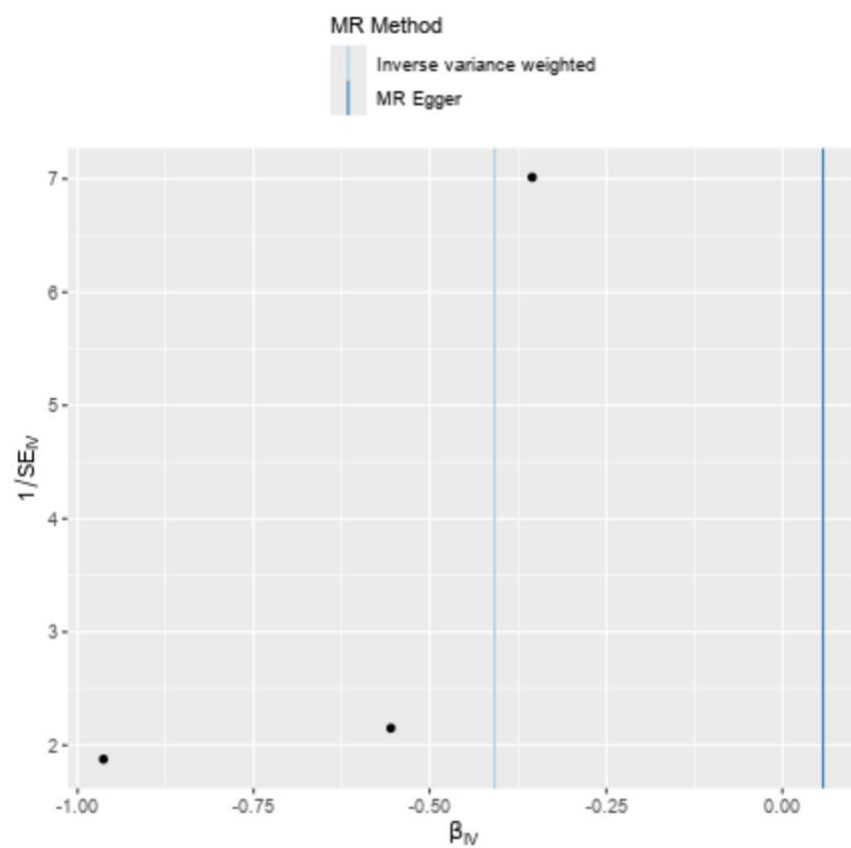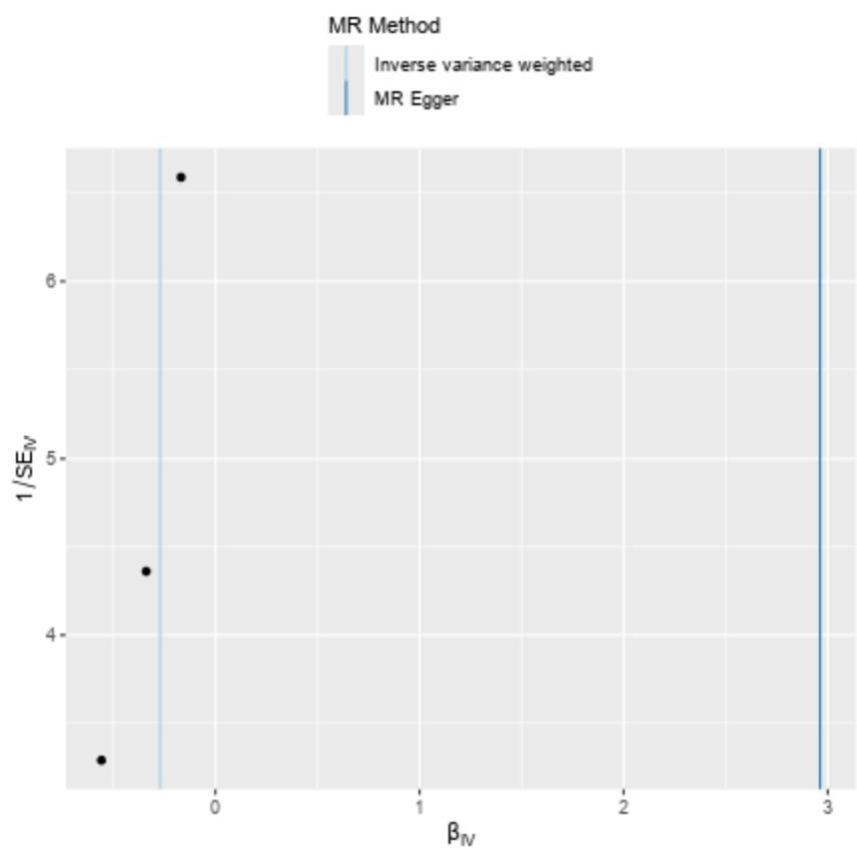

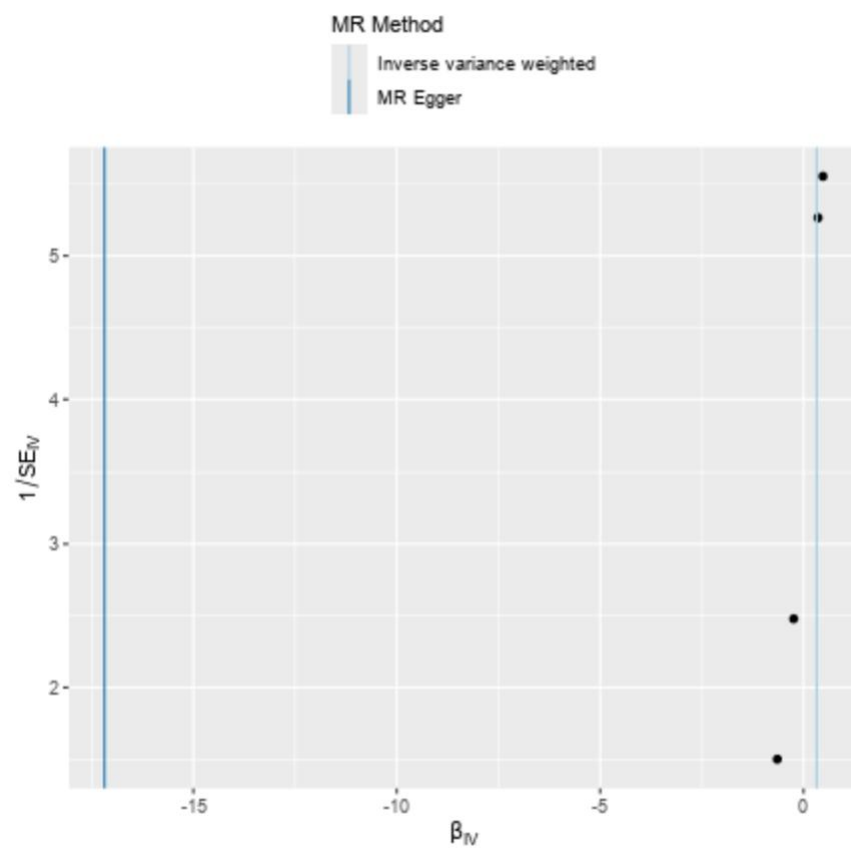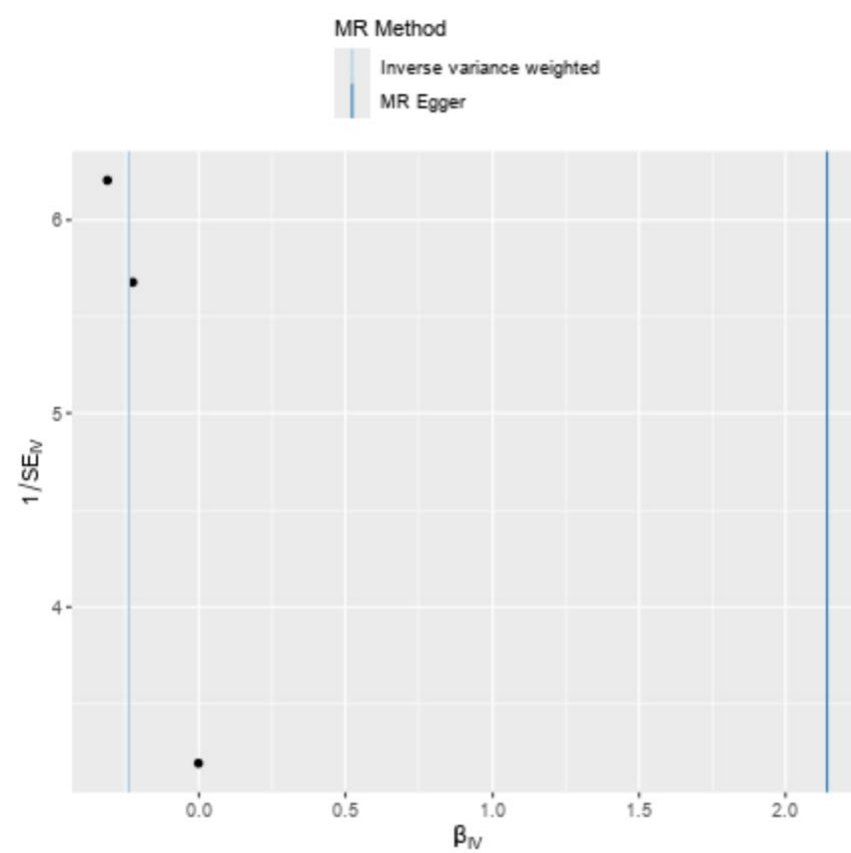

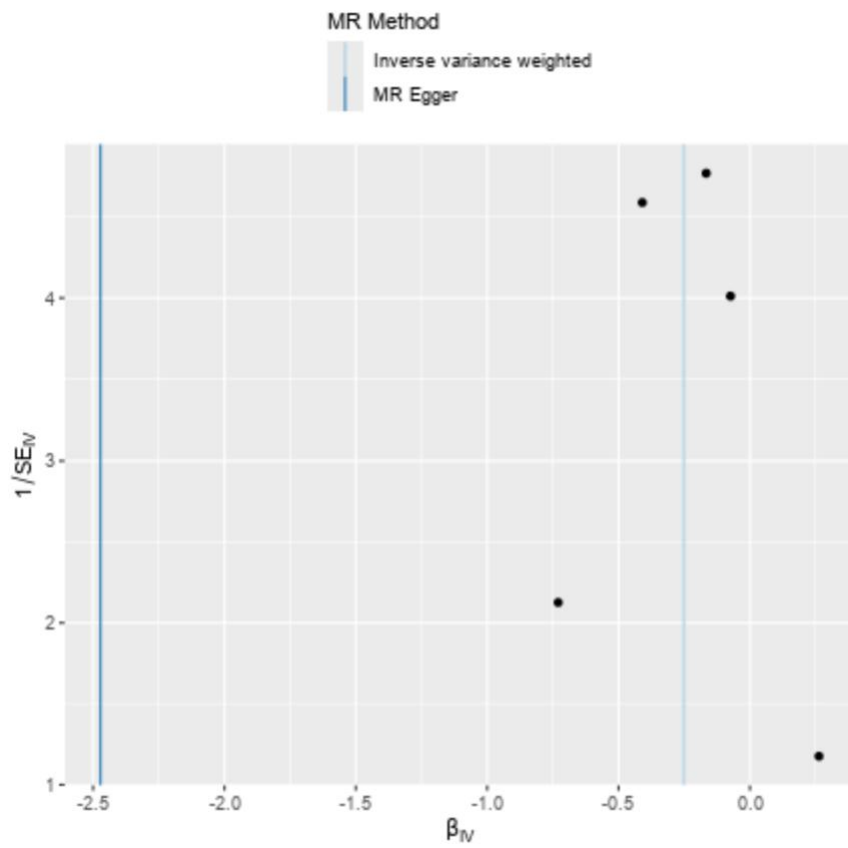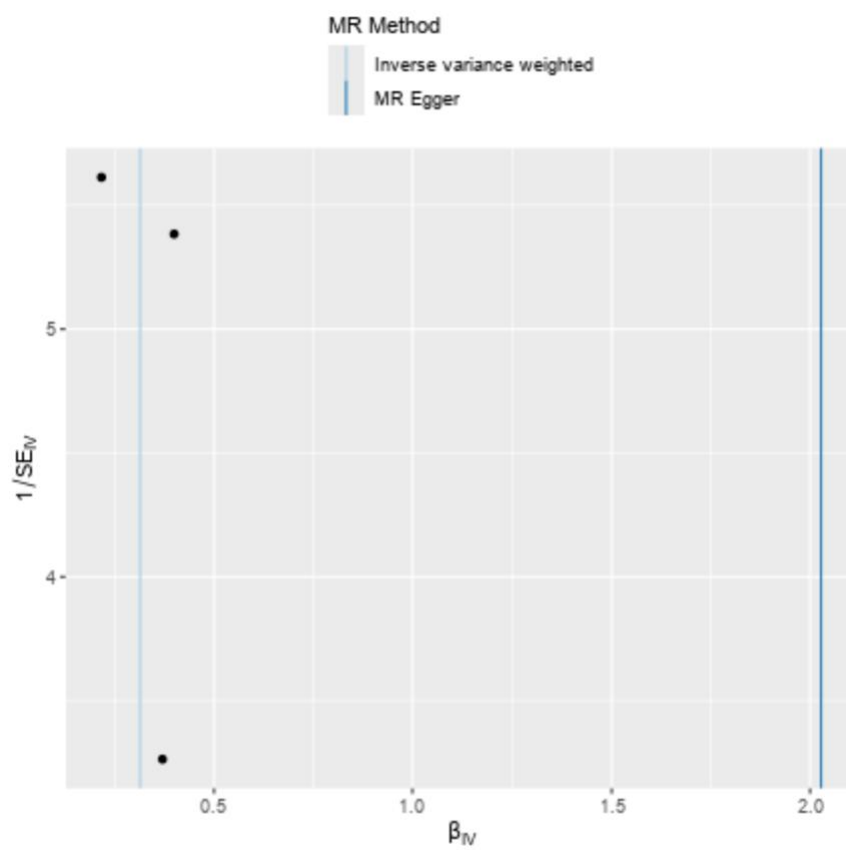

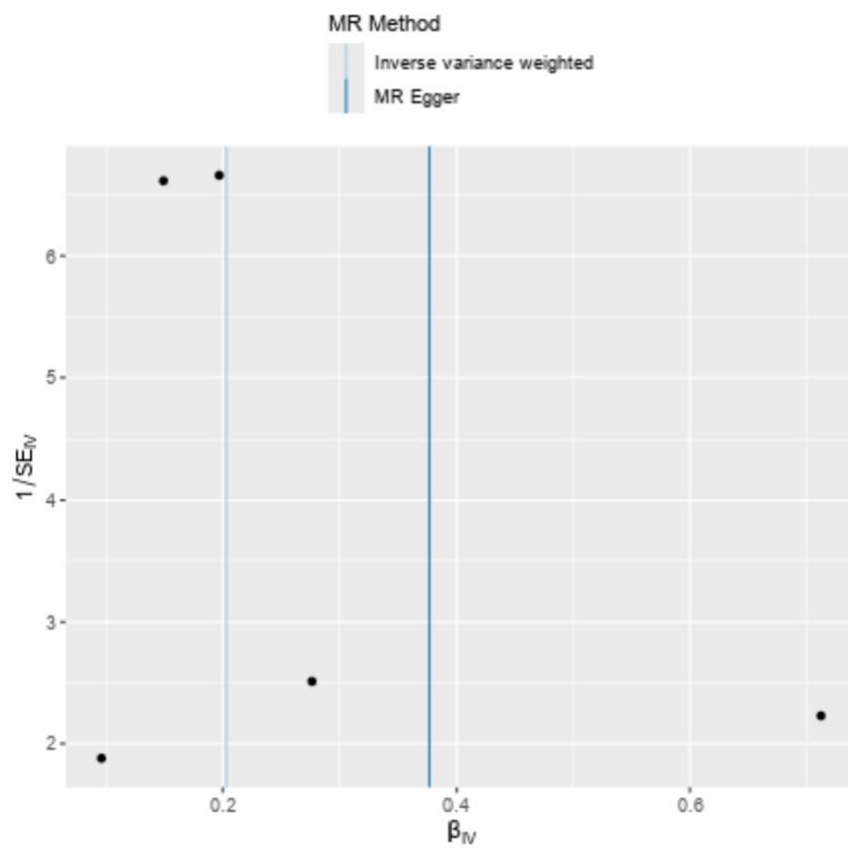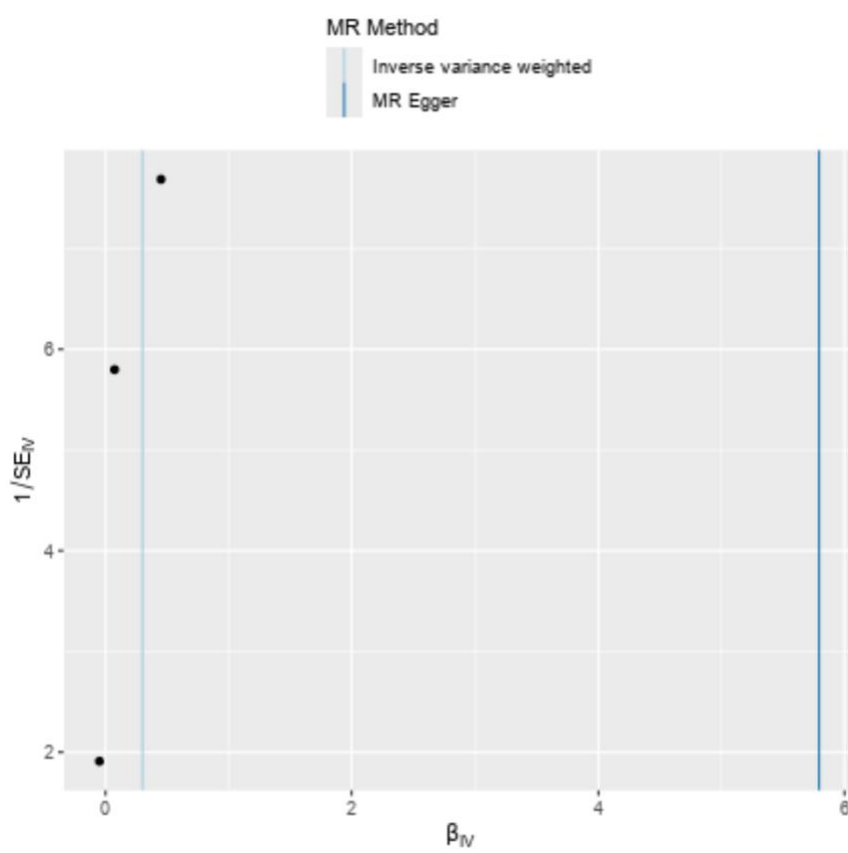

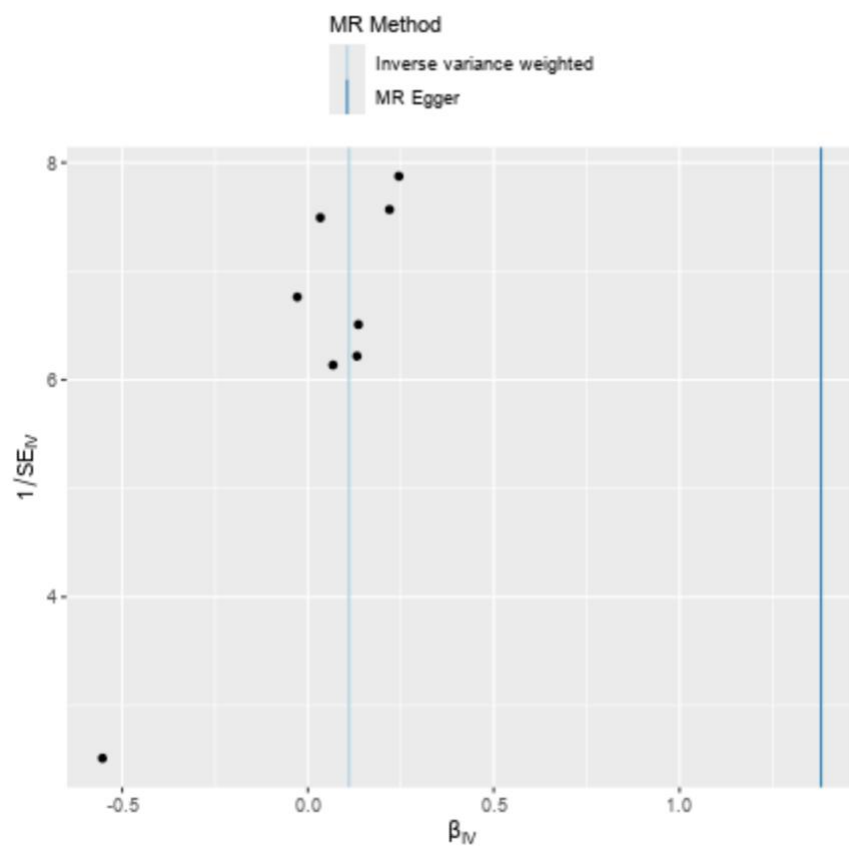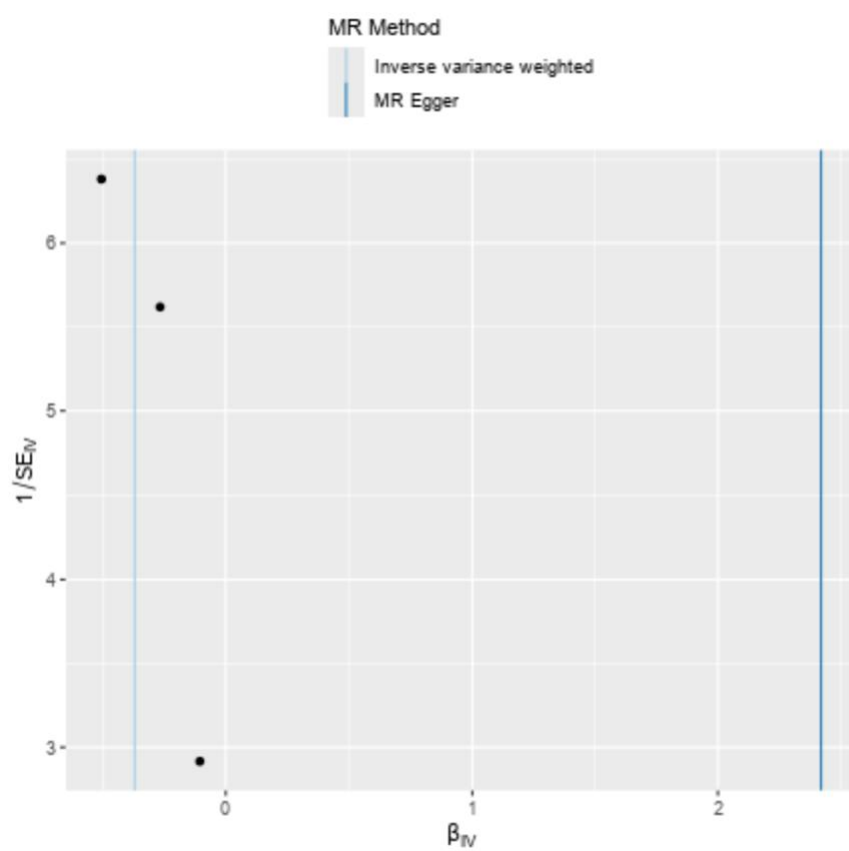

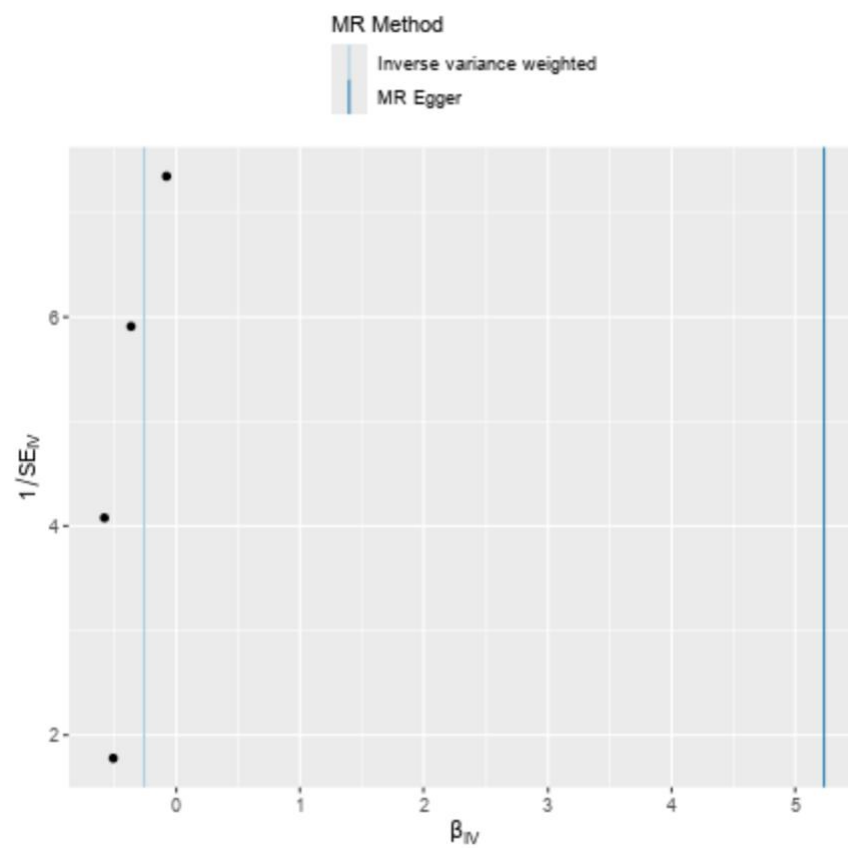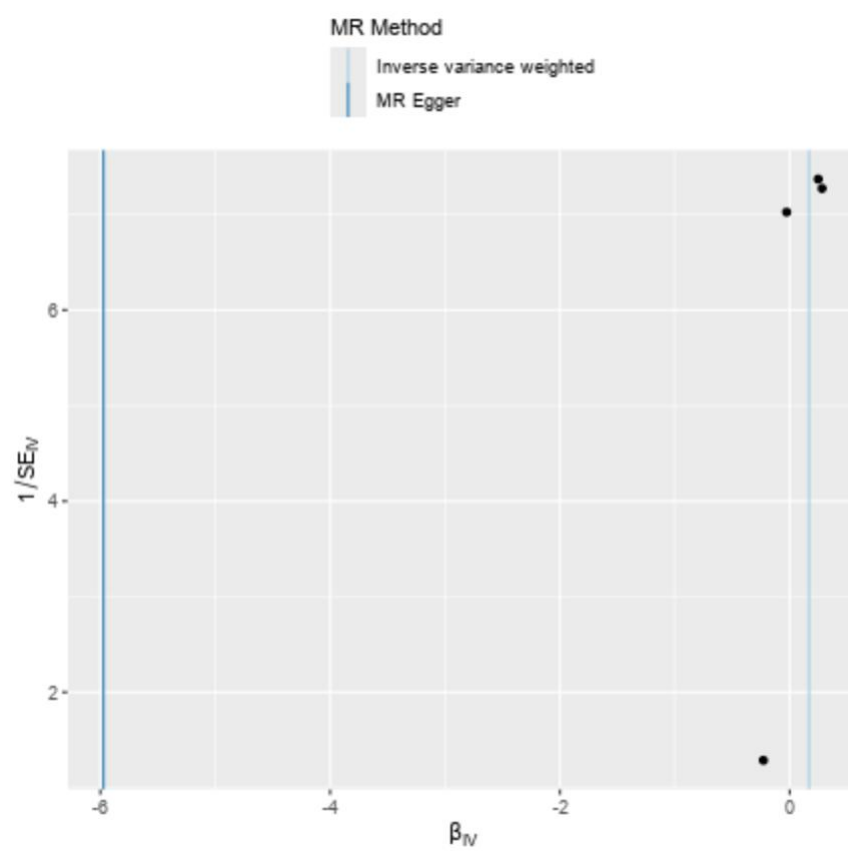

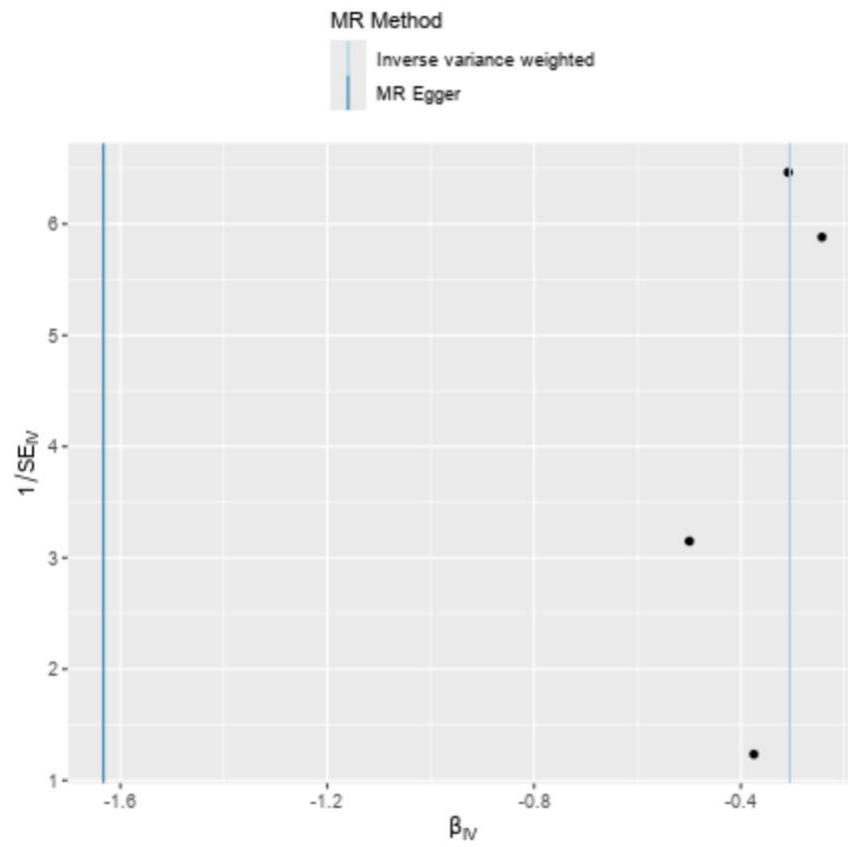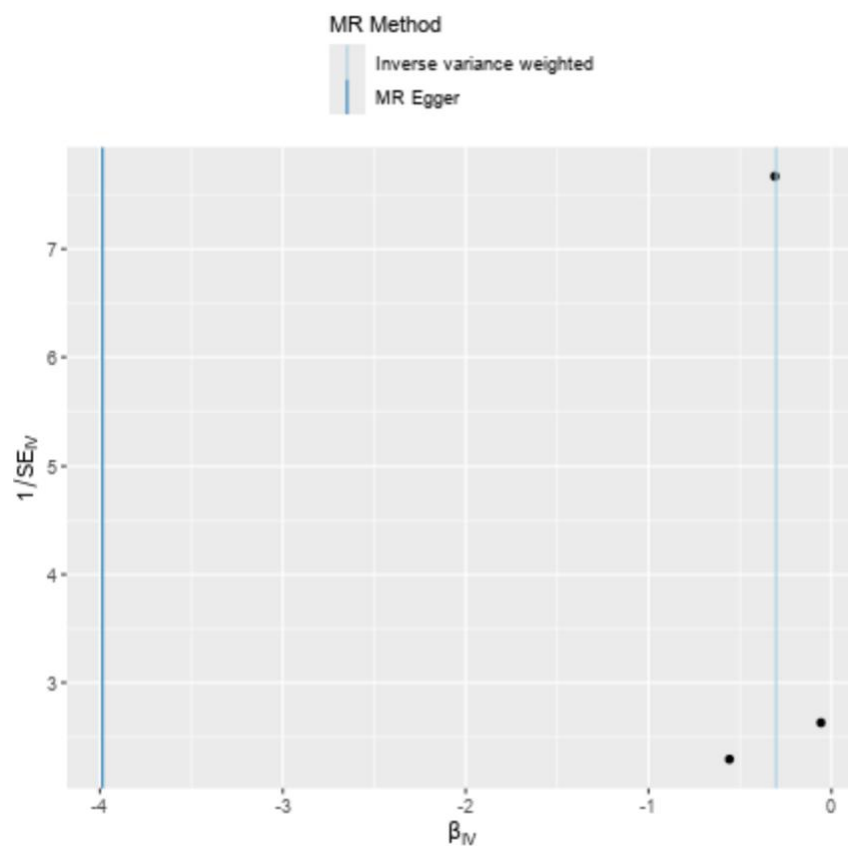

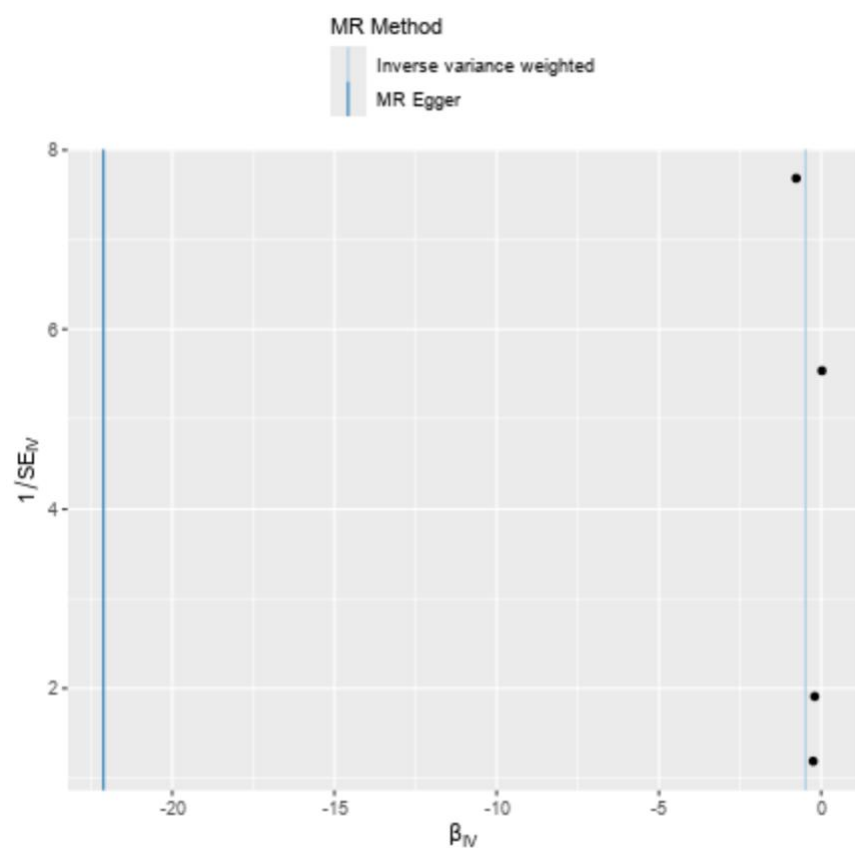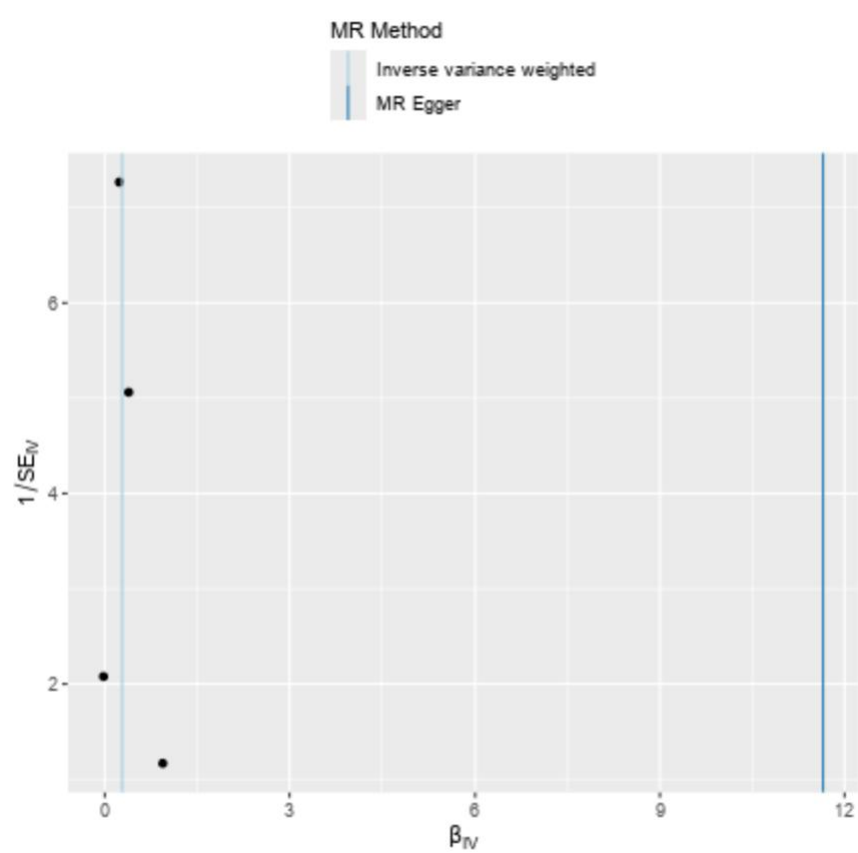

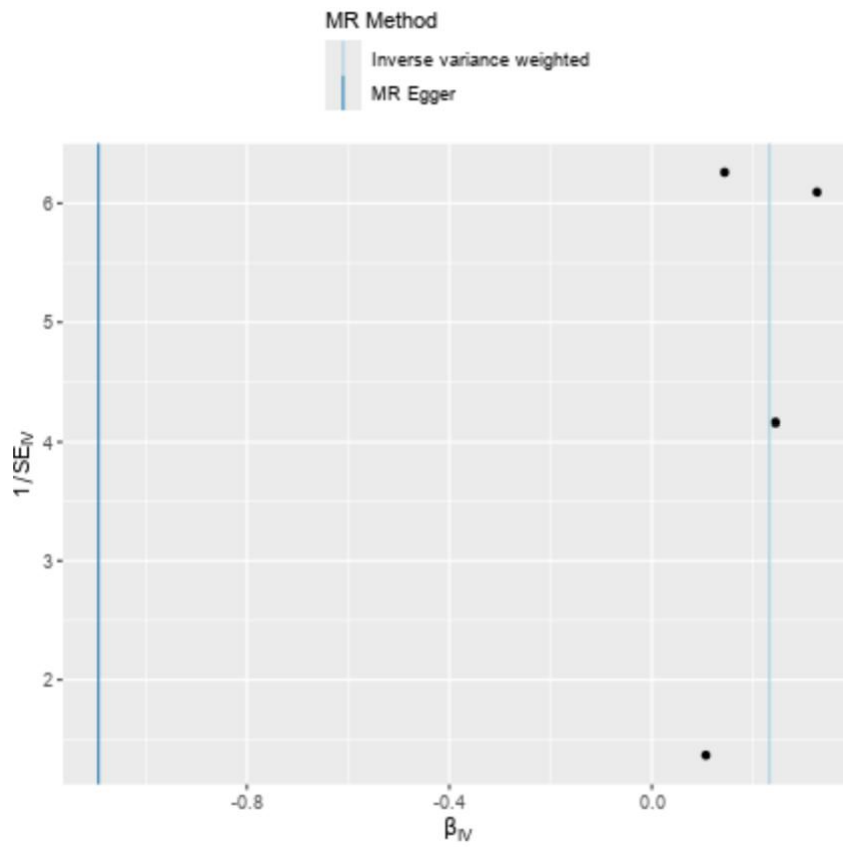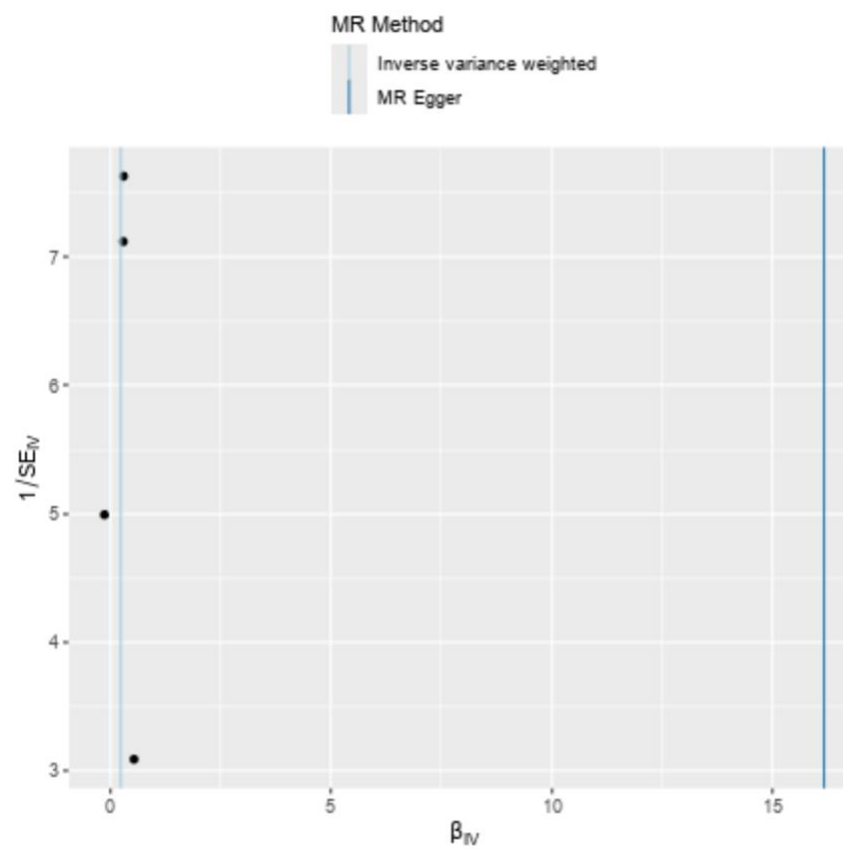

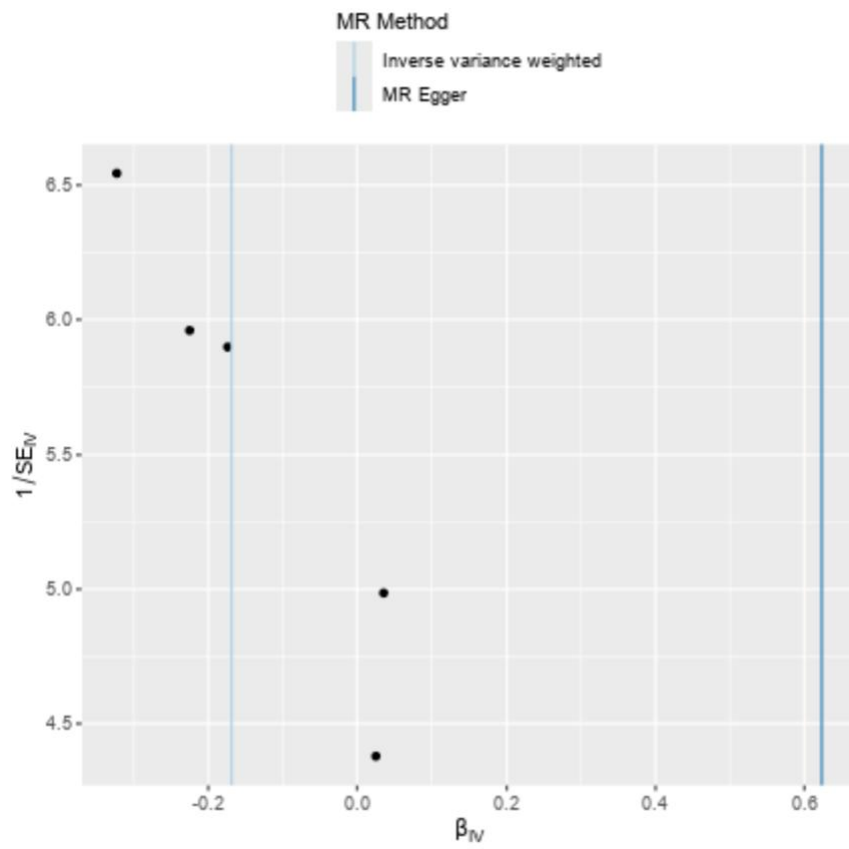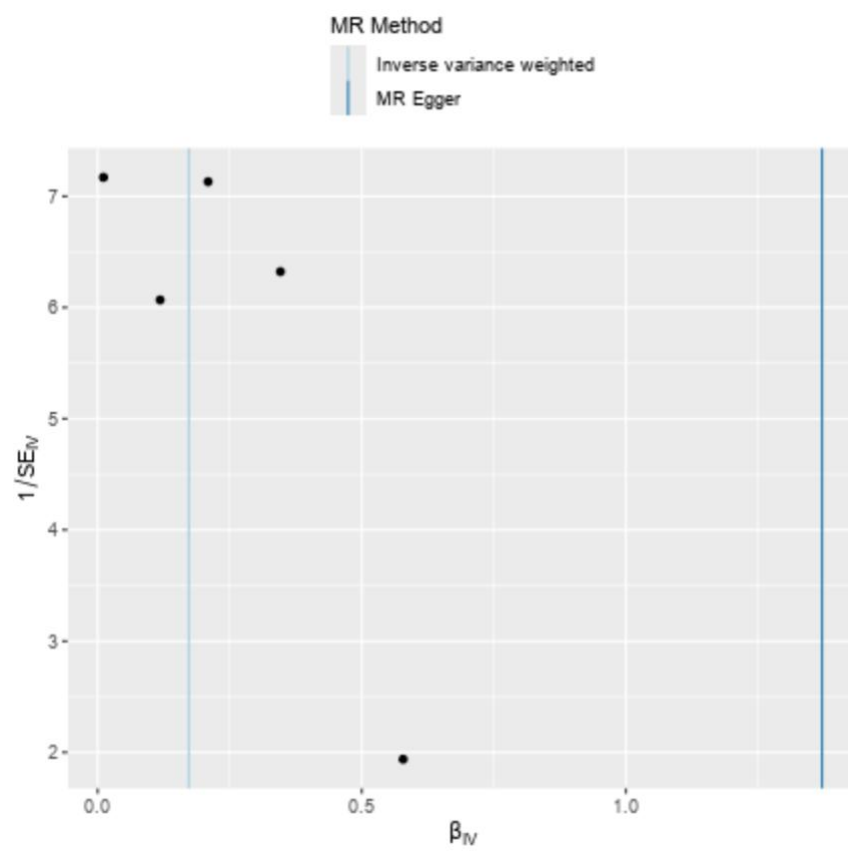

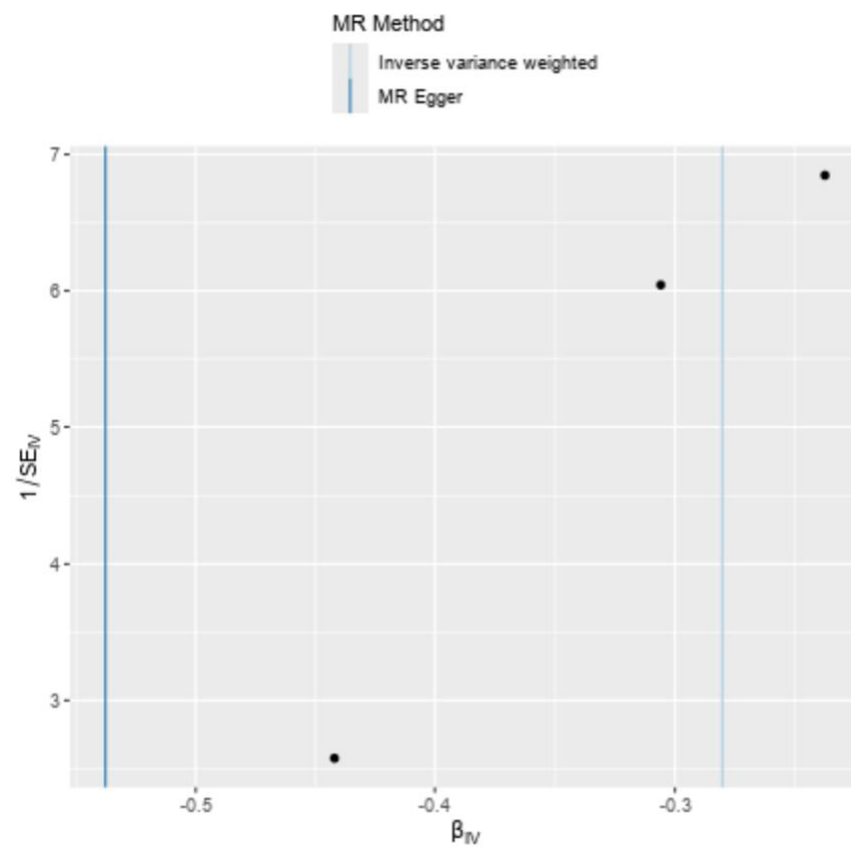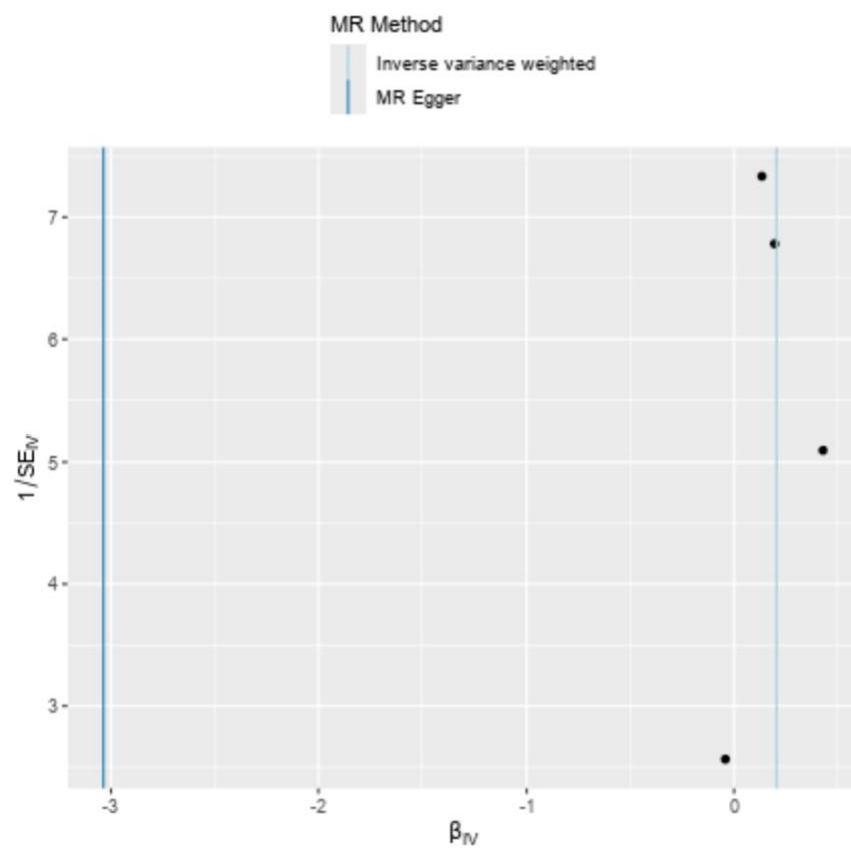

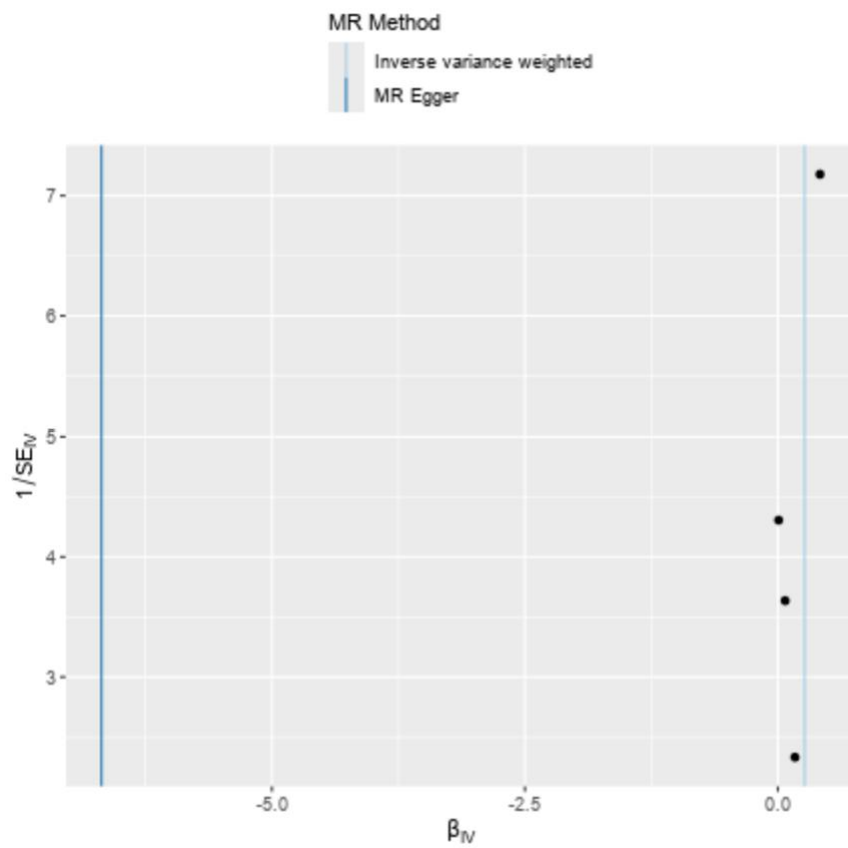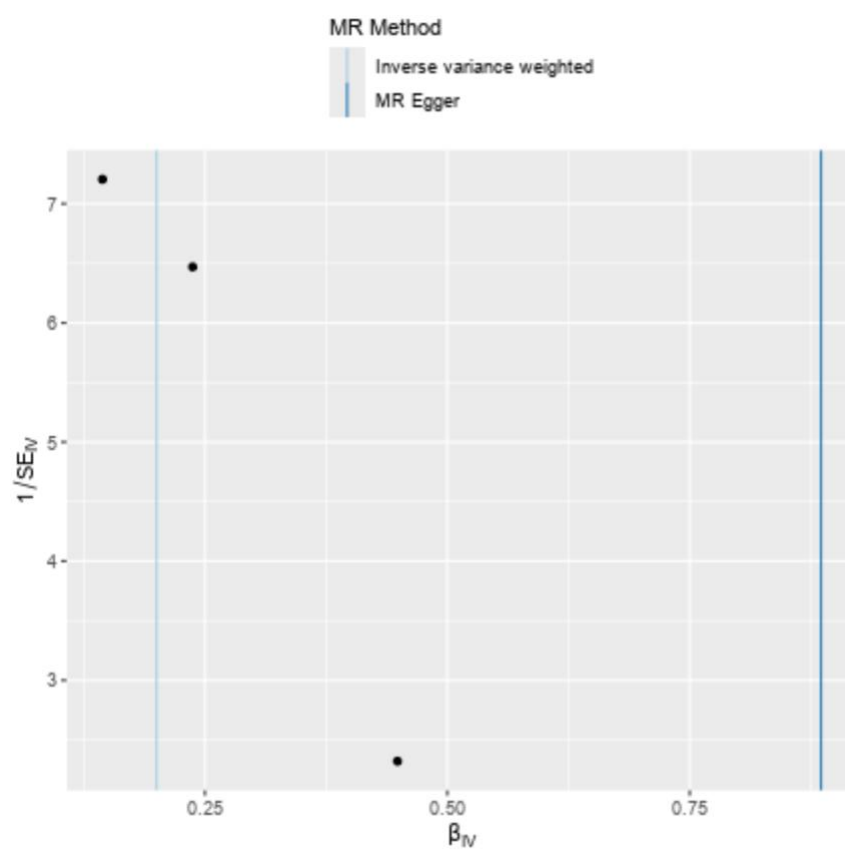

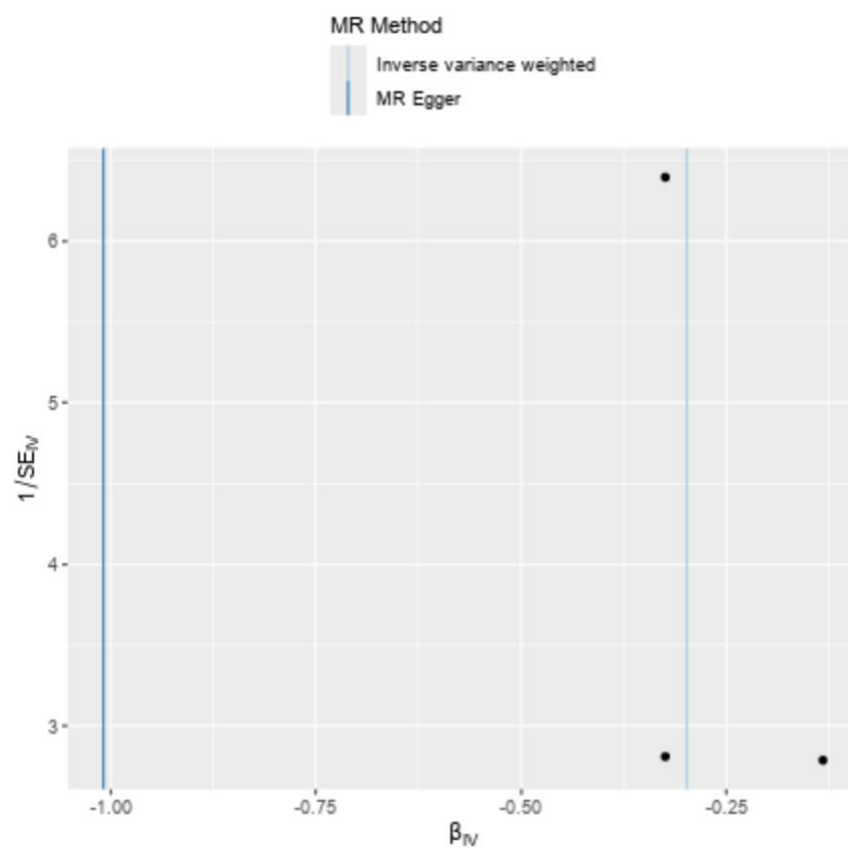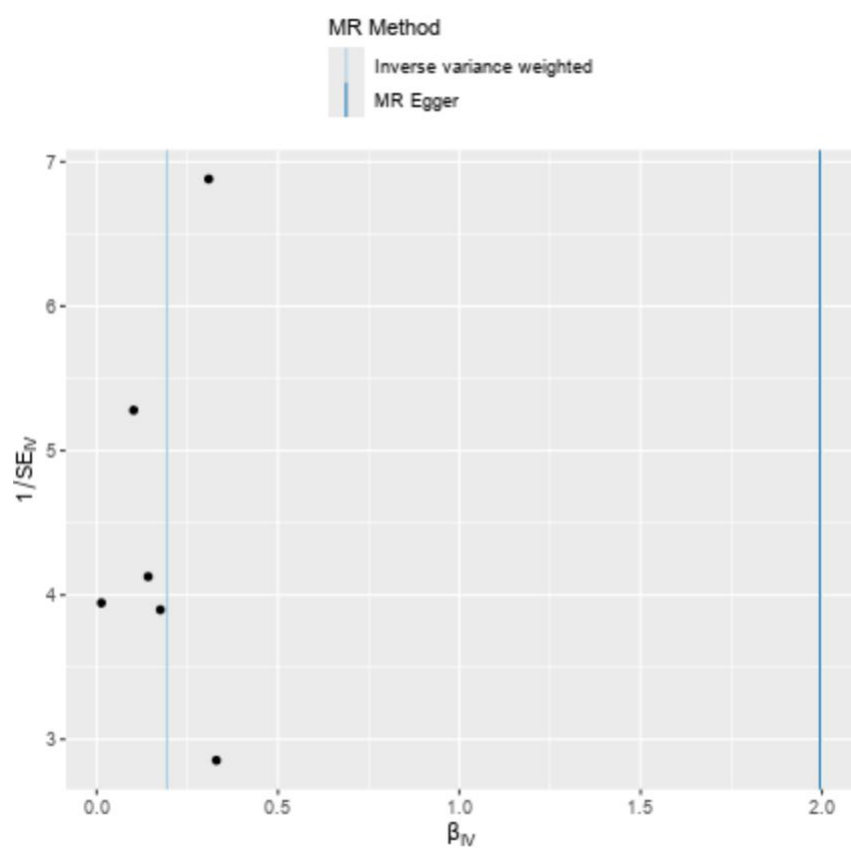

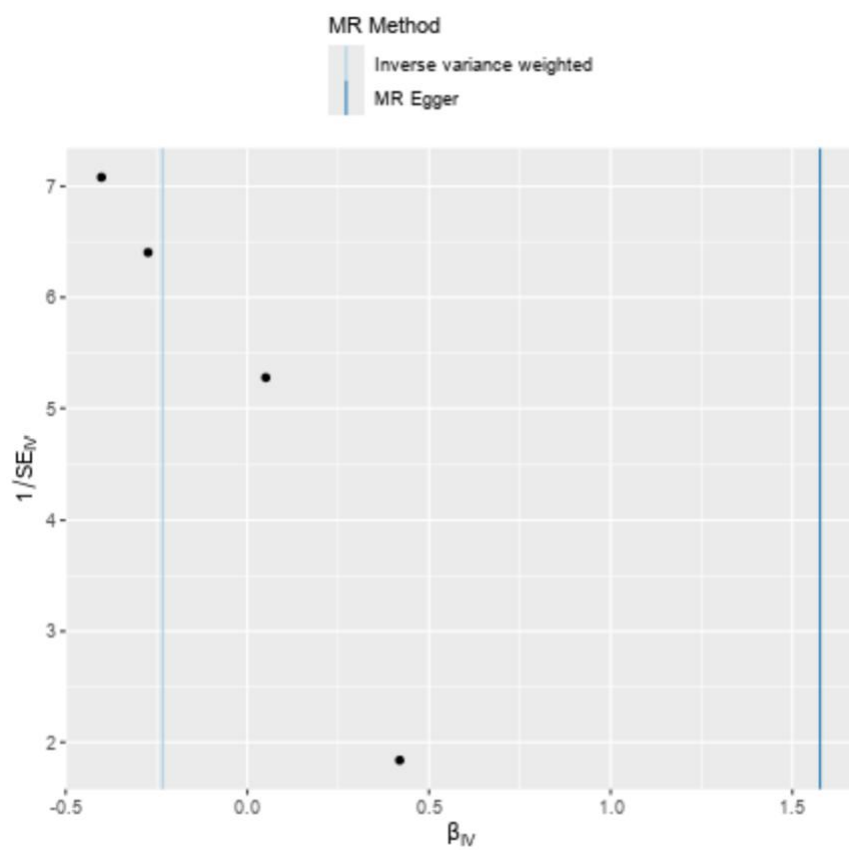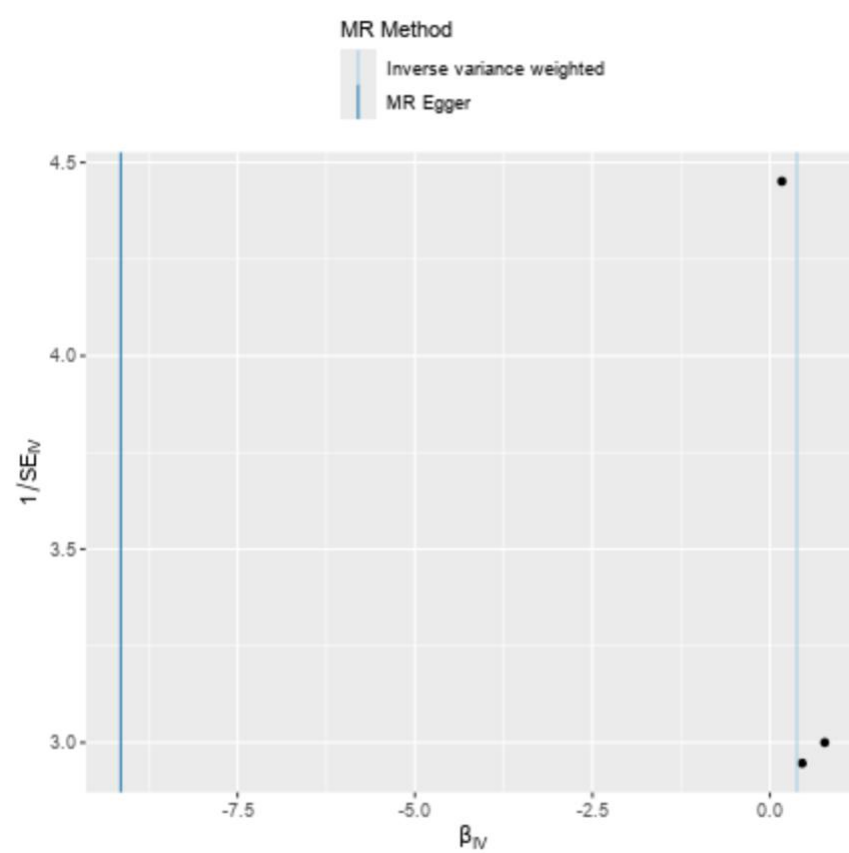

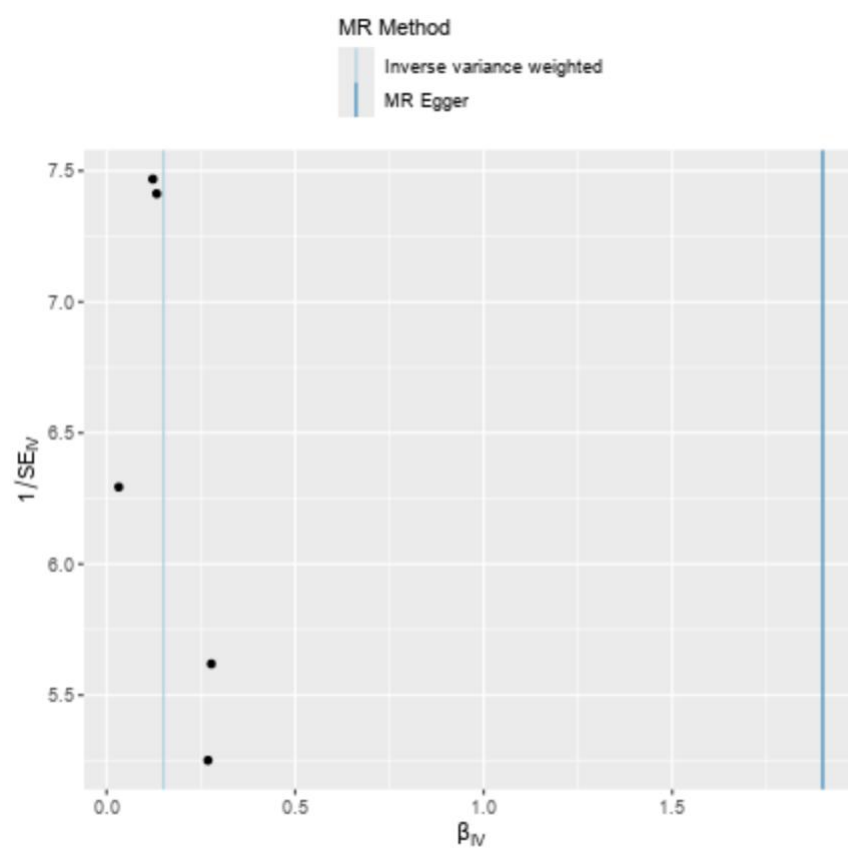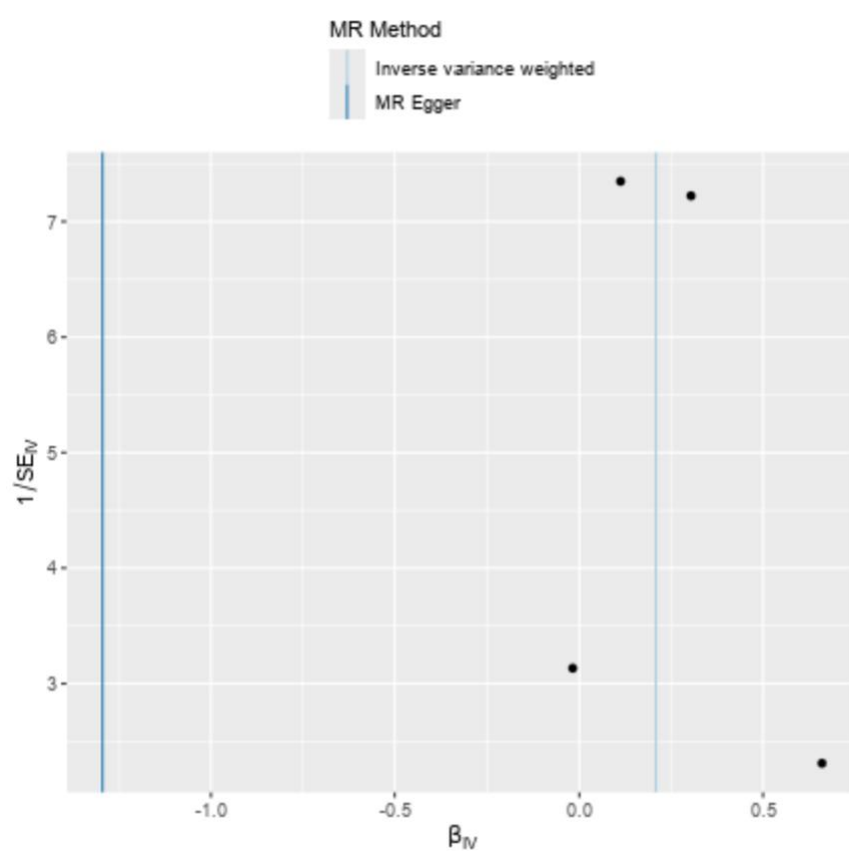

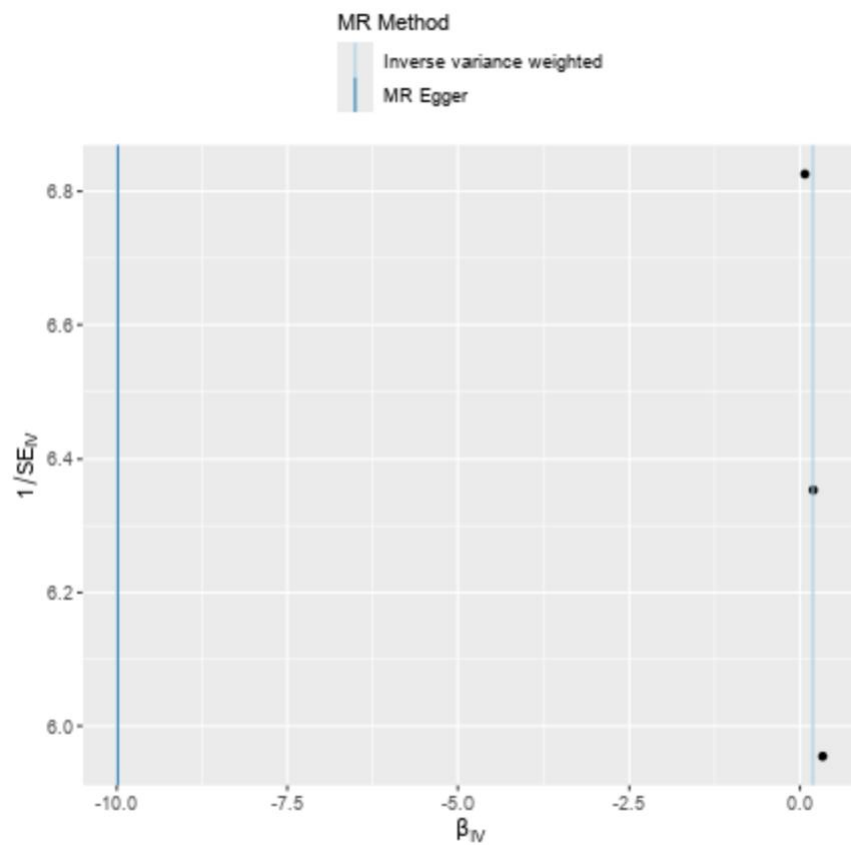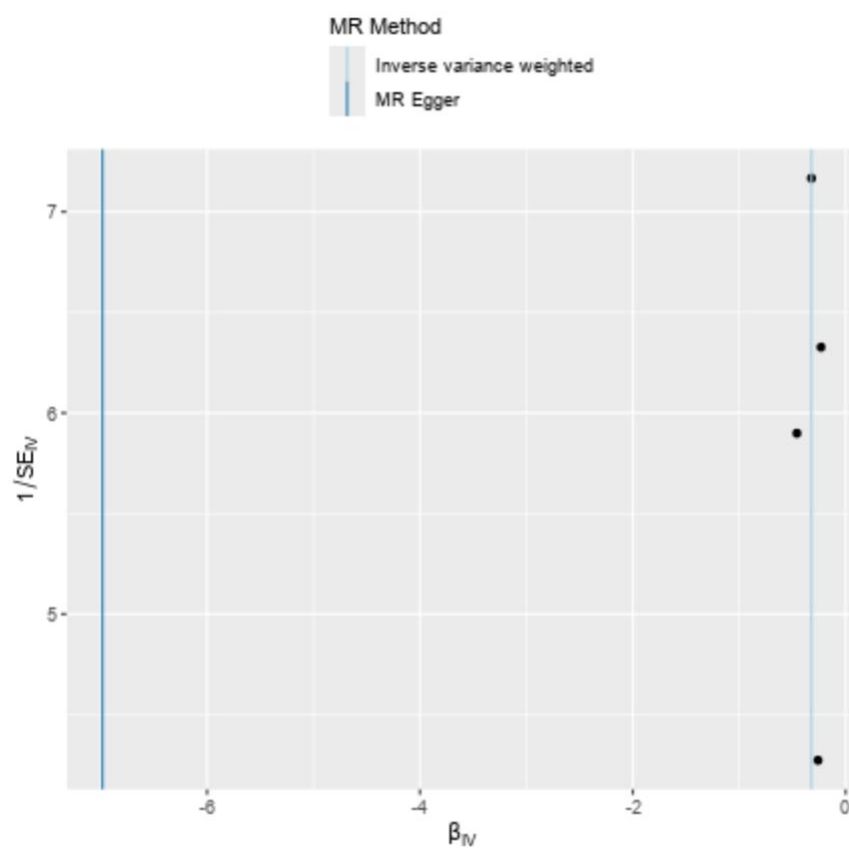

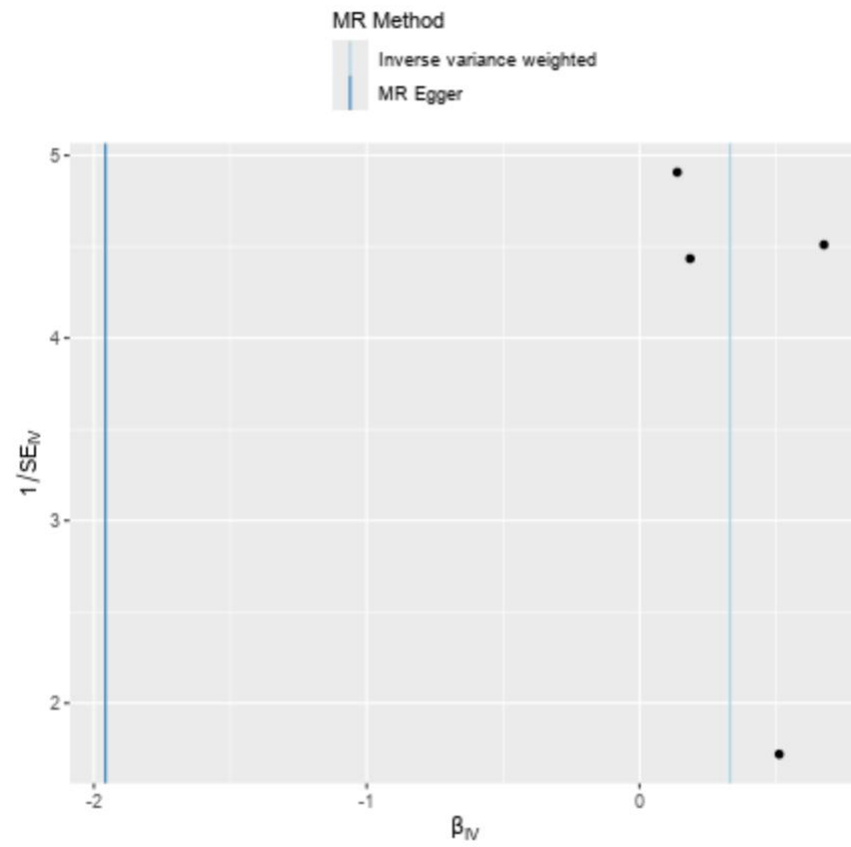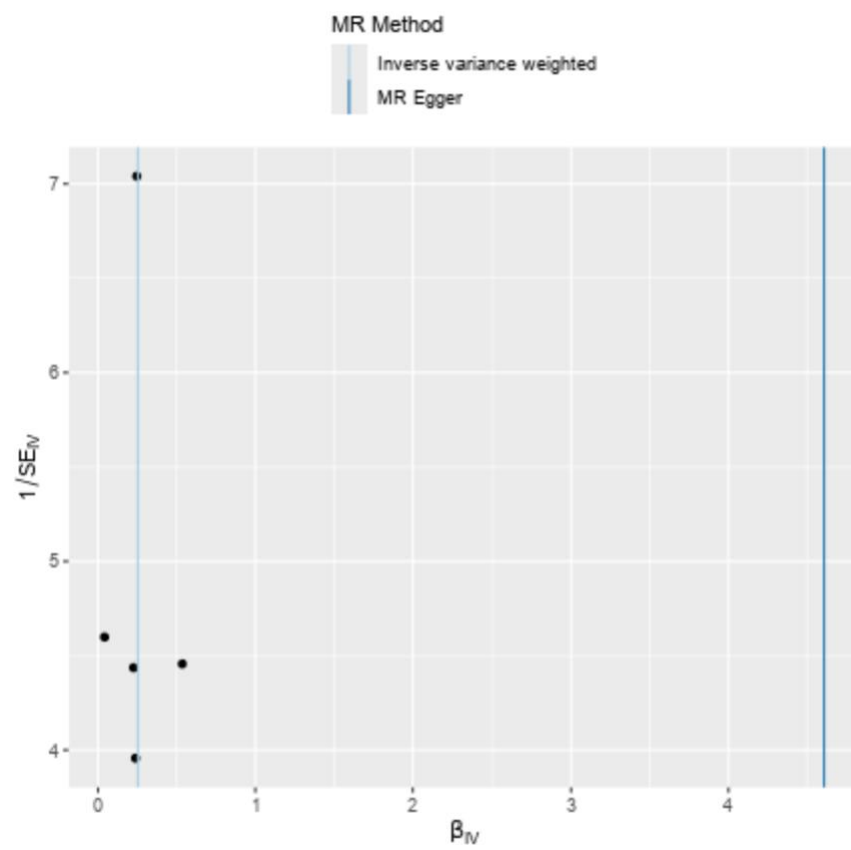

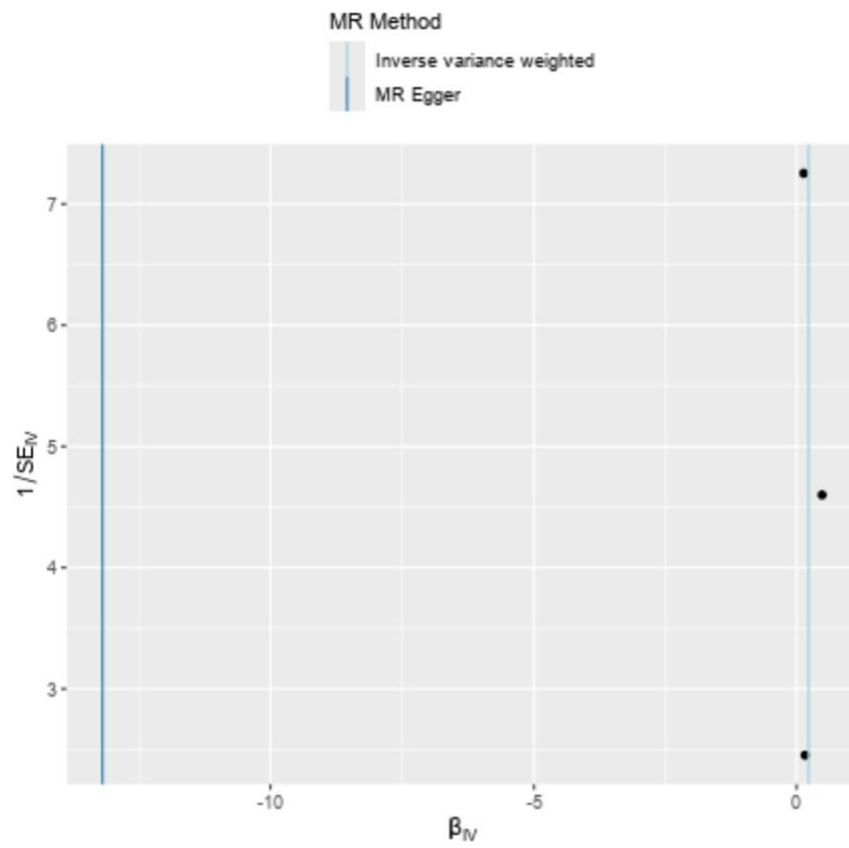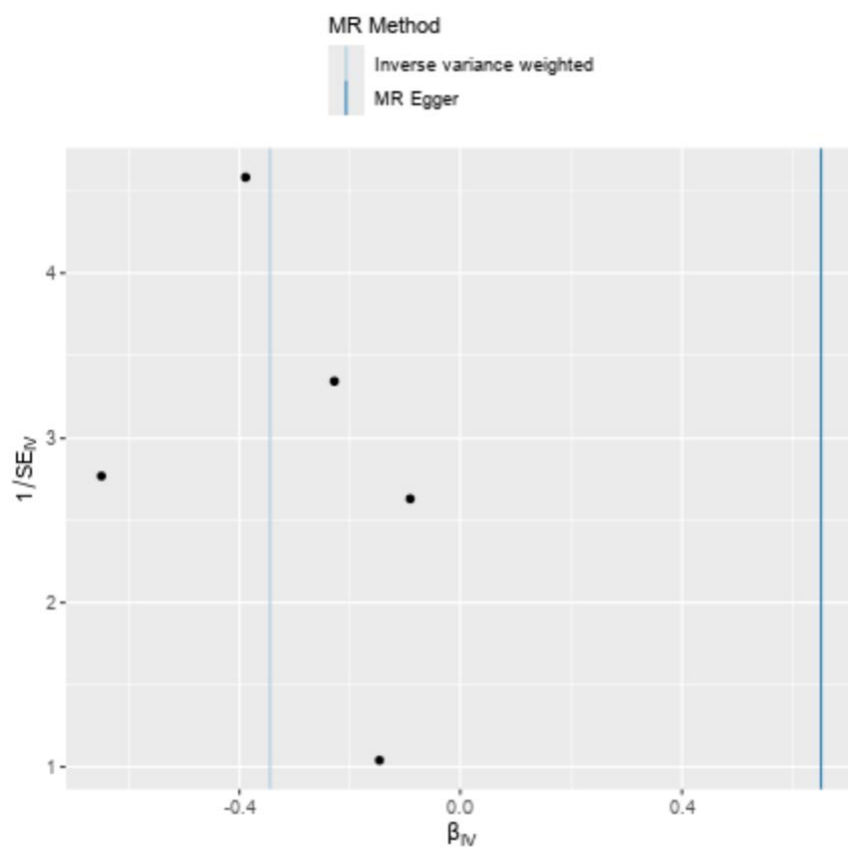

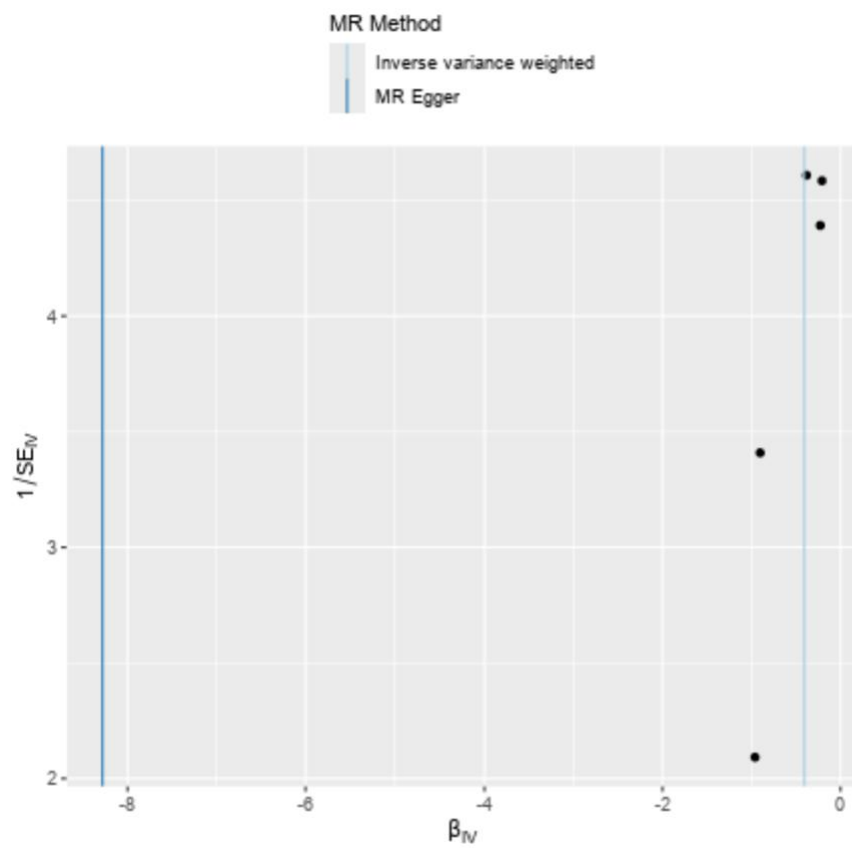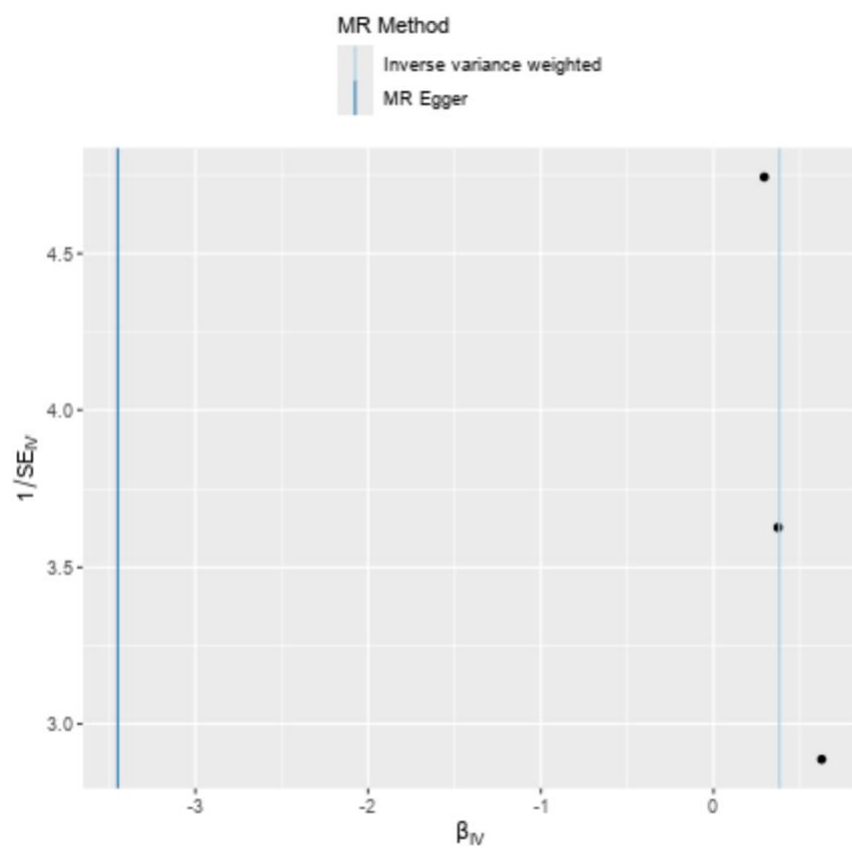

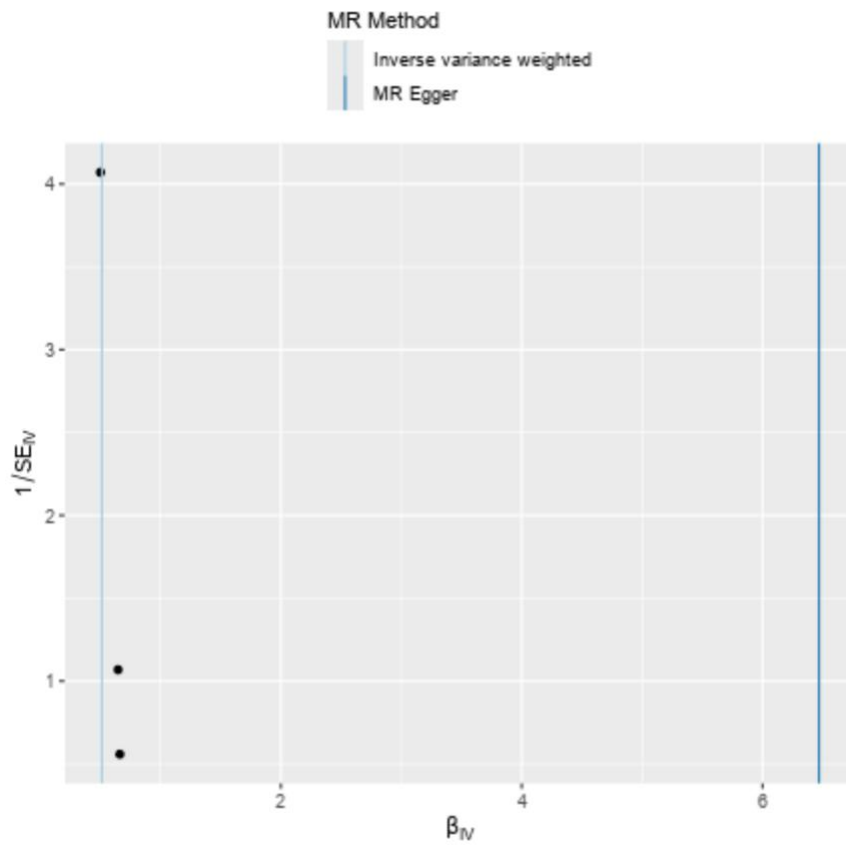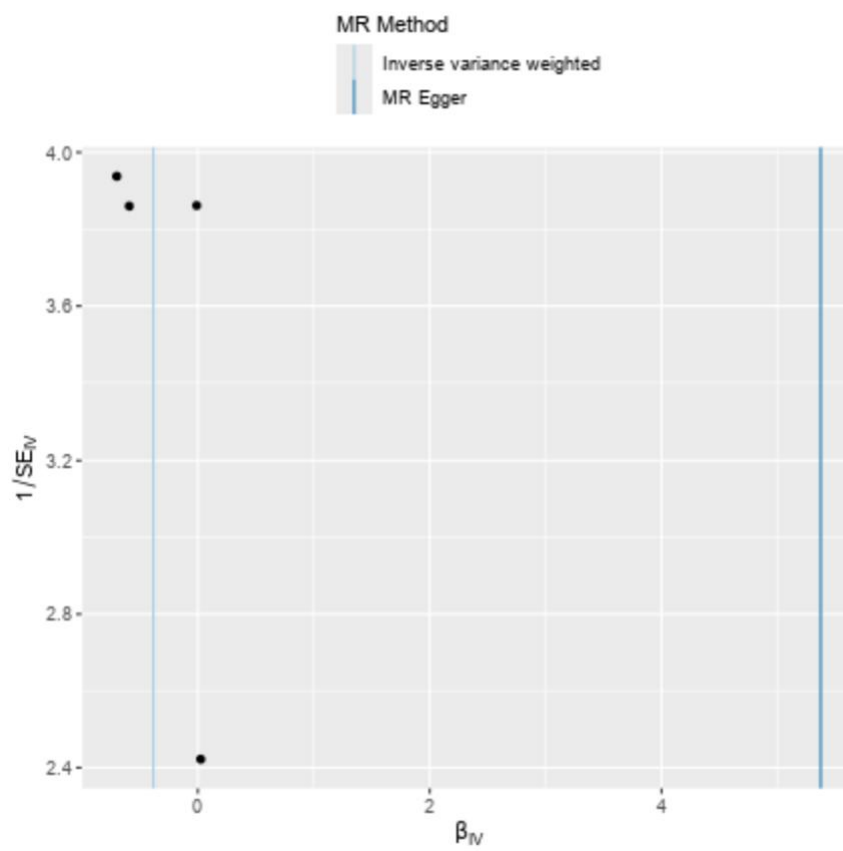

Supplement: Supplementary file 5 — Supplemnetary 5 [file BRB3-15-e70753-s002.pdf]

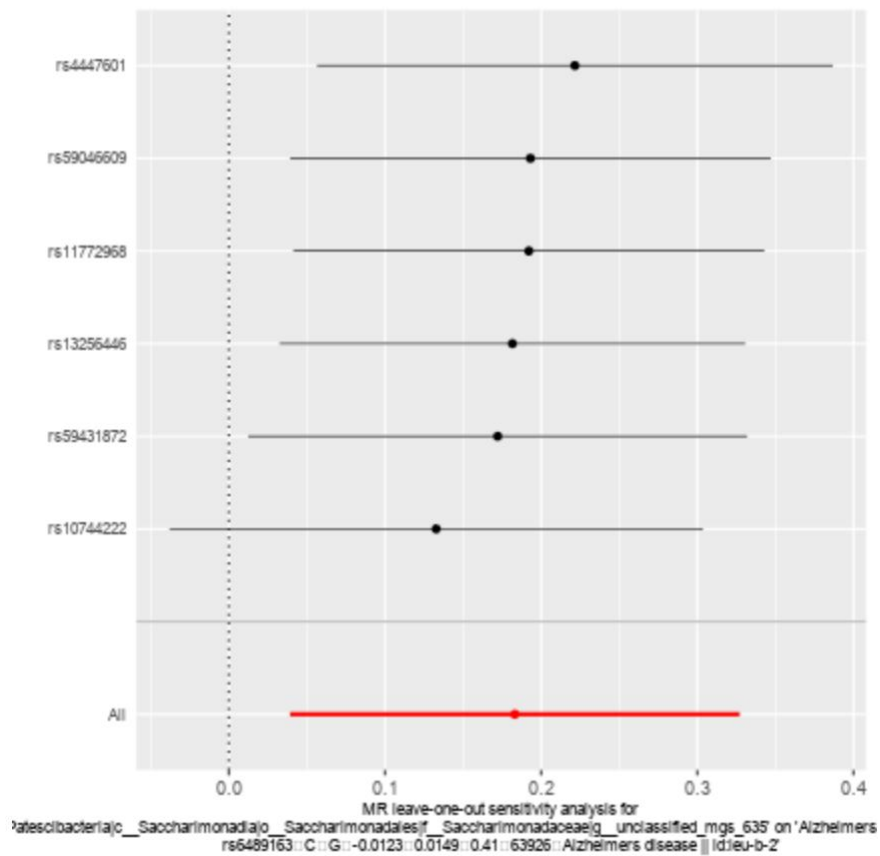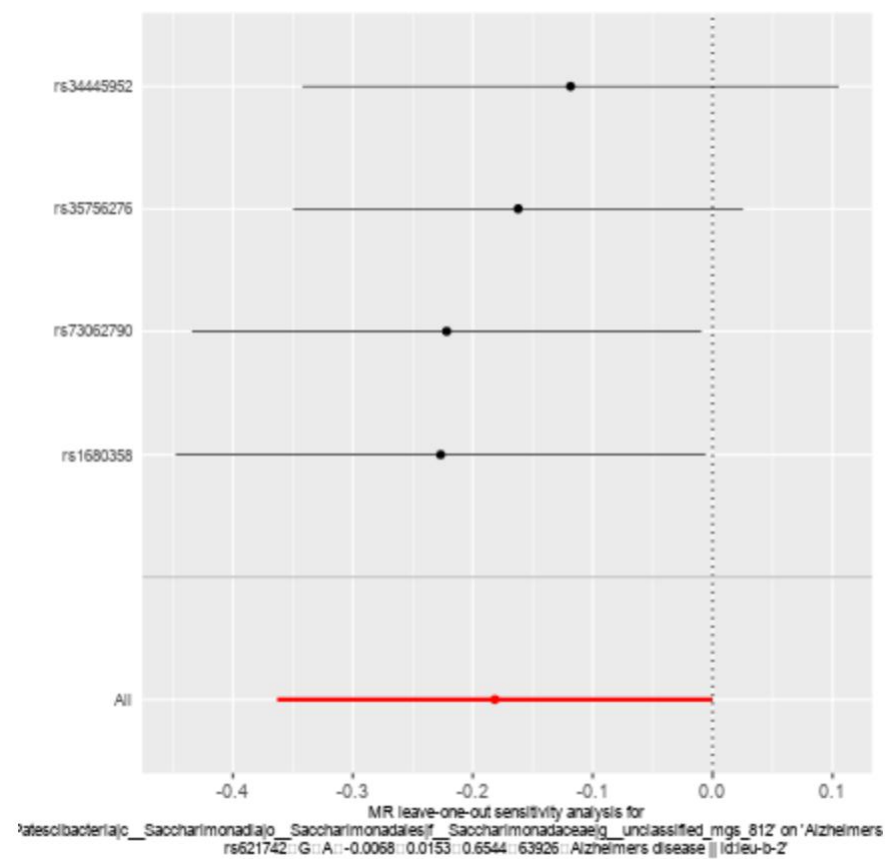

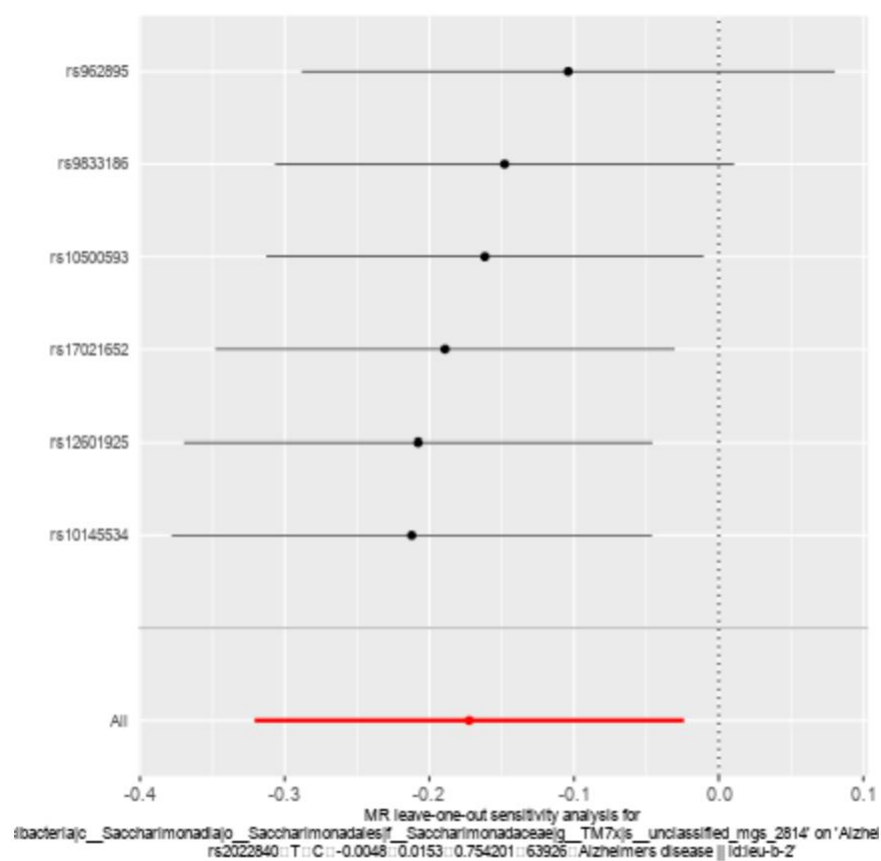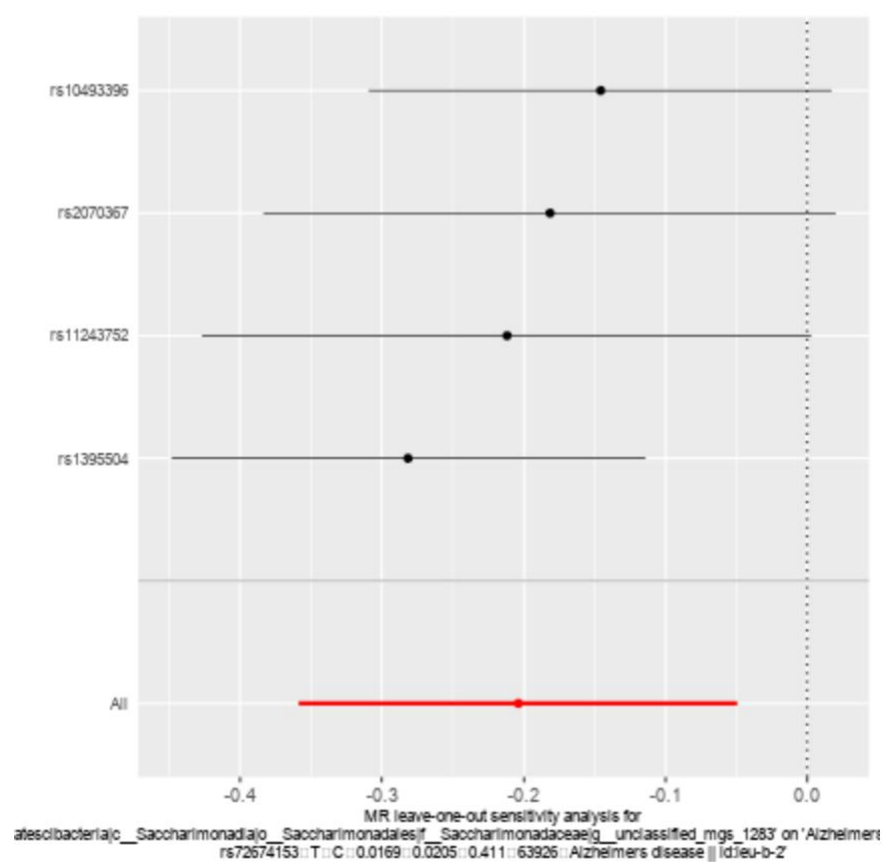

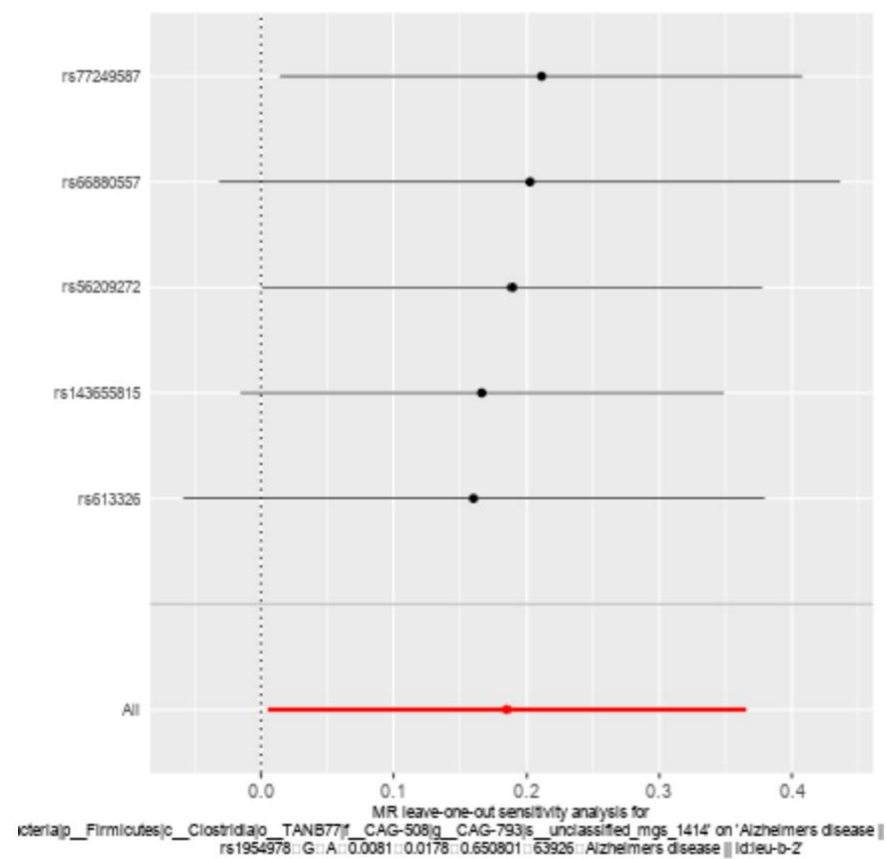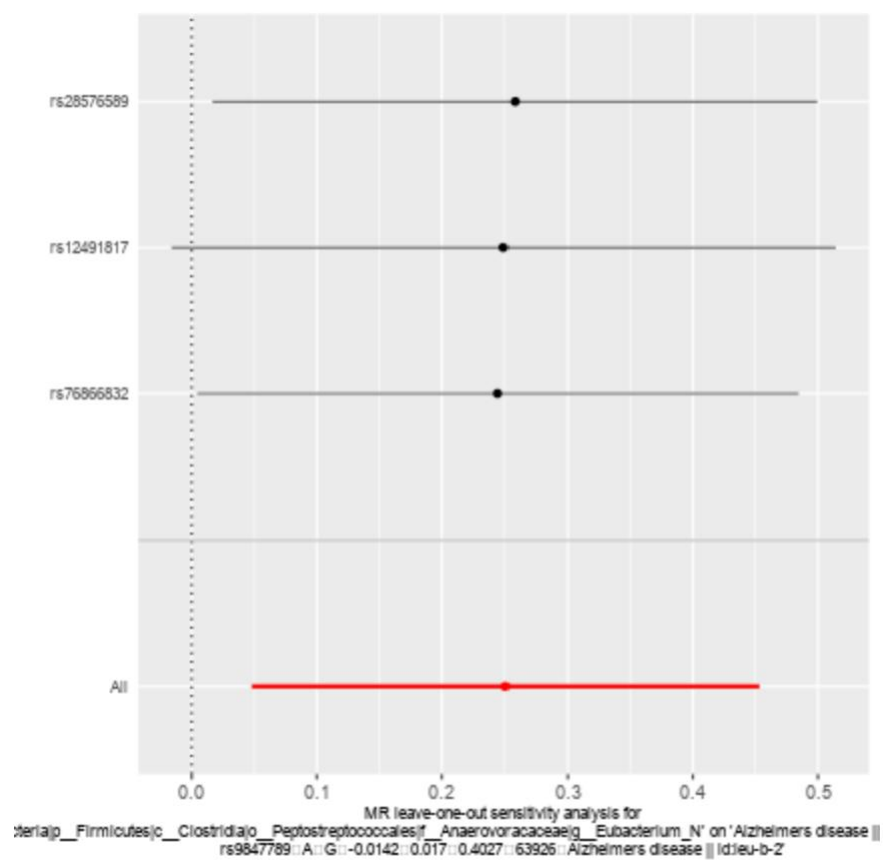

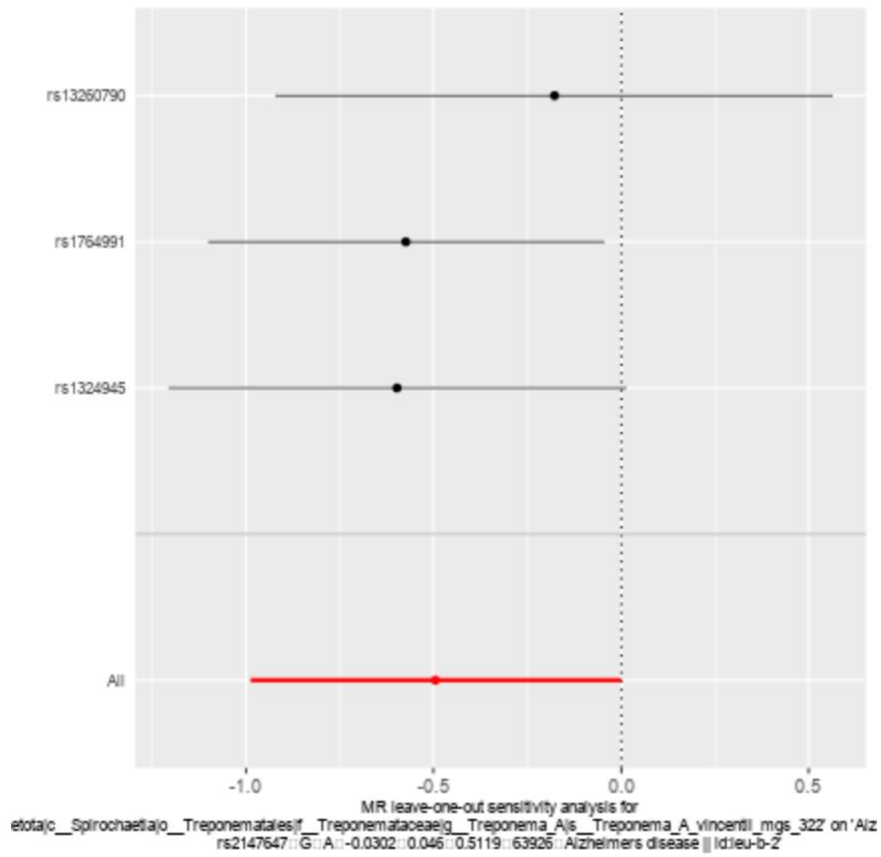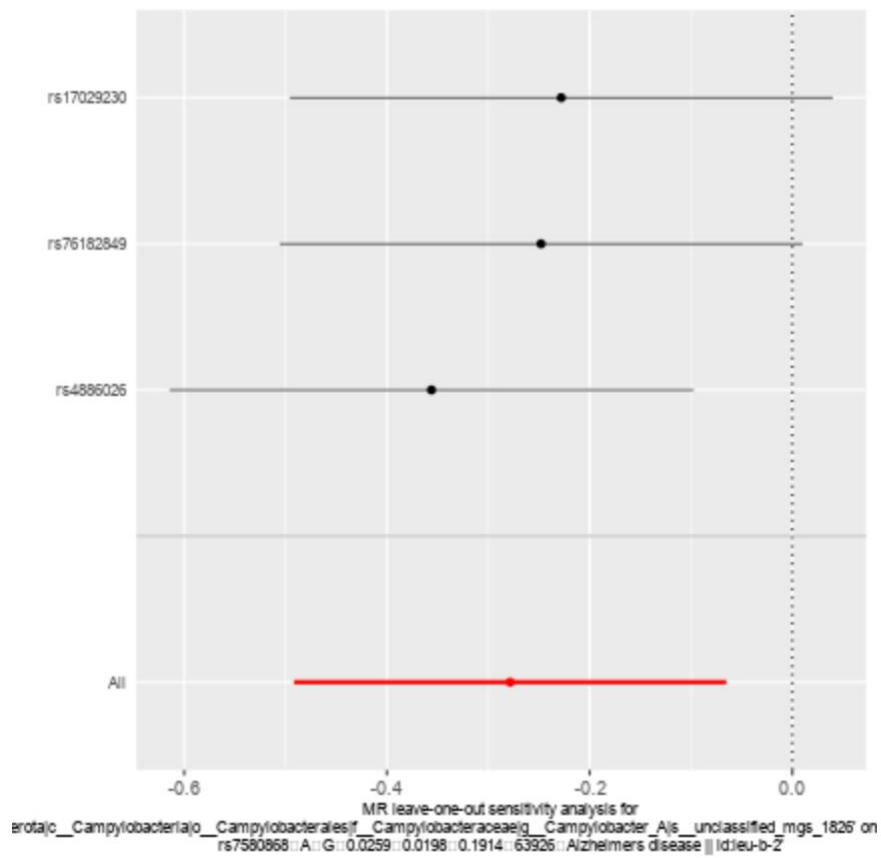

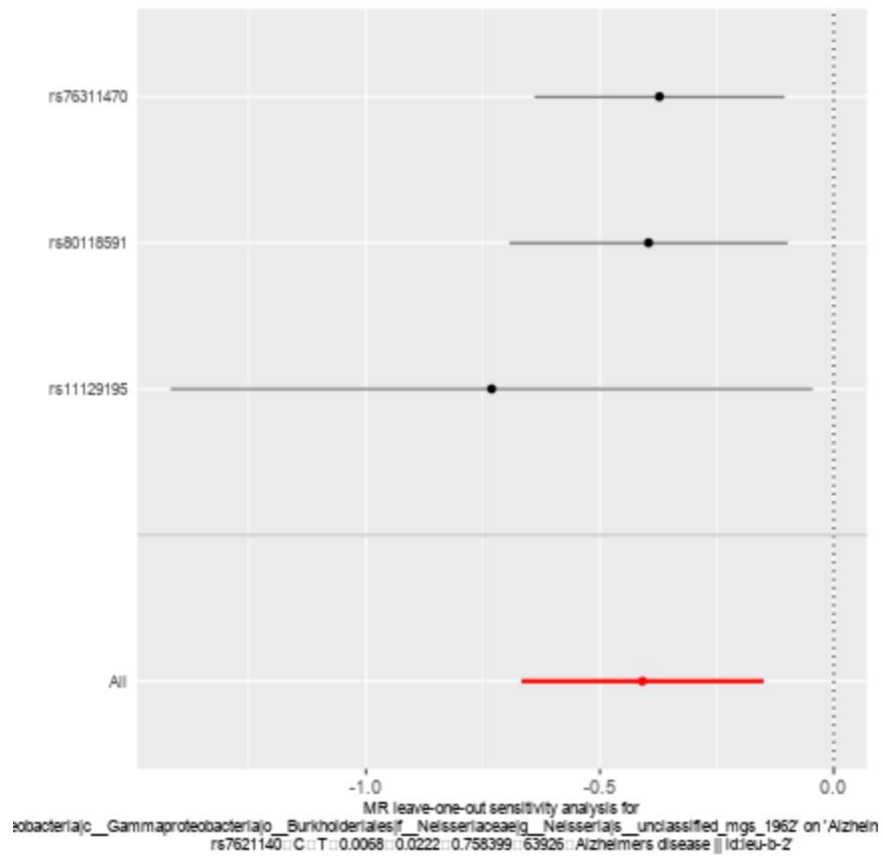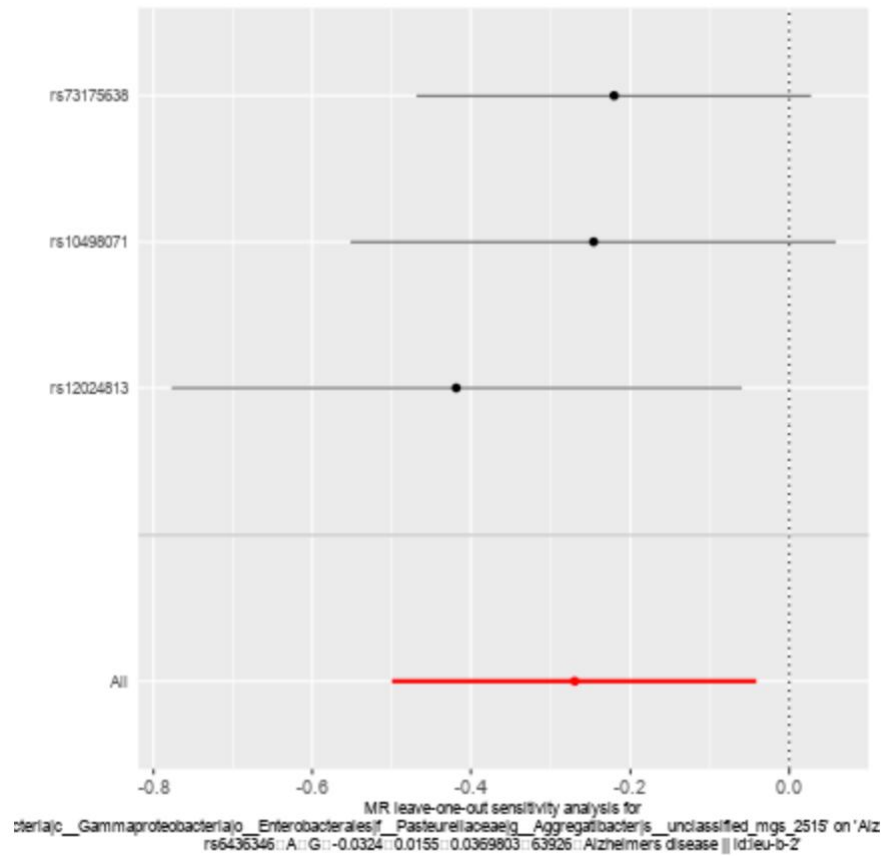

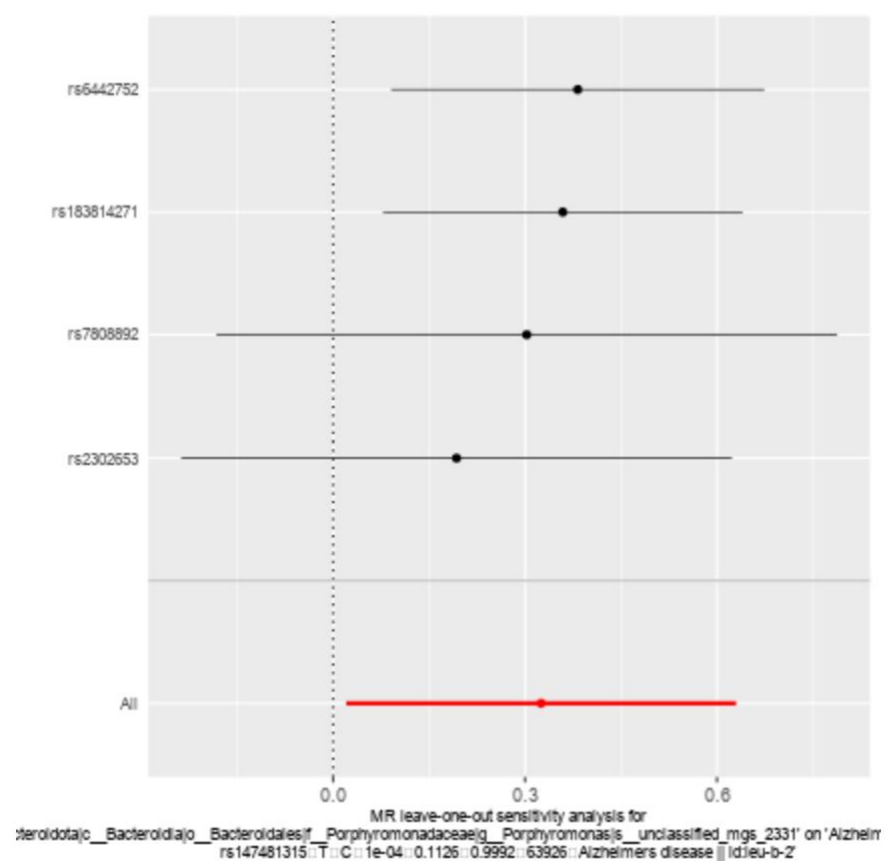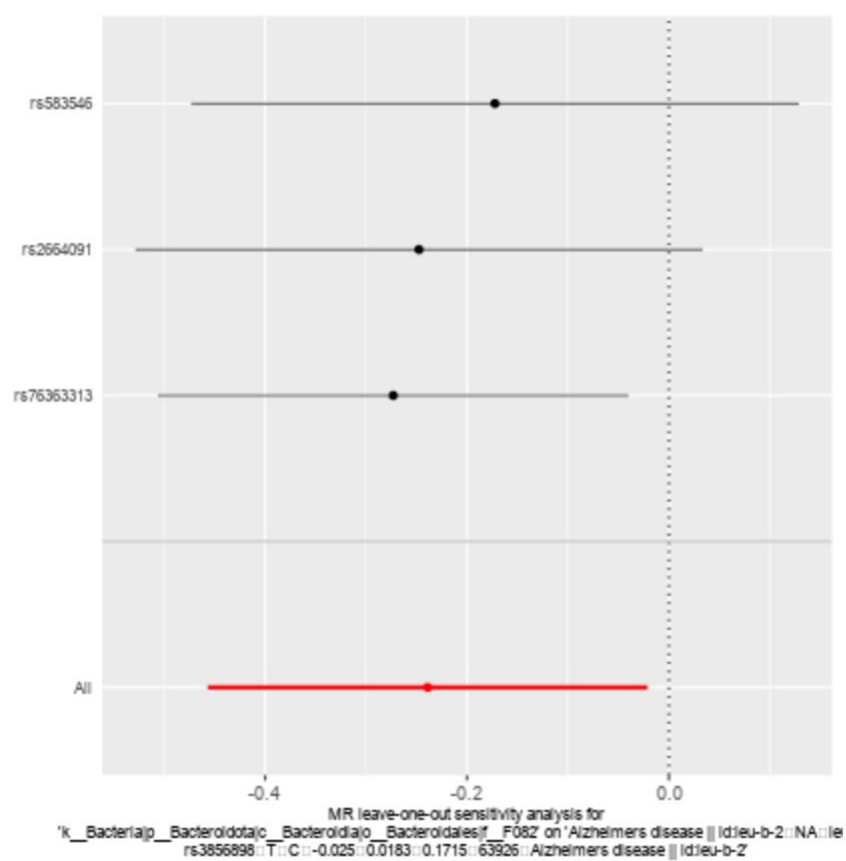

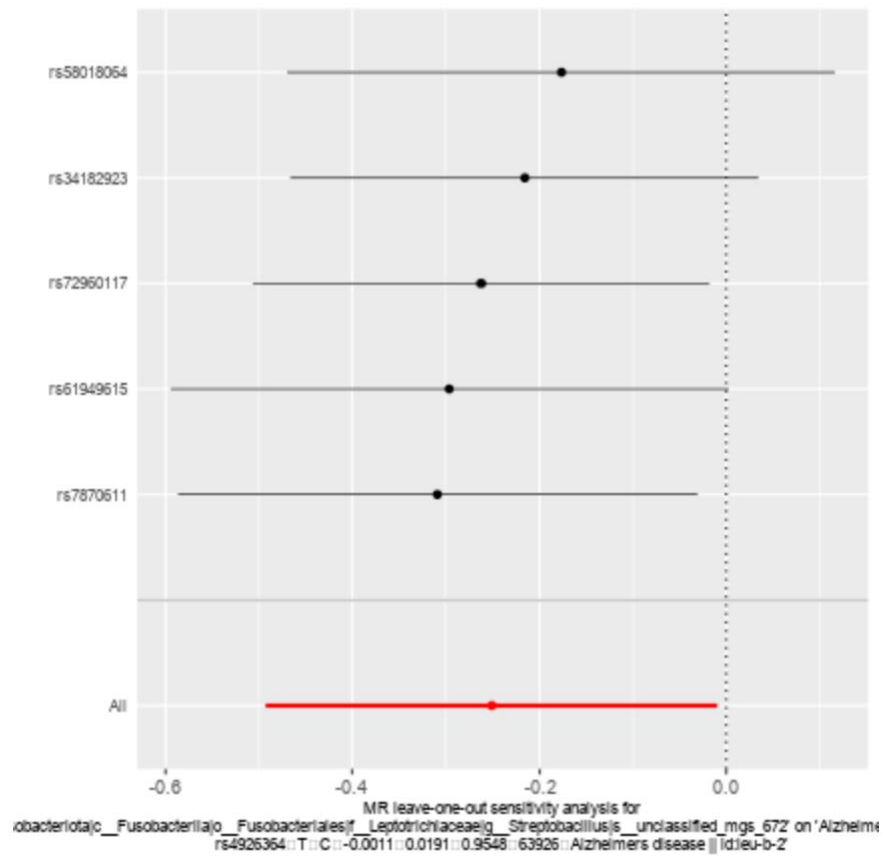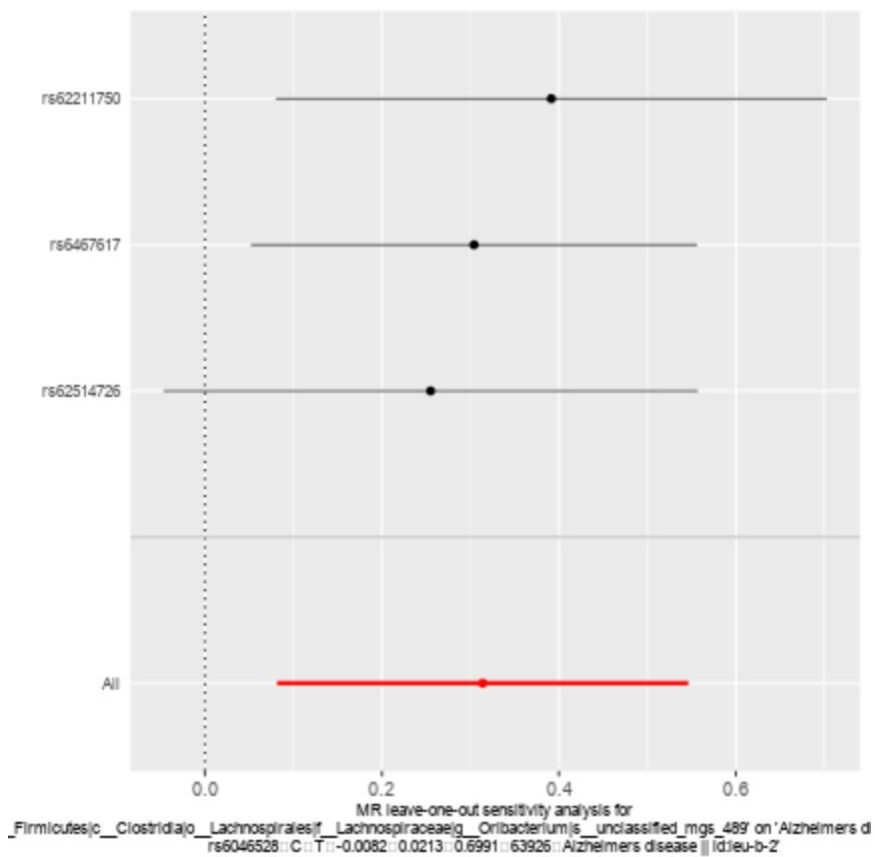

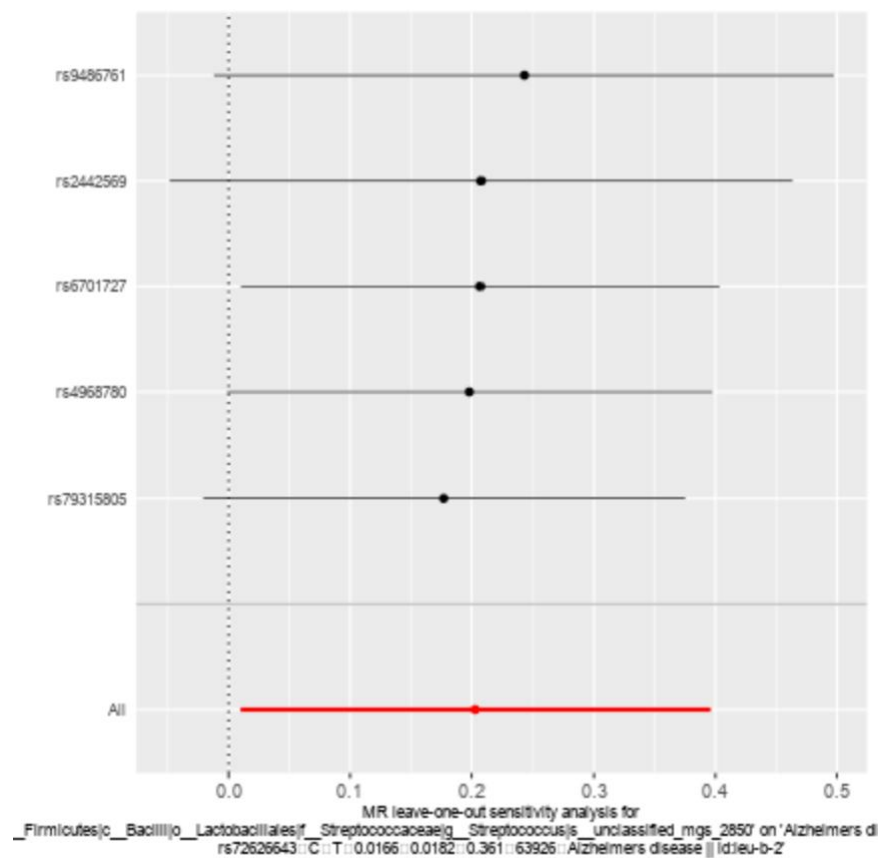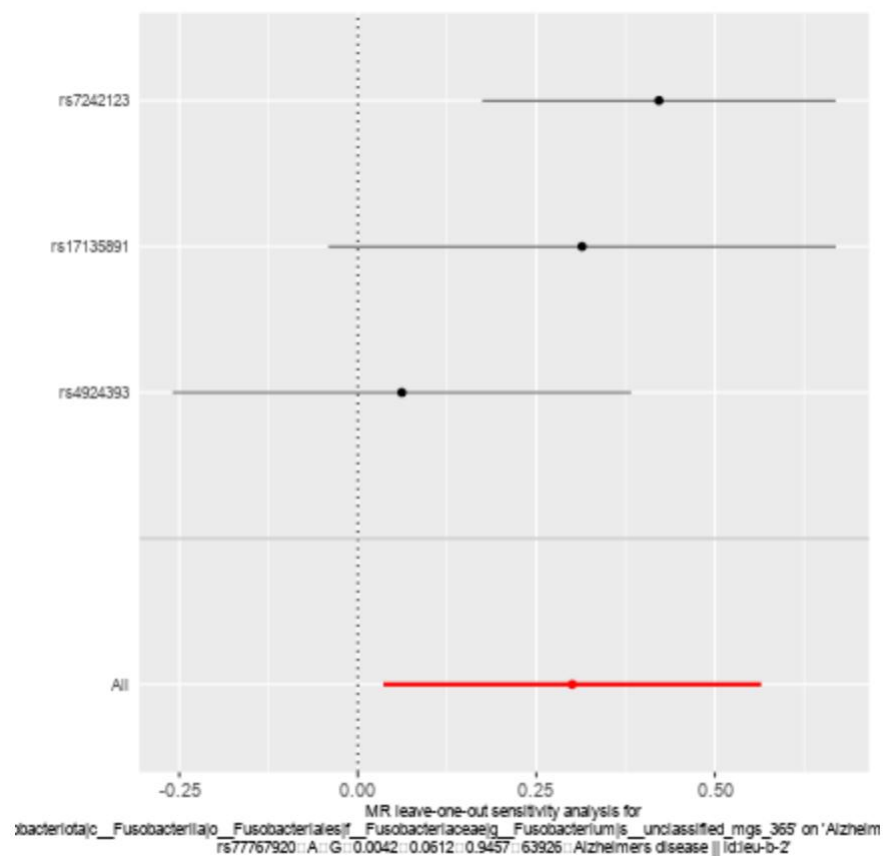

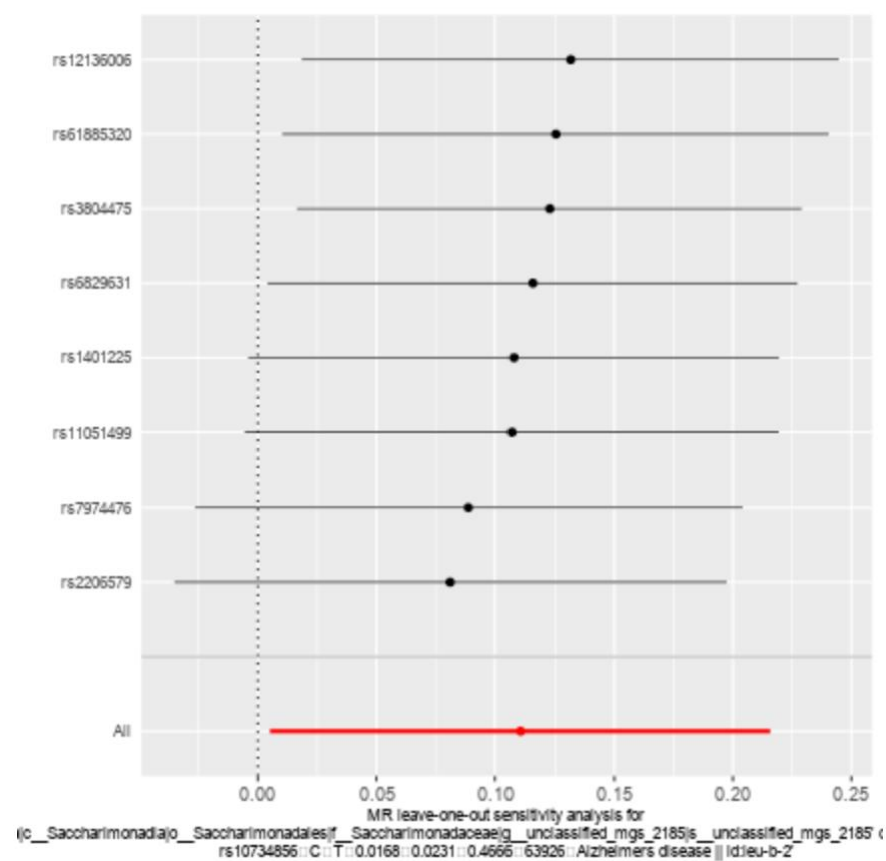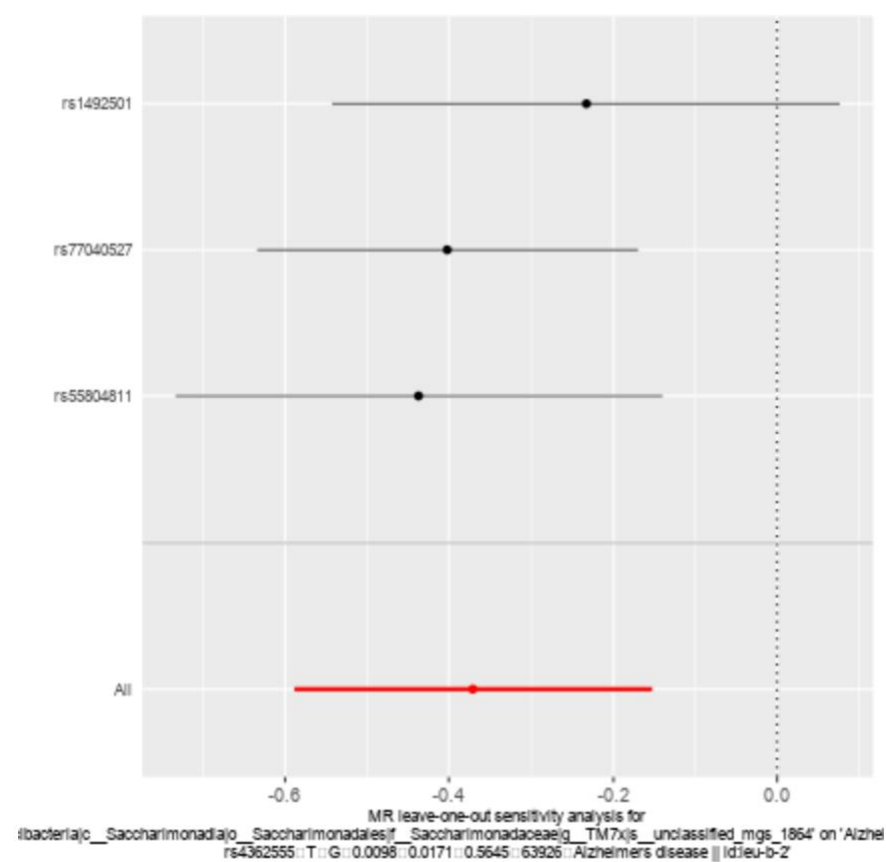

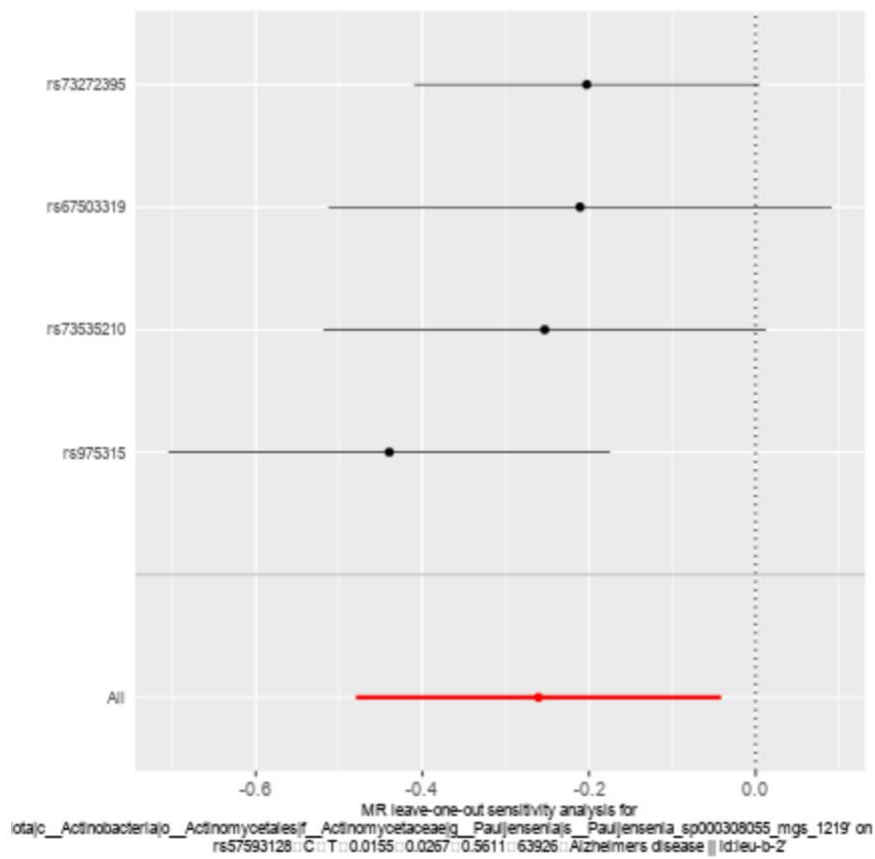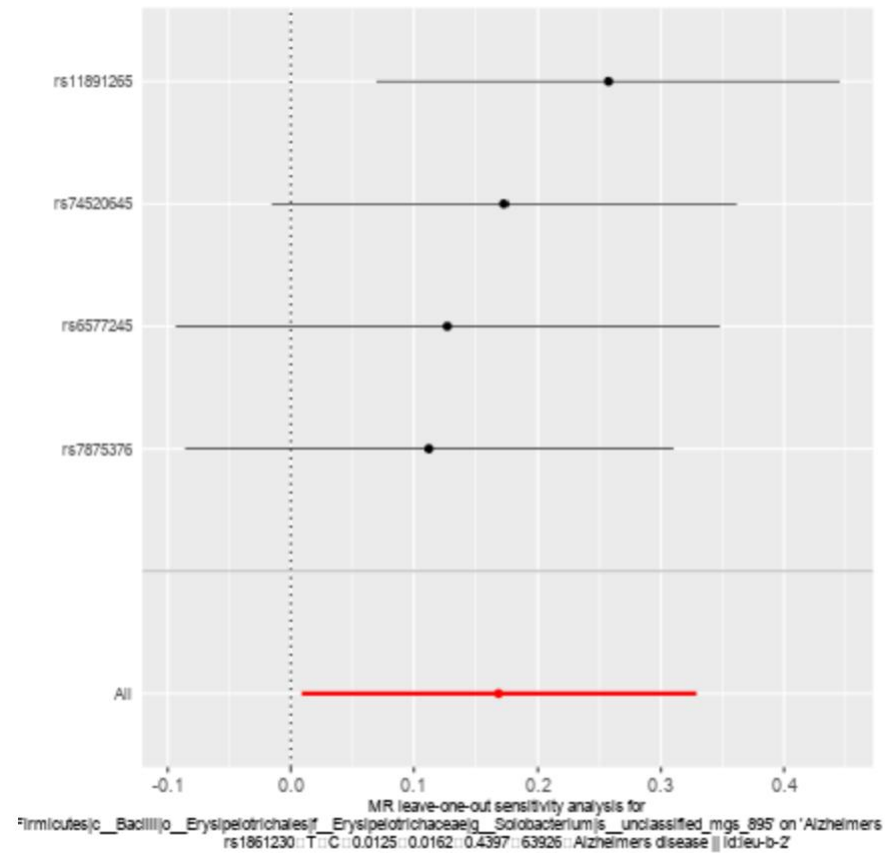

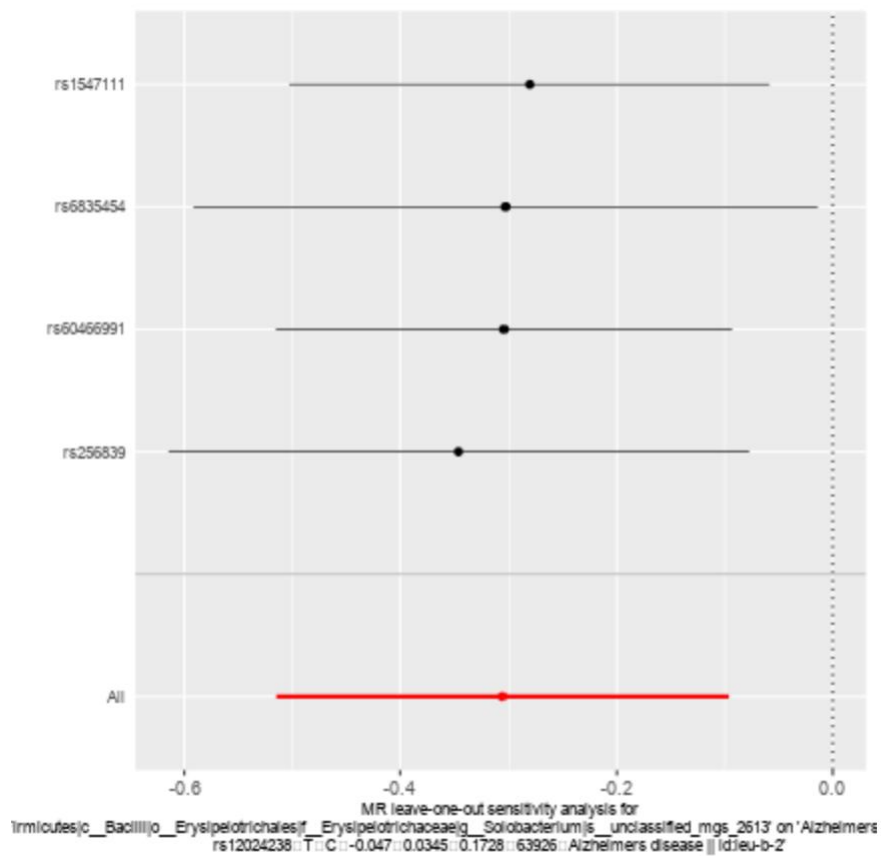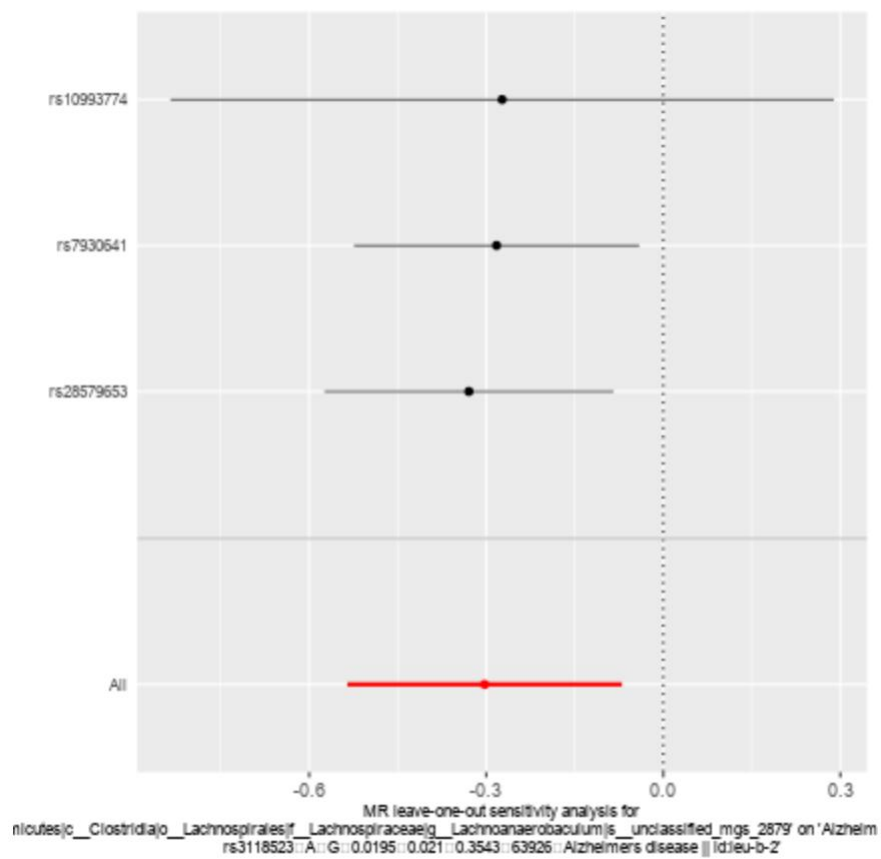

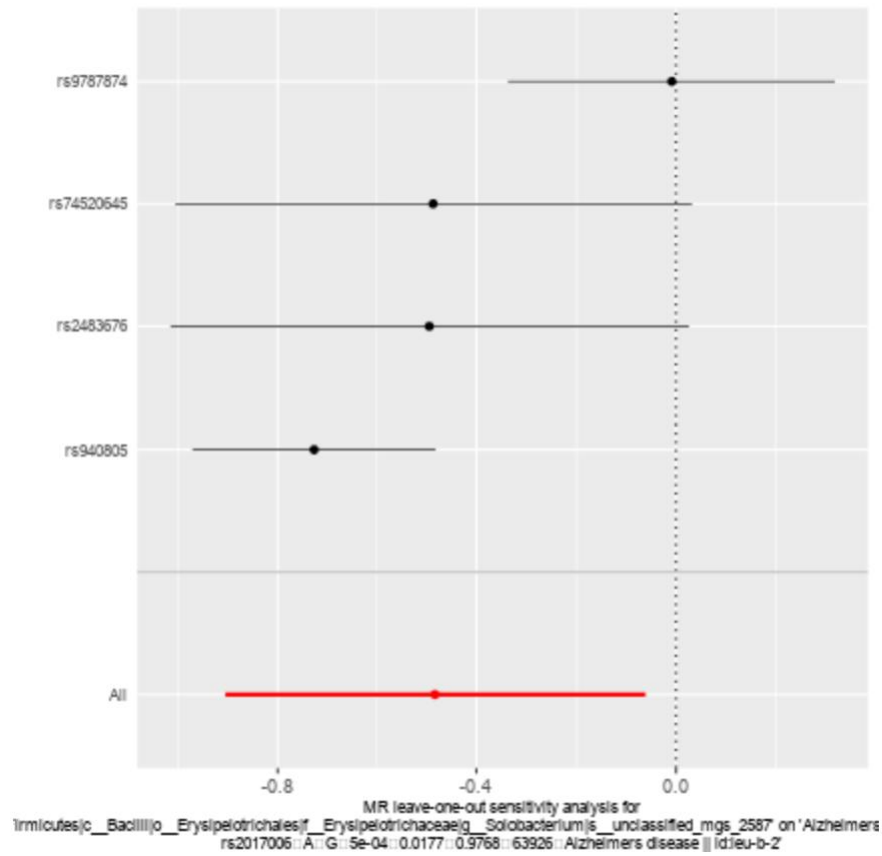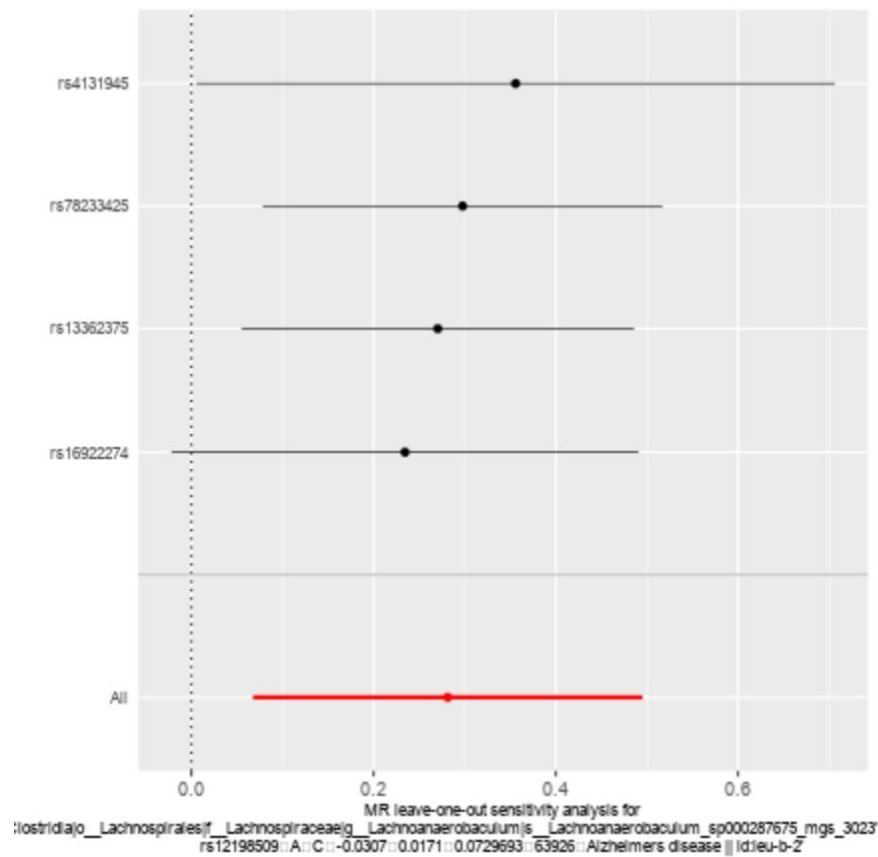

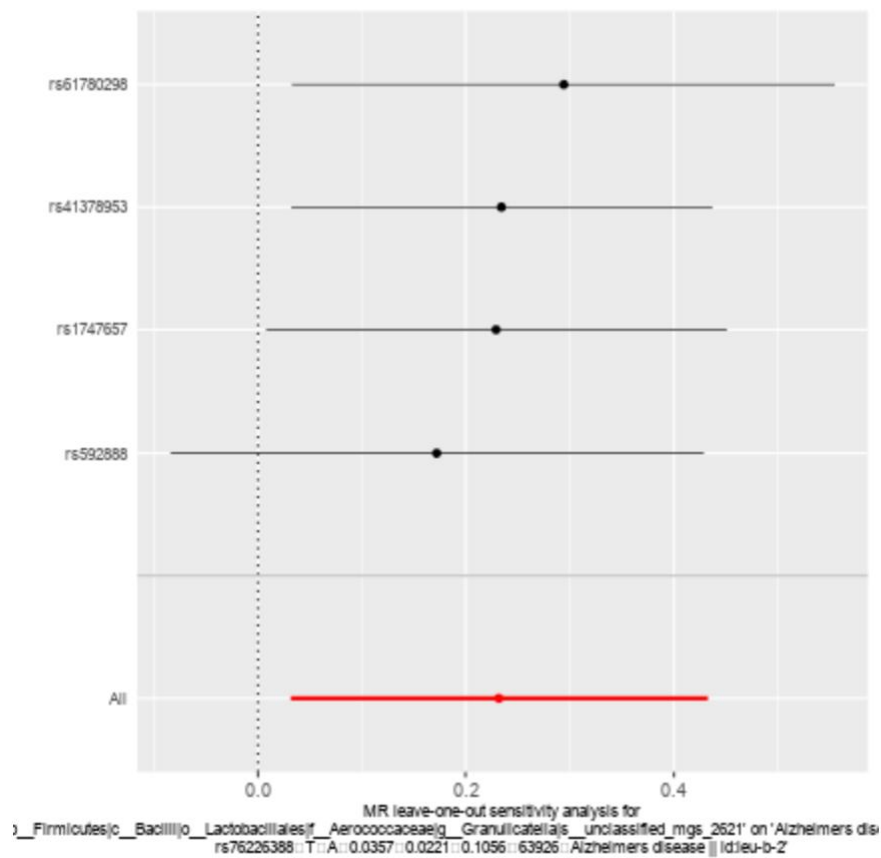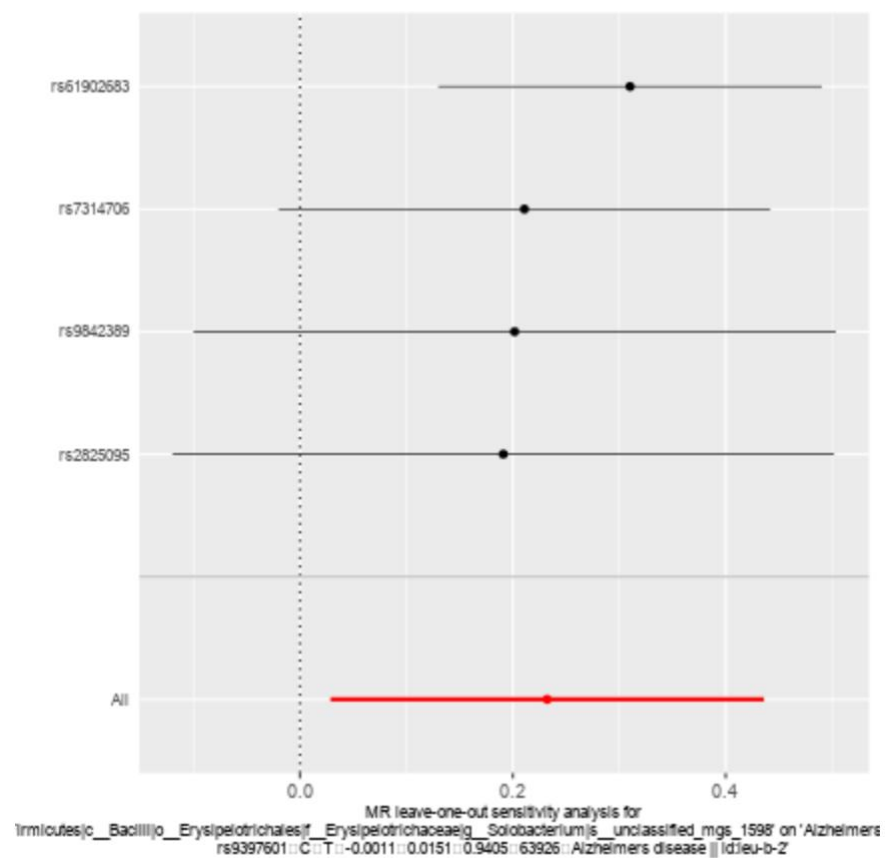

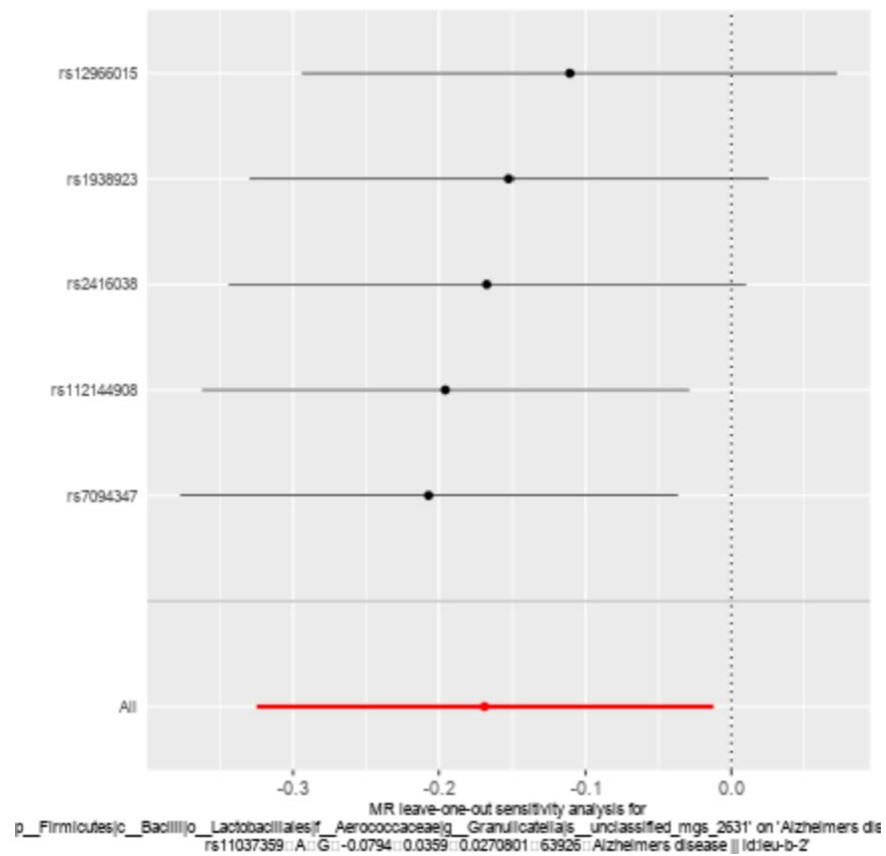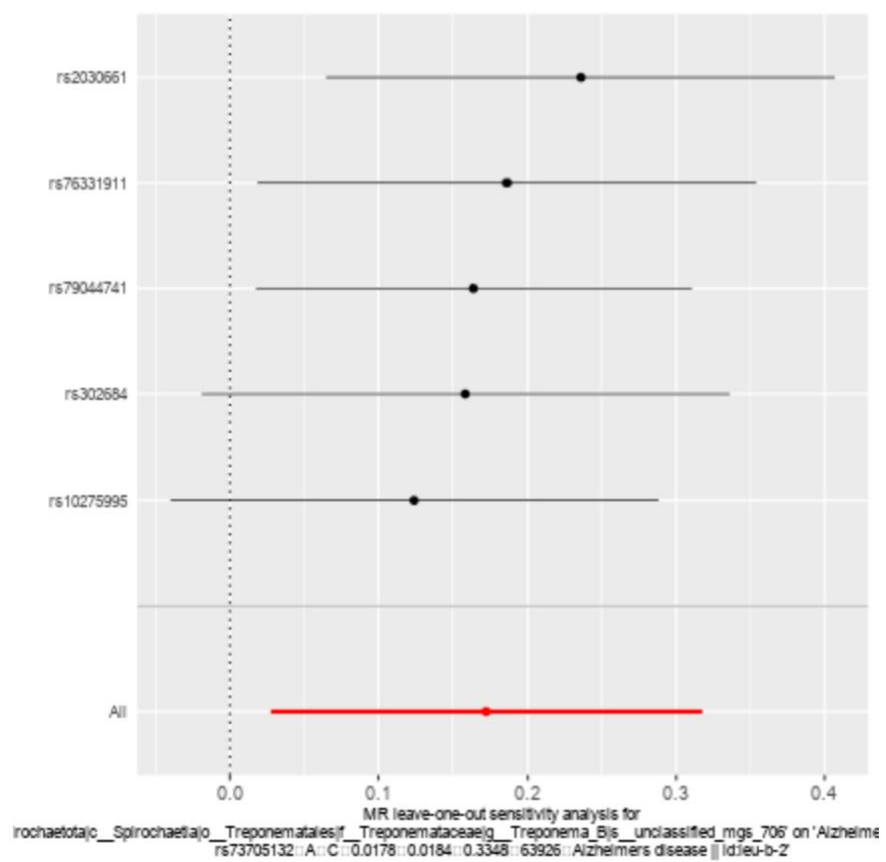

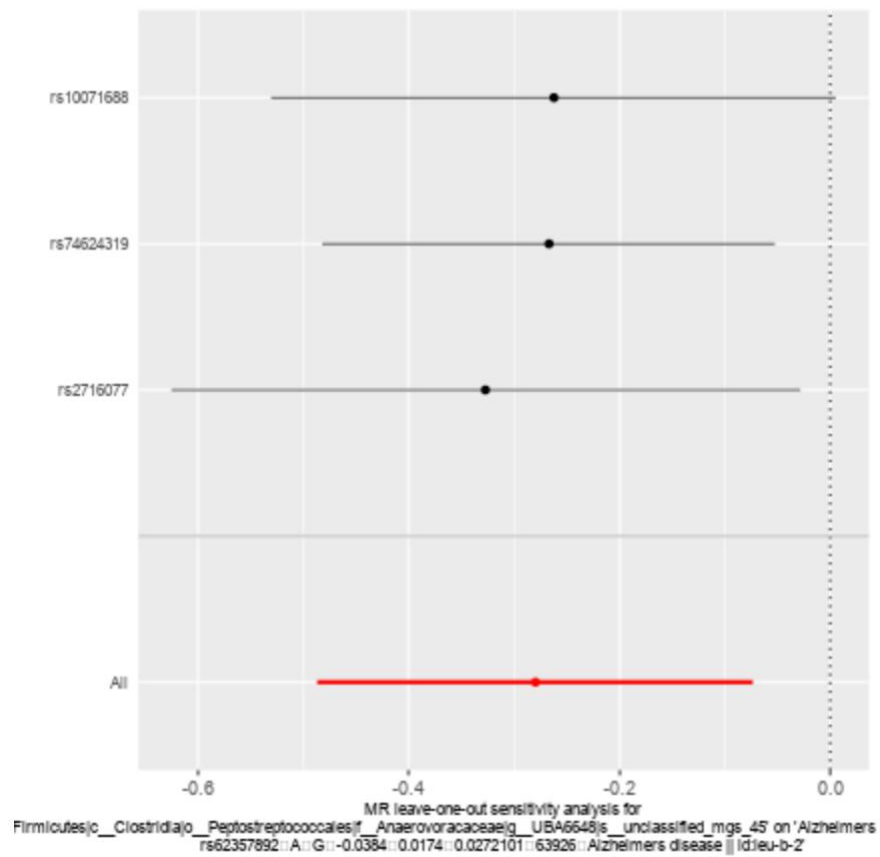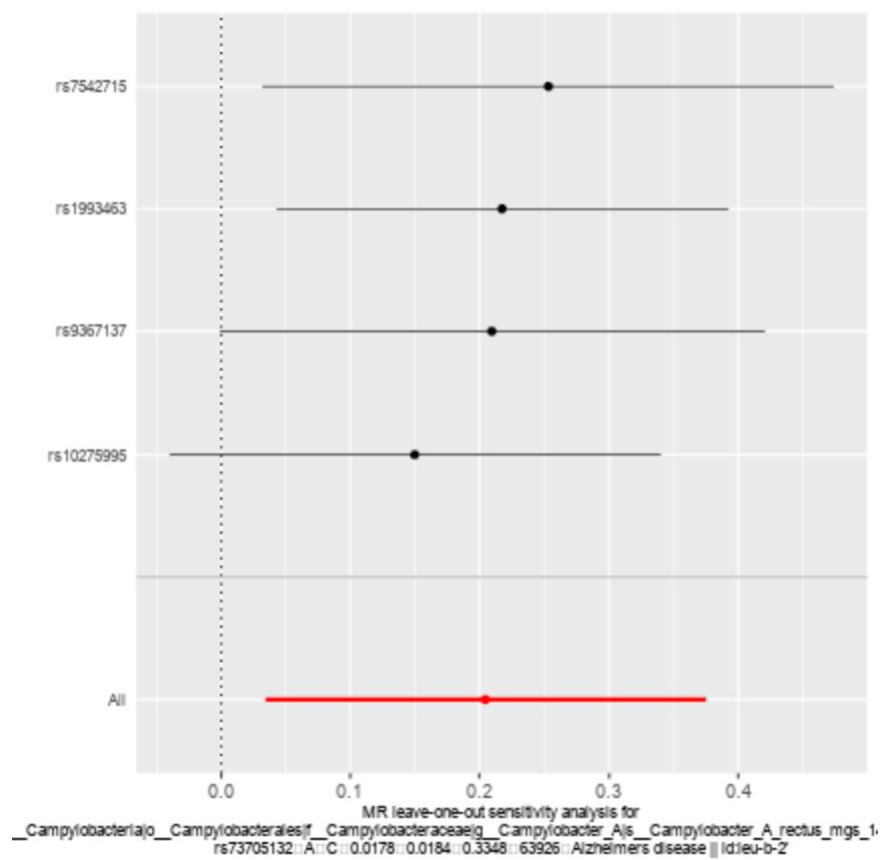

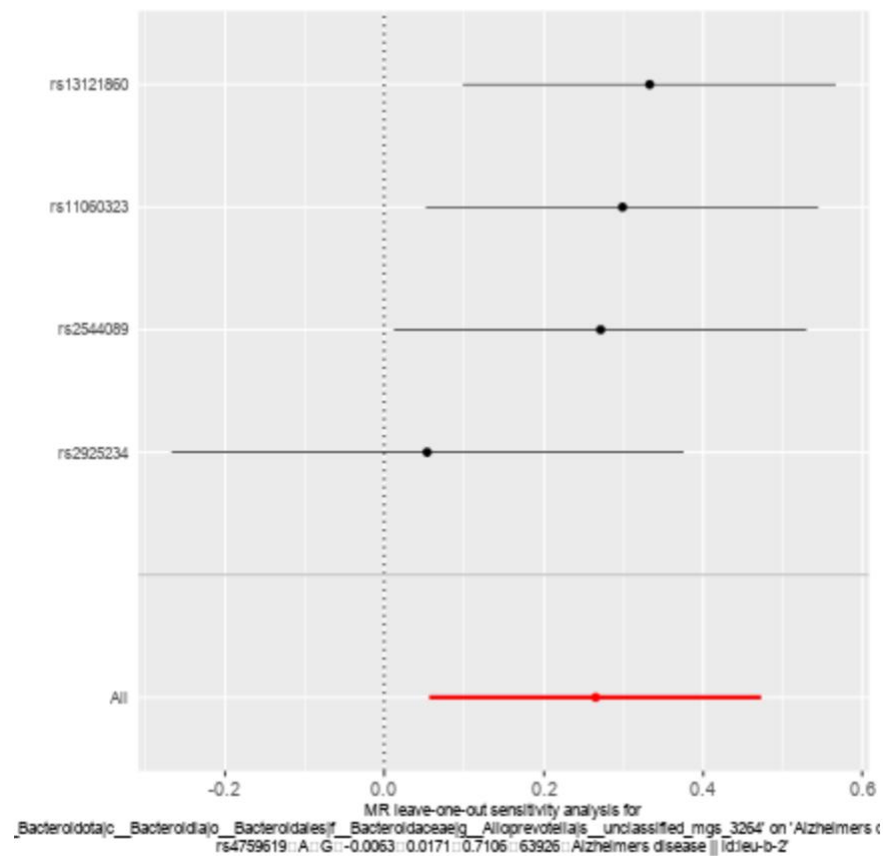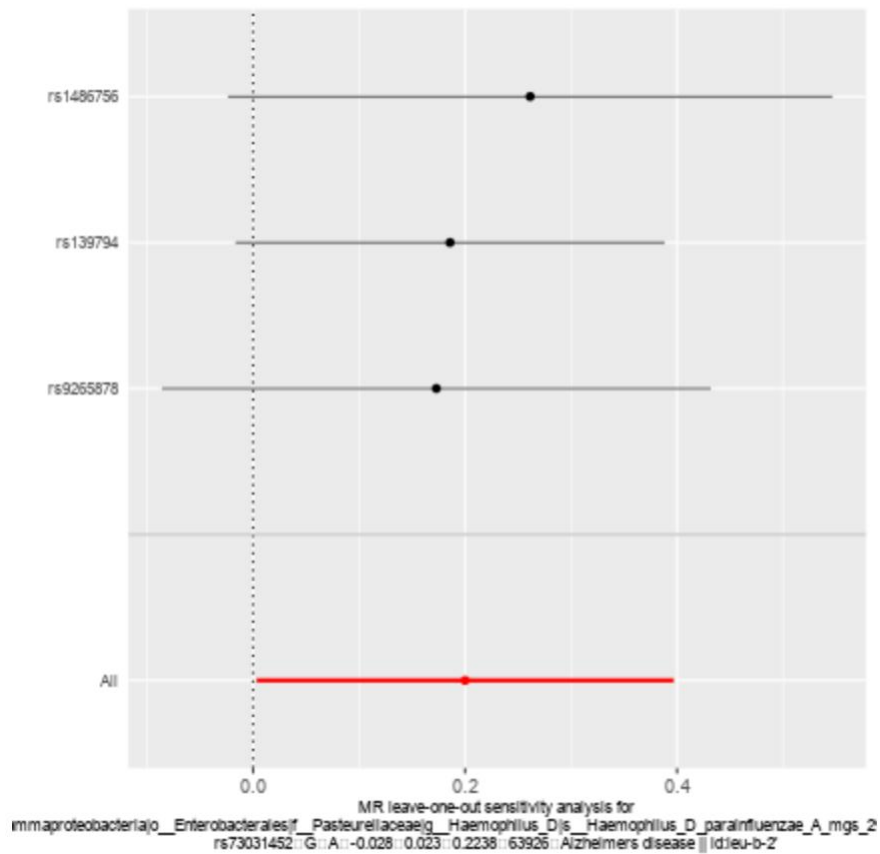

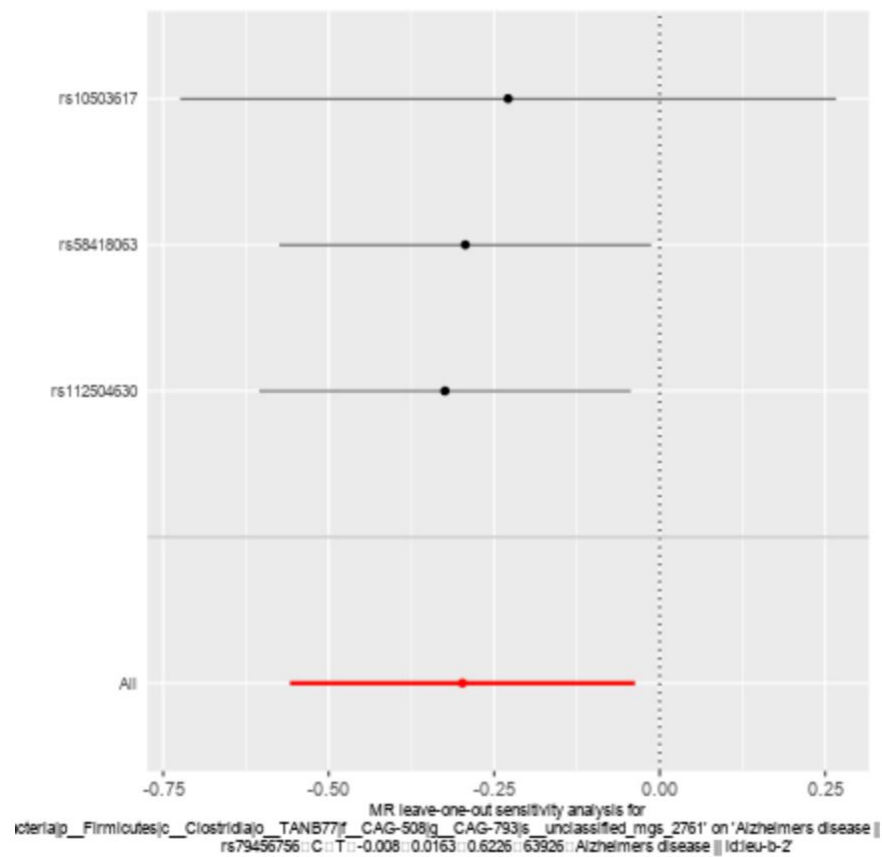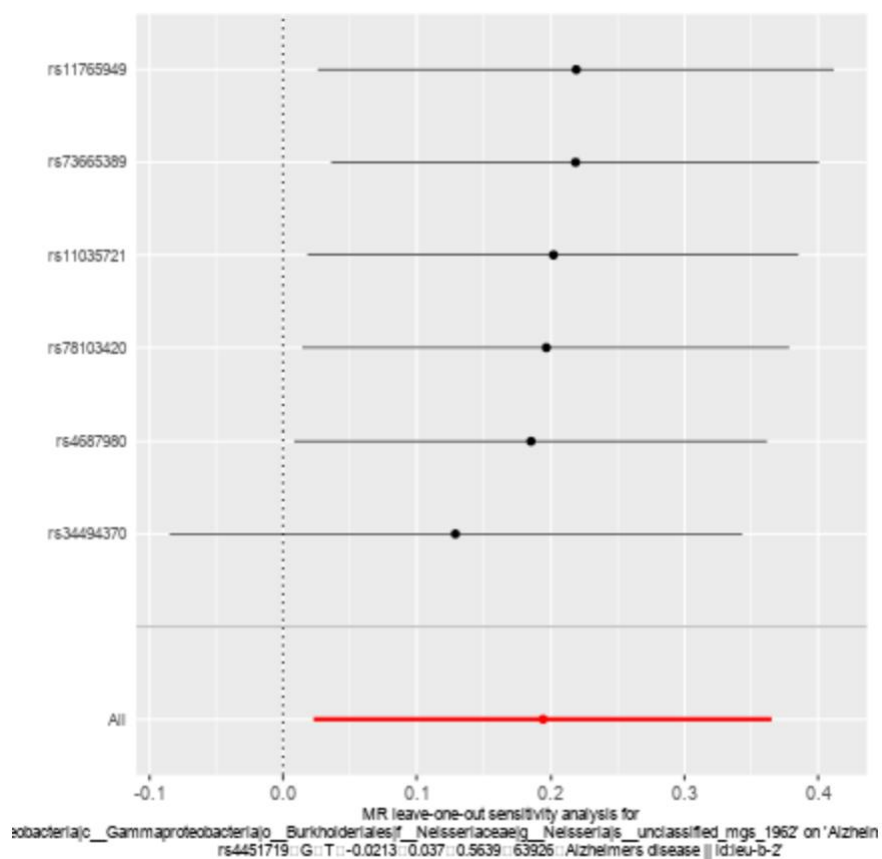

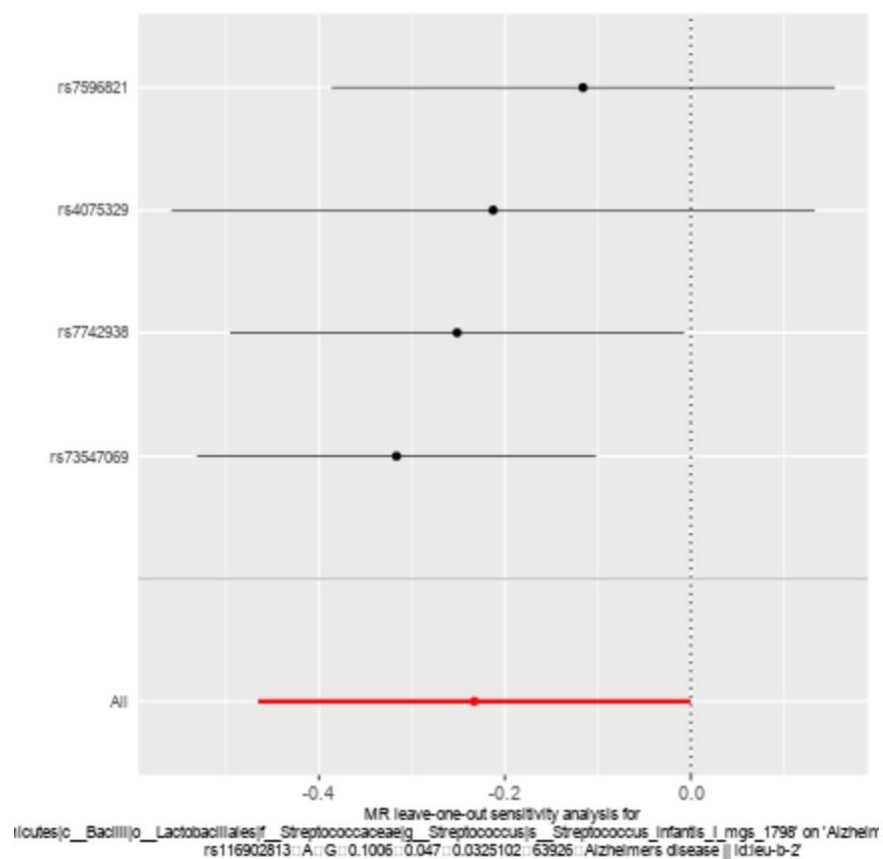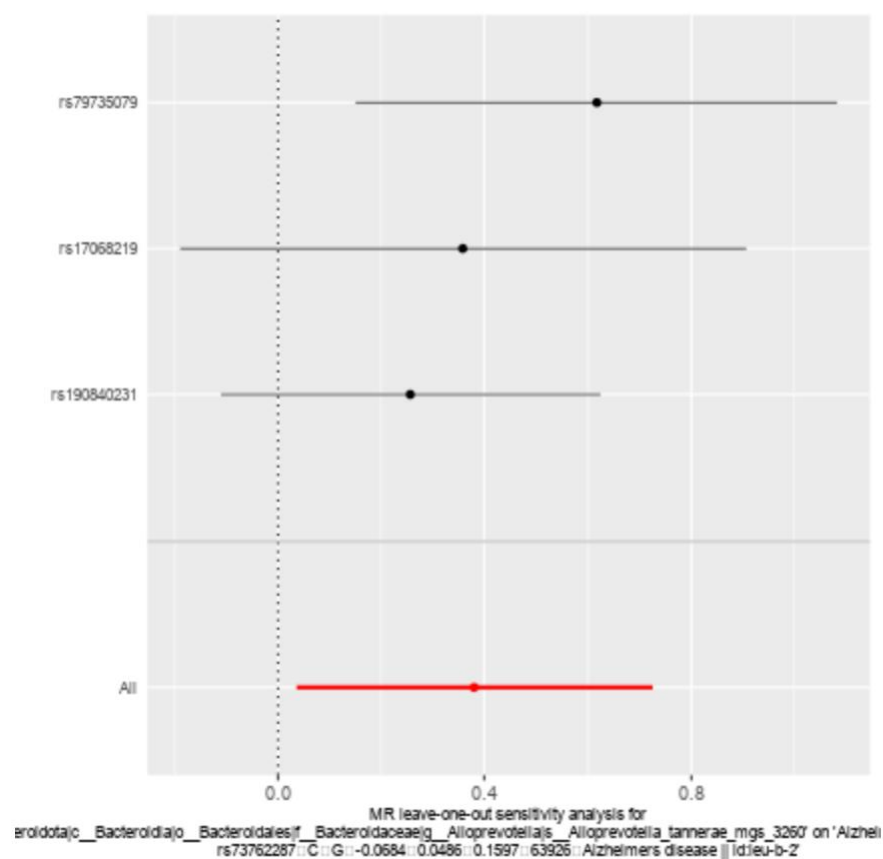

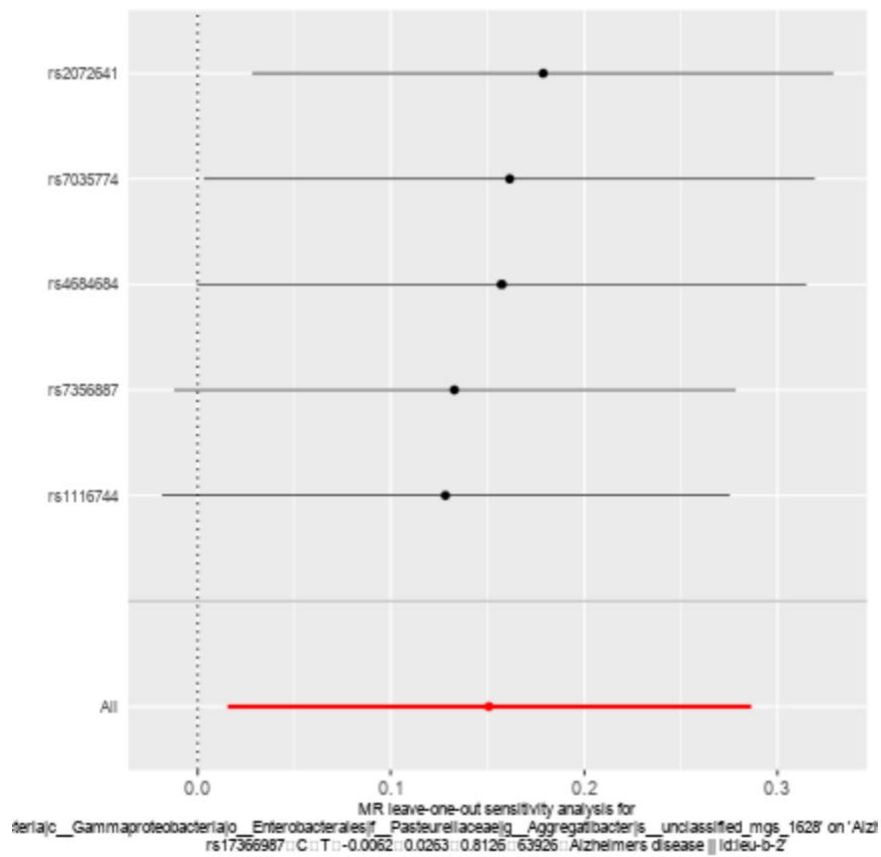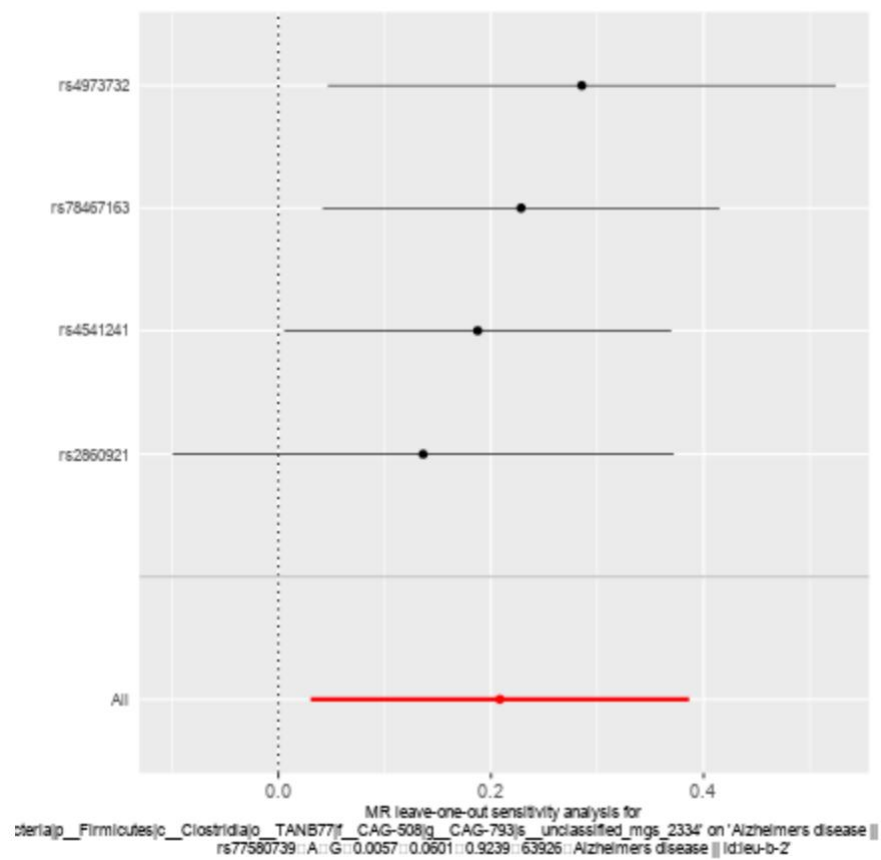

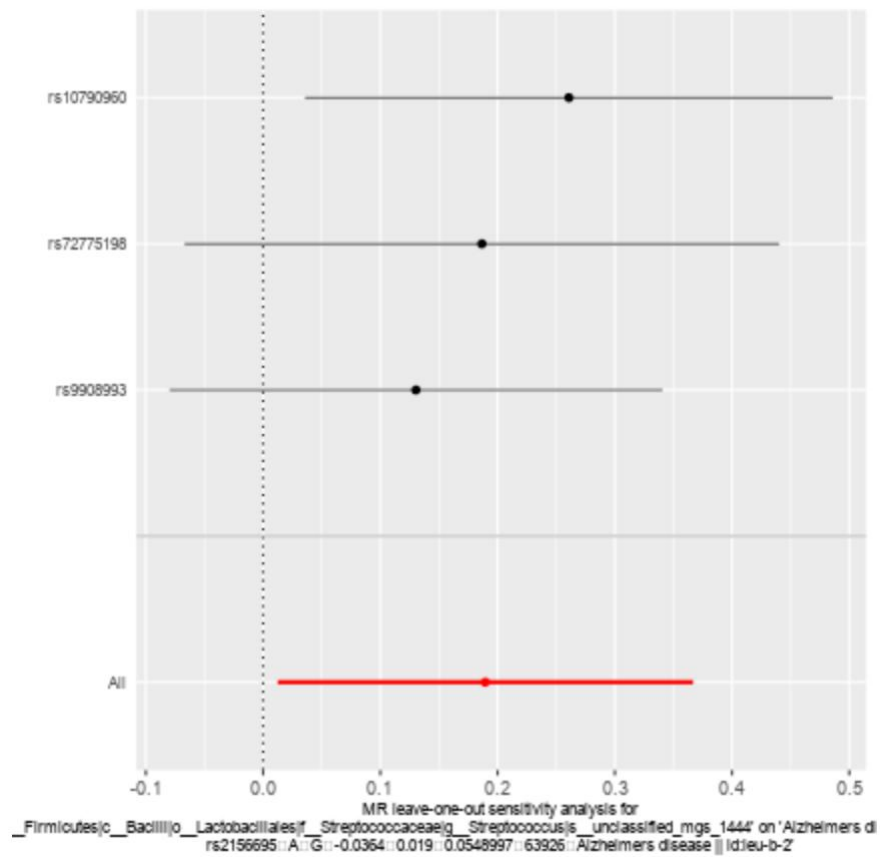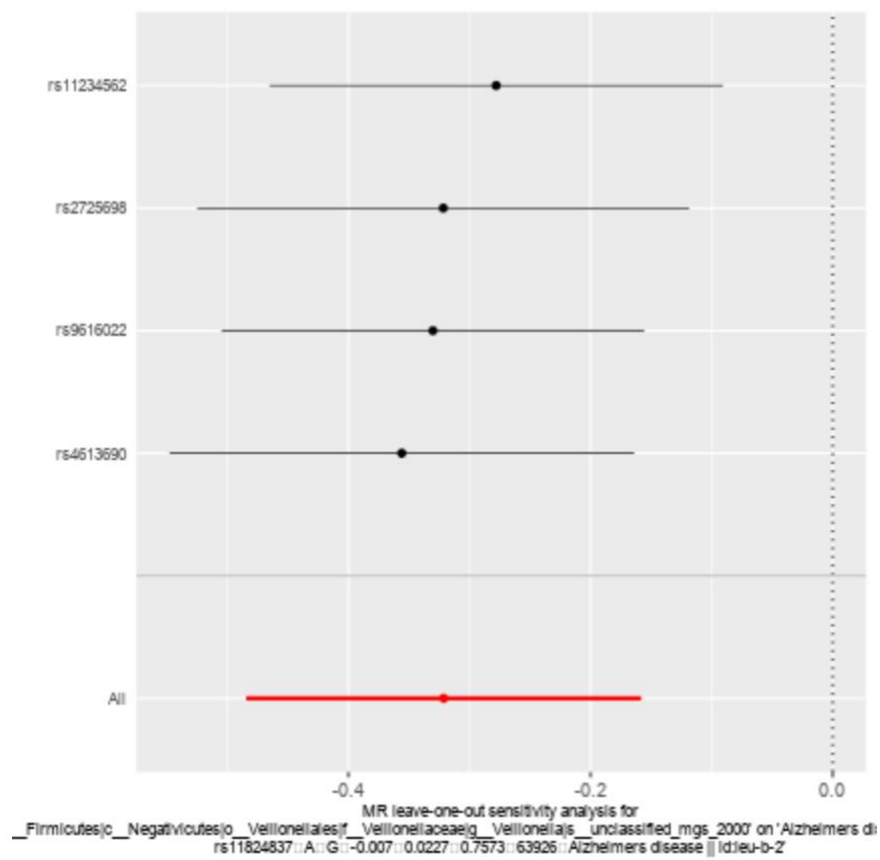

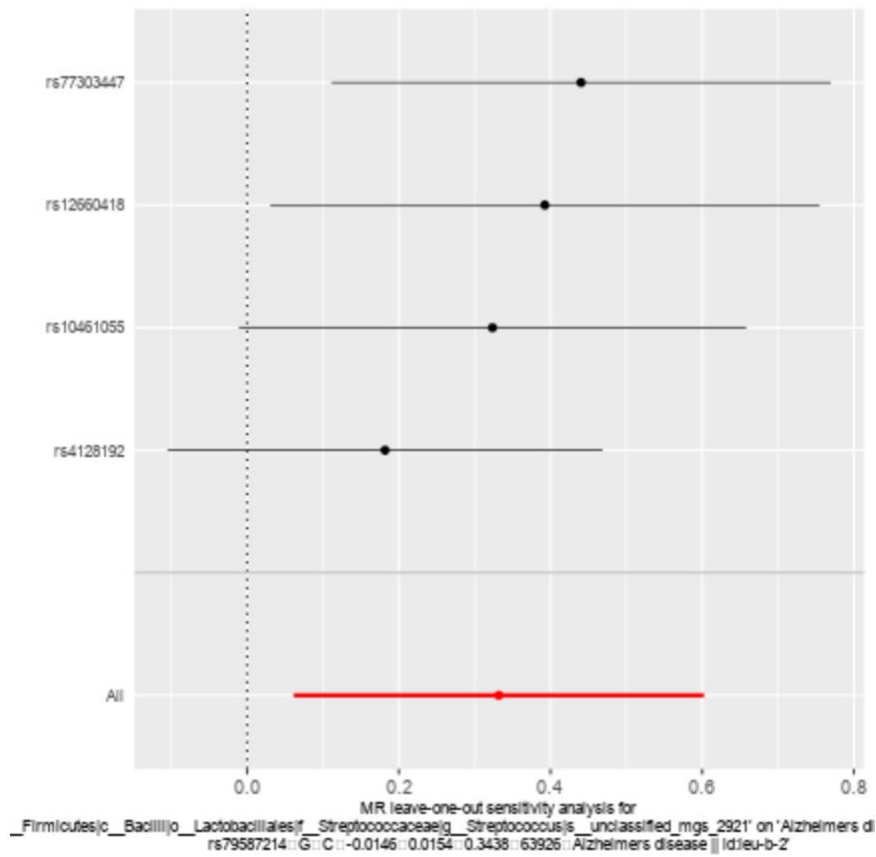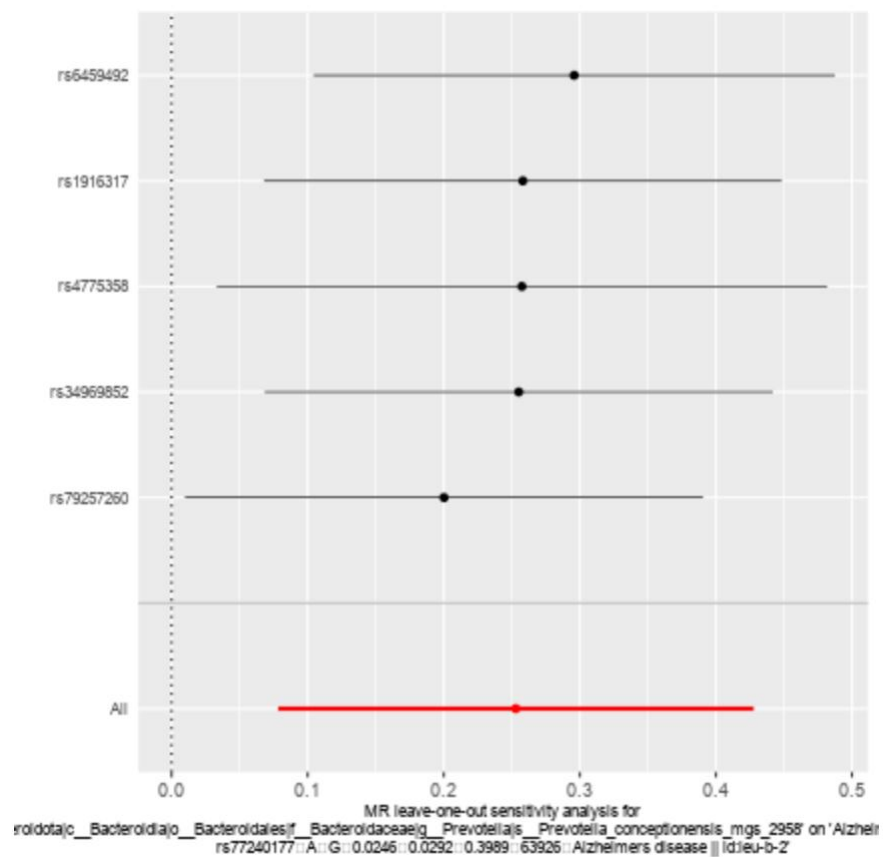

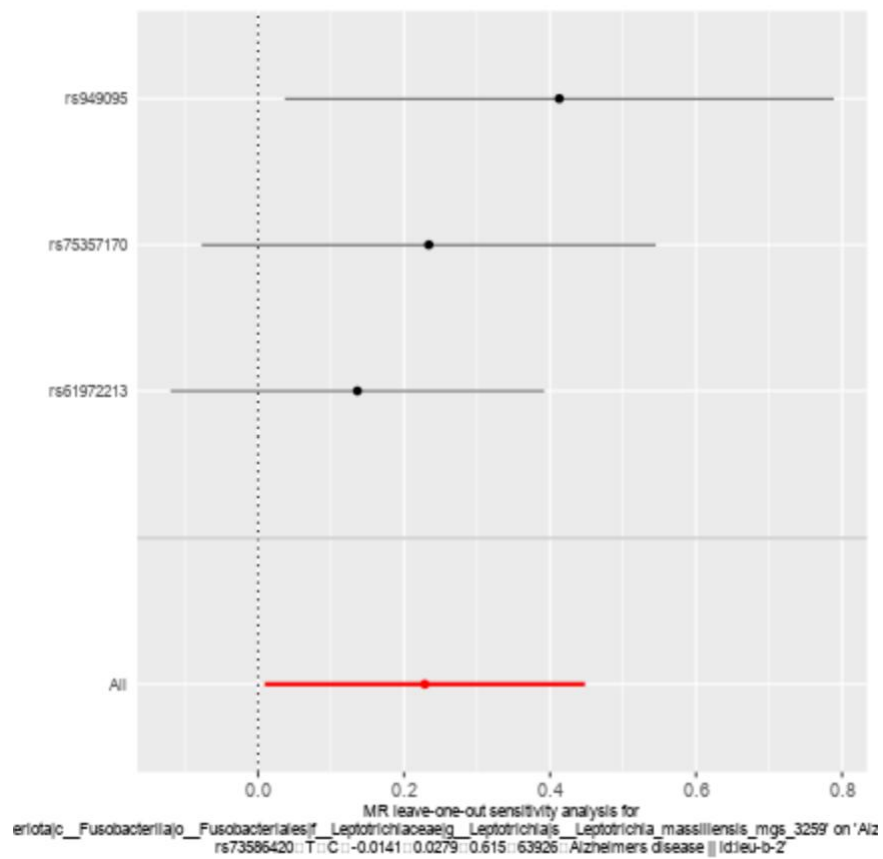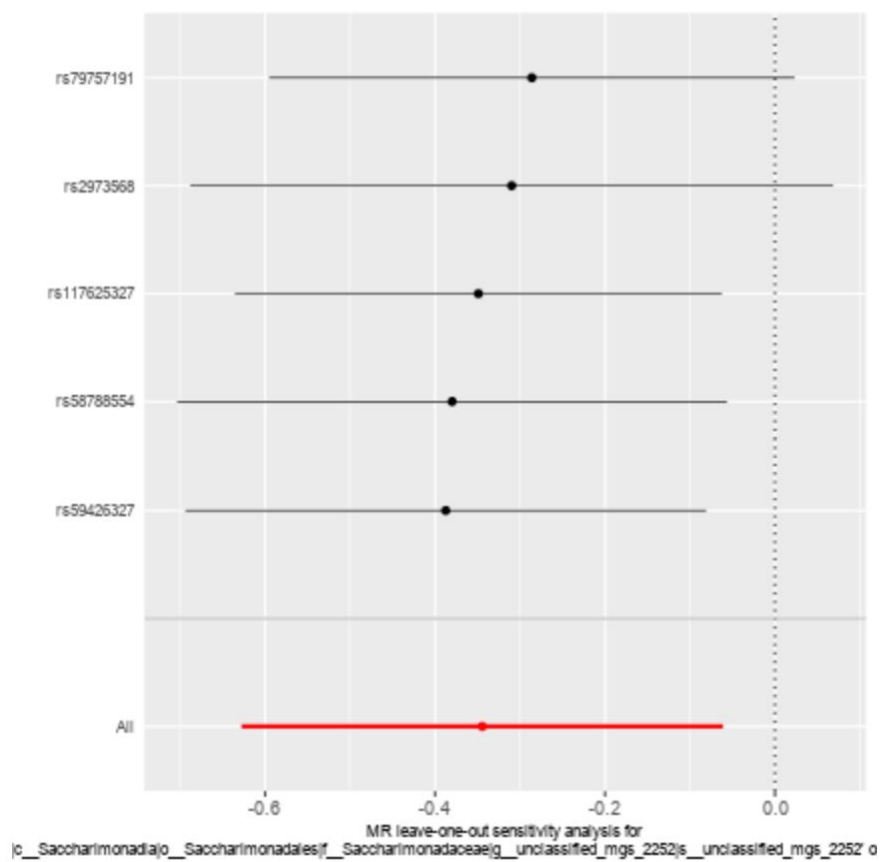

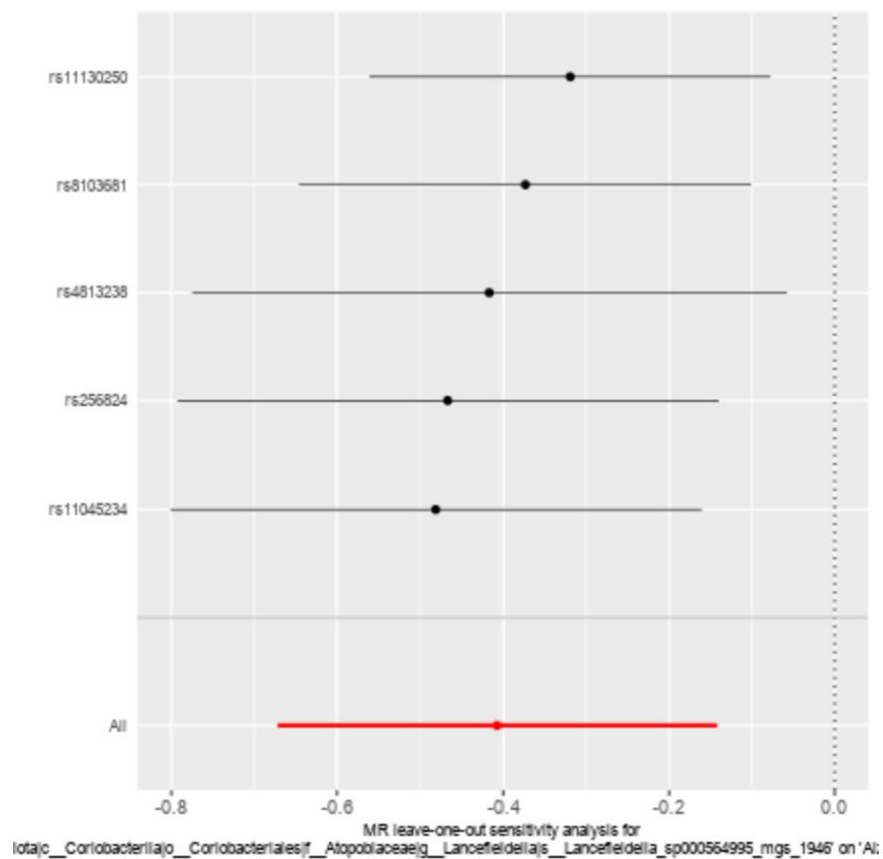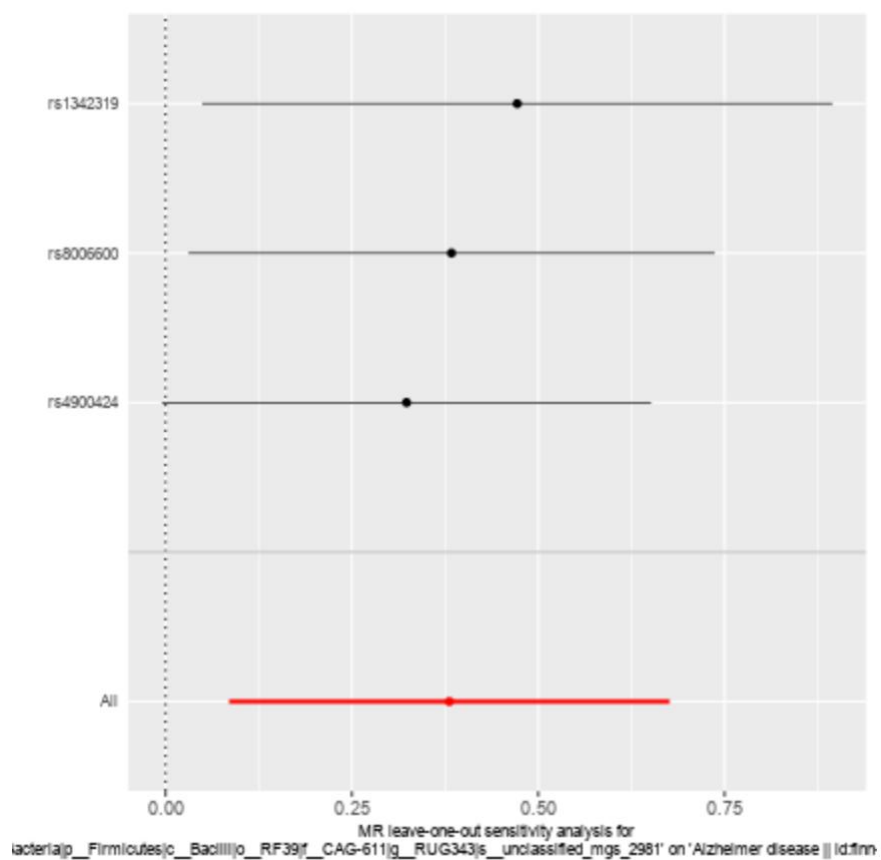

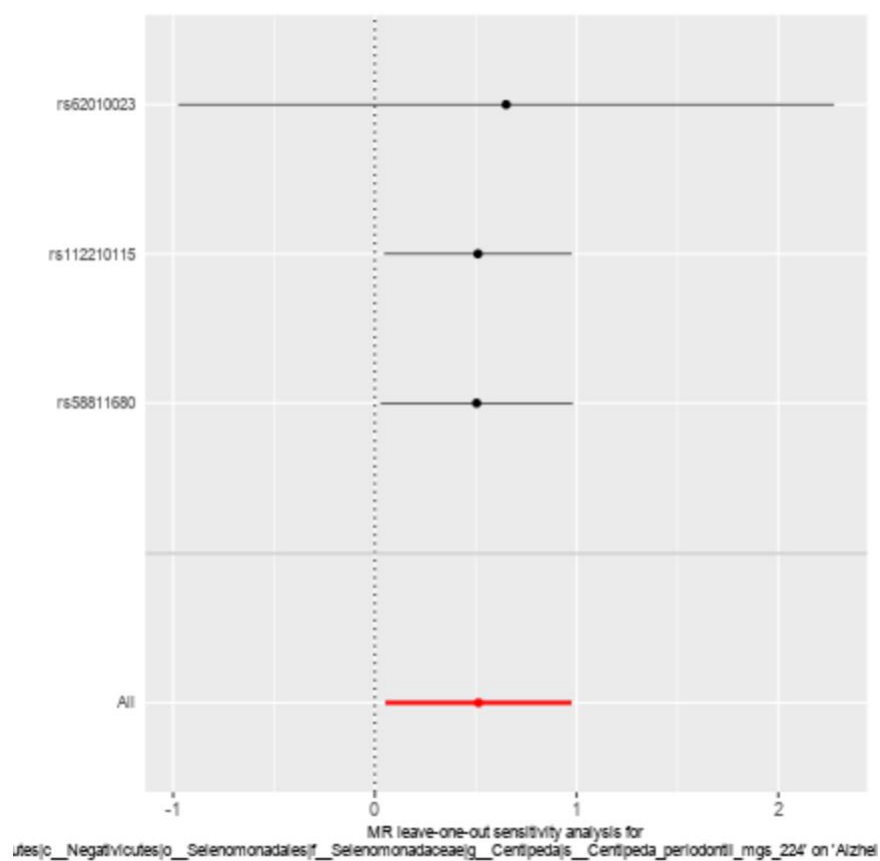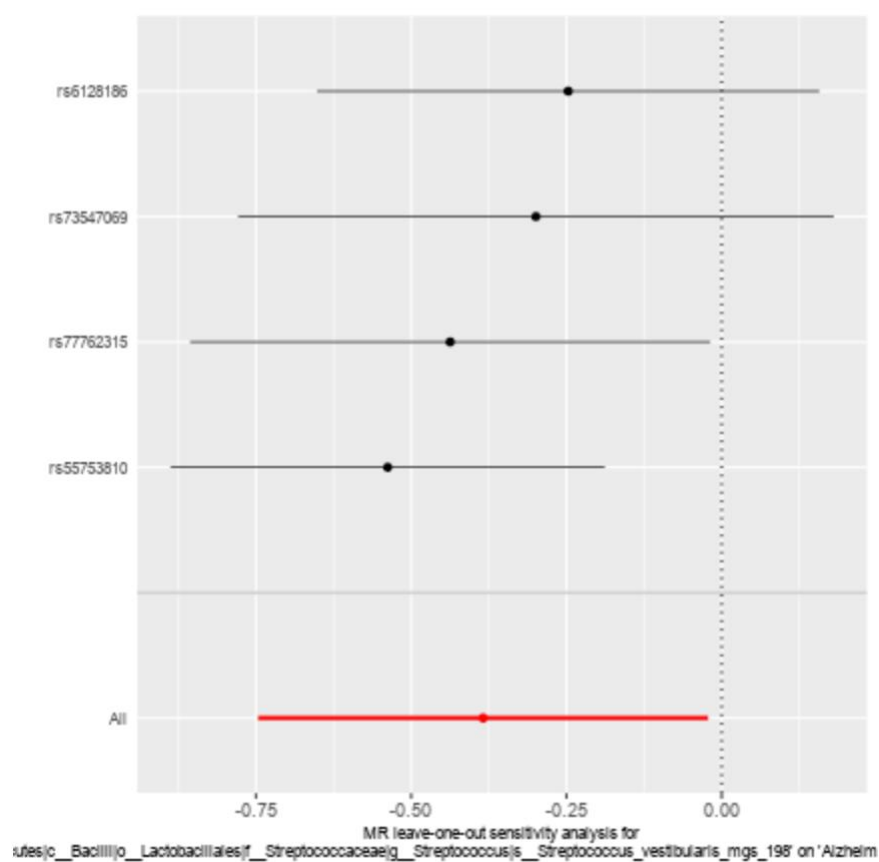

Supplement: Supplementary file 6 — Table S1‐S2 [file BRB3-15-e70753-s001.pdf]
